# Change Your Diet: How CO_2_ , Plant Phenology and Genotype Alter Grapevine Quality and Affect Performance and Larval Transcriptome of an Insect Herbivore

**DOI:** 10.1111/mec.17636

**Published:** 2025-01-09

**Authors:** Christine Becker, Christof B. Steingass, Heiko Vogel, Annette Reineke

**Affiliations:** ^1^ Department of Crop Protection Hochschule Geisenheim University Geisenheim Germany; ^2^ Department of Beverage Research Hochschule Geisenheim University Geisenheim Germany; ^3^ Department of Insect Symbiosis Max Planck Institute for Chemical Ecology Jena Germany

**Keywords:** bottom‐up effects, European grapevine moth, flavonoids, *Lobesia botrana*, phenolamides, phenolic acids, RNA‐Seq, top‐down effects, *Vitis vinifera*

## Abstract

Herbivorous insects need to cope with changing host plant biochemistry caused by abiotic and biotic impacts, to meet their dietary requirements. Larvae of the multivoltine European grapevine moth *Lobesia botrana*, one of the main insect pests in viticulture, feed on both flowers and berries. The nutritional value and defence compounds of these organs are changing with plant phenology and are affected by climate change which may accordingly alter plant–insect interactions. Here, we assessed the impacts of future elevated atmospheric CO_2_ concentrations on the host plant quality of different grapevine organs and the larval performance and the transcriptome of *L. botrana*. Using the Geisenheim VineyardFACE facility, where ‘Riesling’ and ‘Cabernet Sauvignon’ were cultivated in the field under ambient or elevated (ca. + 20%) atmospheric CO_2_ concentrations, we found that nutrient (amino acids and sugars) and defence compound (phenolic compounds) concentrations of inflorescences and ripening berries differed strongly due to plant phenology and less due to cultivar and CO_2_ concentration. Assessing global gene expression after feeding on the respective organs, we found that larval transcriptomic plasticity largely mirrored the plant biochemical plasticity. Larval relative growth rate differed between treatments in a plant phenology‐dependent manner. Grape berries contained higher amino acid concentrations and altered phenolics profiles after larval feeding. In the near future, the grapevine–*L. botrana* interaction will probably change less because of elevated CO_2_ concentrations than it does currently during one season. Changes associated with plant phenology, however, may be relevant for contemporary pest management.

## Abstract 2

see also the Perspective by Seung‐Joon Ahn

## 1 Introduction

Ever since the groundbreaking paper of Ehrlich and Raven (1964), plant specialised metabolites are regarded as key factors shaping the interaction of insects and plants. Whether a plant is a suitable host is largely determined by the insect's ability to digest the provided nutrients and detoxify encountered toxins (Breeschoten et al. 2019). However, these interactions are dynamic because plant chemical composition changes under the impact of abiotic and biotic factors, such as climate change and herbivory (Schoonhoven, Van Loon, and Dicke 2005; DeLucia et al. 2012) and, on the other hand, expression of detoxification and digestion genes in insects varies with their environment (Breeschoten et al. 2019).

Plants are the sole sources of nutrition for herbivorous insects. Their chemical composition, notably the availability of organic nitrogen, determines the nutritional quality for herbivores (Schoonhoven, Van Loon, and Dicke 2005). However, the availability of these nutrients is affected by diverse plant defence mechanisms, ranging from direct chemical and physical to indirect defences, such as interactions with natural enemies of herbivores (Schuman and Baldwin 2016). In response to these challenges of nutrient acquisition on the one hand and antinutritive or toxic compounds on the other hand, plant feeding insects possess a diverse set of enzymes to facilitate the digestion of carbohydrates, lipids and proteins as well as detoxifying enzymes (Heidel‐Fischer and Vogel 2015; Nation 2016). Both the plant nutritional value and their chemical defence levels are crucial factors for herbivore preference and performance (Rosenblatt and Schmitz 2014). Yet, plant chemical composition is far from constant and can vary considerably in space and time due to abiotic impacts like temperature, atmospheric carbon dioxide (CO_2_) concentration, water and nutrient supply, as well as biotic impacts like herbivory and plant phenology (Schoonhoven, Van Loon, and Dicke 2005). The dietary breadth of an herbivore depends to a large degree on the diversity and plasticity of the insect's digestive and detoxifying system (Breeschoten et al. 2019).

Climate change has significant potential to impact plant–insect interactions either directly by affecting plant and insect physiology or indirectly, through bottom‐up effects mediated by changes in host quality (DeLucia et al. 2012; Han et al. 2019). Rising temperatures can influence plant and insect physiology, potentially leading to higher pest pressure due to faster development, earlier spring emergence and additional generations of multivoltine insects later in the year (Martín‐Vertedor, Ferrero‐García, and Torres‐Vila 2010; DeLucia et al. 2012; Reineke and Thiéry 2016). Rising atmospheric CO_2_ concentration can enhance photosynthesis and, thereby, the plant carbon pool that provides building blocks for primary and specialised metabolites determining host plant quality (DeLucia et al. 2012; Rosenblatt and Schmitz 2014). Consequently, plants grown under elevated CO_2_ concentrations generally exhibit a lower nutritional value for herbivores and higher concentrations of defence compounds, such as ubiquitous phenolic compounds (Rosenblatt and Schmitz 2014). These indirect, plant‐mediated effects of elevated CO_2_ concentration on insects are more common than direct effects (Rosenblatt and Schmitz 2014). Depending on the specific plant response and the physiological and behavioural plasticity of the respective pest species, this will result either in higher or lower future pest pressure.

On the plant level, plant–insect interactions and the underlying mechanisms have been thoroughly studied (Dicke, van Poecke, and de Boer 2003; Stam et al. 2014; Schuman and Baldwin 2016). On the insect level, research has increased in the last decade and presented interesting results, notably on the host plant impact on gene expression patterns in herbivorous insects in laboratory experiments (Roy et al. 2016; Müller, Vogel, and Heckel David 2017; Schweizer et al. 2017; Breeschoten et al. 2019). Research gaps remain, however, regarding global, RNA‐Seq‐based gene expression analyses of non‐model insect species, especially in field experiments and on the impact of climate change.

The European grapevine moth, *Lobesia botrana* Denis & Schiffmüller (Lepidoptera: Tortricidae), is a polyphagous pest with the capacity to inflict serious damage in vineyards. It is currently the major insect pest in European vineyards and is invading wine growing regions worldwide (Ioriatti et al. 2011; Gutierrez et al. 2012; Benelli et al. 2023a). The larvae of this multivoltine insect feed on substantially different phenological stages of grapevine reproductive organs, from flowering to harvest ripe grapes (Ioriatti et al. 2011). The inflorescence‐feeding first generation and the following berry‐feeding generations therefore feed on grapevine tissues that vary morphologically, histologically and chemically (Ioriatti et al. 2011; Vincent et al. 2012). Although the concentrations of nutrients and defence compounds are well known in berries of different ripening stages (Vincent et al. 2012), the chemical composition of grapevine inflorescences remains understudied (Cuadros‐Inostroza et al. 2016). The transcriptomic plasticity exhibited by larvae of subsequent generations in response to drastic changes in the host plant organs has not been studied yet. Polyphagous herbivores adapt their metabolism and enzymatic machinery to acquire nutrients and detoxify plant defences when changing host plant species, accompanied by transcriptomic changes (Müller, Vogel, and Heckel 2017). Hence, transcriptomic plasticity may increase with the breadth of the dietary range (Roy et al. 2016). A similar degree of transcriptomic plasticity may be required by multivoltine insects to cope with changes associated with plant phenology.

Previously, we analysed grapevine gene expression showing an impact of elevated CO_2_ concentrations, plant phenology and *L. botrana*‐herbivory (Reineke and Selim 2019). Here, we are adding another trophic level, examining the effect of elevated CO_2_ concentrations, grapevine cultivar and plant phenology on grapevine chemical host quality as well as on *L. botrana* larval gene expression and development. The following hypotheses were investigated:

1. The concentrations of nutrients and defence compounds in grapevine reproductive organs are affected by atmospheric CO_2_ concentration, grapevine phenological stage and cultivar.
2. Larval feeding of *L. botrana* affects the nutritional value and/or defence compound concentration of grapevine reproductive organs in their favour, that is, larval feeding increases nutrient concentrations and suppresses host plant defences.
3. The transcriptome of *L. botrana* larvae is affected by changed concentrations of nutrients and defence compounds in grapevine reproductive organs due to grapevine phenological stage, grapevine cultivar and elevated atmospheric CO_2_ concentrations.

To this end, we first measured the concentration of nutrients (sugars and amino acids) and defence compounds (phenolic compounds) for two grapevine cultivars (‘Riesling’ and ‘Cabernet Sauvignon’) in reproductive organs of two phenological stages (inflorescences and berries at onset of ripening: véraison), cultivated under ambient or elevated CO_2_ concentrations in the Geisenheim VineyardFACE (free‐air carbon dioxide enrichment) facility as well as the impact of larval herbivory on the concentrations of the respective compounds. We then studied *L. botrana* larval development and transcriptomic plasticity after feeding on plants from the respective CO_2_ concentrations, cultivars and phenological stages, representing the first and second generations of *L. botrana*. The larval transcriptome was analysed using high throughput sequencing (RNA‐Seq) and *de novo* transcriptome assembly which was verified using quantitative reverse transcription‐PCR (qPCR). Here we report on the first larval transcriptomic study of the non‐model species *L. botrana* under field conditions.

## 2 Materials and Methods

### 2.1 Insects

Experiments were conducted with *L. botrana* larvae derived from a laboratory strain laboratory strain maintained at Hochschule Geisenheim University, Germany. To mitigate inbreeding, the laboratory colony is regularly supplemented with additional *L. botrana* genotypes either collected from vineyards or received from other laboratory strains outside Geisenheim. Larvae raised in the lab can differ from larvae in wild populations (Vogelweith et al. 2014) with regard to physiology but they allow for the reduction of larval transcriptional variation because of different histories. Larvae were cultured in groups in plastic boxes (20 × 15 cm and 9 cm height) in an insect rearing room (24°C ± 1°C, 40% ± 12% relative humidity, light/dark photoperiod: 16:8 h) and were fed *ad libitum* with a modified semi‐synthetic diet based on the general‐purpose diet of Singh and Moore (1985). In brief, agar and alfalfa sprouts were mixed and boiled, and sucrose, yeast, wheat germ, cholesterol, casein, sunflower oil and Wesson's salt mixture were added. A separately prepared mixture of vitamins, sorbic acid, propionic acid and 95% ethanol was combined with the diet after cooling. Third instar larvae (L3; assessed visually) were used in the experiments described below.

### 2.2 Experiments

#### 2.2.1 Vineyard FACE Experiment

The Geisenheim VineyardFACE was established in a Hochschule Geisenheim University vineyard (49°59′ N, 7°57′ E; 96 m above sea level) in 2011 and consists of six ring‐frame structures each with an inner diameter of 12 m. Three rings are under elevated CO_2_ (eCO_2_) and three under ambient CO_2_ (aCO_2_) concentration (Figure S1). Each ring contains seven rows of *Vitis vinifera* L. ‘Riesling’ and ‘Cabernet Sauvignon’ grapevine plants, totalling 64 grapevine plants per ring and variety. For a detailed description of the Geisenheim VineyardFACE facility refer to publications by Reineke and Selim (2019) and Wohlfahrt et al. (2018). During the experimental periods described here, from (A) 29 May to 6 June and (B) 9–17 August 2018, the average CO_2_ concentrations in the three ambient rings were 409 ± 22 ppm and 409 ± 25 ppm (mean ± standard deviation) respectively. In the eCO_2_ rings, air was enriched during daylight hours to approximately 18%–19% above the ambient CO_2_, that is, 487 ± 22 ppm for the June and 483 ± 27 ppm for the August experimental period respectively. These 18%–19% correspond to the increase in CO_2_ concentration in the near future, predicted for 2050 by the Intergovernmental Panel on Climate Change (Ciais et al. 2013). CO_2_ concentrations were closely monitored by two LI‐8100 analyser control units in the grapevine canopy. Additional details regarding weather conditions and CO_2_ concentrations are presented in Table S2 and Figure S3A,B.

Three factors were considered in this experiment: (1) two CO_2_ concentrations approximately 409 and 485 ppm, (2) two grapevine cultivars ‘Riesling’ and ‘Cabernet Sauvignon’ and (3) two grapevine developmental stages ‘inflorescences’ and ‘berries at the onset of ripening: véraison’. These factors resulted in eight different treatments: ‘Riesling’ inflorescences and véraison berries, ‘Cabernet Sauvignon’ inflorescences and véraison berries, at ambient and elevated CO_2_ concentrations, respectively, with three replicates per treatment. We used the means per ring for statistical evaluation to reduce single‐larvae and single‐plant related variability and to focus on the CO_2_‐related effects.

For each replicate, six inflorescences or véraison berry‐bunches per cultivar and per FACE ring were infested with six *L. botrana* larvae and covered by a nylon mesh bag (12 × 16 cm). After 7 days, the inflorescences or bunches were transferred to the lab and larvae were carefully recovered and flash‐frozen in liquid nitrogen, followed by storage at −80°C until RNA extraction. Larvae were weighed before and after the experiment to assess their relative growth rate (RGR) calculated as biomass gained/[(initial biomass) × (day)] as described by Stiling and Cornelissen (2007).

### 2.3 Chemical Composition of Grapevine Reproductive Organs

Inflorescences and véraison berries with clear signs of larval feeding as well as undamaged véraison berries were harvested on the same day the larvae were collected from the VineyardFACE, flash‐frozen in liquid nitrogen and stored at −80°C until freeze‐drying. Dry material was ground to a homogeneous powder using a ball mill before extractions.

#### 2.3.1 Amino Acids

Amino acids were extracted from 230 and 180 mg of dried, ground plant material per inflorescences and véraison berries sample, respectively, using 2 mL lithium citrate extraction buffer (SYKAM, Eresing, Germany). After sonification (Bandelin Sonorex super RK 1028) for 30 min, samples were centrifuged (Heraeus Sepatech, Megafuge 1.0) for 15 min at 4000 rpm and 4°C. Supernatants were filtered with syringe filters (Titan3 cellulose acetate, 0.45 μm, 17 mm diameter; msscientific) and analysed using ion‐exchange chromatography and post‐column derivatisation with ninhydrin (SYKAM, Eresing, Germany) on an automated amino acid analyser (ARACUS advanced, membraPure, Henningsdorf, Germany; containing a cation exchange column). Ninhydrin derivatives were detected at 570 and 440 nm. L‐norleucine (100 nMol ml^−1^; Sigma Aldrich No. N8513) was used as technical control. Identification and quantification were based on retention times and peak areas compared to authentic reference standards (SYKAM, Eresing, Germany; Standard PH Cat. No. 6006002 and PH‐S Cat. No. 6006005). We analysed L‐arginine, L‐histidine, L‐methionine, L‐tryptophan, L‐threonine, L‐valine, L‐leucine, L‐isoleucine, L‐phenylalanine, L‐lysine, L‐tyrosine and L‐proline as they are considered essential for many insect species (Barbehenn, Reese, and Hagen 1999; O'Brien, Fogel, and Boggs 2002) and L‐serine, L‐glycine and L‐alanine because they are important compounds of insect silk which plays a role in Lepidopteran larval defence (Sutherland et al. 2010).

#### 2.3.2 Sugars

D‐glucose, D‐fructose and sucrose were extracted from 10 mg dried, ground plant material using 1.6 mL water as extraction solvent and their concentrations were measured using an enzymatic kit (Roche Diagnostics/ R‐Biopharm, Darmstadt, Germany). For extraction, 800 μL of water were added, incubated at 78°C for 20 min in a water bath (P‐D Industriegesellschaft mbH, Dresden, Germany), and centrifuged for 5 min at 14,000 rpm and 4°C (centrifuge 5425 R; Eppendorf, Hamburg, Germany). The supernatant was collected and the pellet was extracted twice with 400 μL water. Supernatants were combined, centrifuged at 14,000 rpm and 4°C, and 100 μL were used for analysis following the manufacturer's protocol.

#### 2.3.3 Phenolic Compounds

Phenolic compounds were measured following established methods (Friedel et al. 2015; Strack and Stoll 2021). In brief, dried, ground plant material (inflorescences: 100 mg, ‘Riesling’ véraison berries: 100 mg; ‘Cabernet Sauvignon’ véraison berries: 200 mg) was extracted using acidified acetonitrile under SO_2_ protection. After sonification for 30 min, extracts were centrifuged, the supernatant collected, and the pellet extracted again. Combined supernatants were reduced to 2 mL, stored at 4°C and analysed within 3 days. Compounds were identified via HPLC‐DAD‐ESI(−)‐QTOF‐HR‐MS and ESI(−)‐MS^*n*^ analyses and quantified by HPLC‐DAD (for technical details and identification of individual phenolic compounds please refer to Supporting Information Methods S4 and Table S1, respectively).

### 2.4 RNA‐Seq

#### 2.4.1 RNA Extraction and Illumina Sequencing

Total RNA was extracted from one larva per sampled plant and date, using Spectrum Plant Total RNA Kit (Sigma‐Aldrich) according to the manufacturer's protocol. In pre‐experiments, we verified insect RNA quantity and integrity spectrophotometrically (NanoDrop 1000 Spectrophotometer, Thermo Scientific, Wilmington, USA) and by agarose gel electrophoresis (data not shown). DNA was removed during extraction using on‐column DNase I digestion (Sigma‐Aldrich). After extraction, larval RNA was pooled in equimolar amounts of 900 ng (3.7–32.1 μL) as follows. RNA from six individuals sampled from the same cultivar and VineyardFACE ring at the same date was pooled to obtain three replicates per cultivar, CO_2_ concentration and grapevine phenological stage. From these samples, ca. 4 μg of total RNA for each pool was ethanol precipitated and sent to Macrogen Korea (Seoul, Korea) for RNA sequencing. The total RNA was further processed, performing Ribo‐Zero rRNA removal, poly(A) + mRNA enrichment, and library preparation using the TruSeq Sample Prep approach. Libraries were sequenced on the Illumina platform using paired‐end (2 × 150 bp) read technology, resulting in 22–30 Mio reads per sample. Raw sequence data are available in the NCBI Sequence Read Archive under BioProject ID PRJNA910346 and can be assessed in the BioSample database under accession numbers SAMN32123559‐SAMN32123582 (Table S14).

#### 2.4.2 Transcriptome Assembly and Differential Gene Expression Analysis

Quality control measures and de novo transcriptome assembly combining all RNA‐Seq samples were carried out using CLC Genomics Workbench v12.0.2 (https://www.qiagen.com/qiagen‐clc‐genomics‐workbench/) as previously described (Jacobs et al. 2016). The de novo reference transcriptome assembly of *L. botrana* larvae contained 61,294 contigs (minimum contig size = 200 bp; minimum contig coverage cut‐off criteria = 3x) with an N50 contig length of 1025 bp and a maximum contig length of 29,149 bp. Transcriptome annotation using BLAST, Gene Ontology mapping and InterProScan implemented in OmicsBox (https://www.biobam.com/omicsbox/) was carried out as previously described (Jacobs et al. 2016). The quality and completeness of the assembly was assessed by BUSCO v.3 with the Arthropod BUSCO set from OrthoDB (database version 9.1). This resulted in 84.3% complete, 9.4% fragmented and 6.3% missing BUSCO genes for the *L. botrana* transcriptome assembly.

### 2.5 qPCR

Subsequent to RNA‐Seq analysis, expression of a subset of eight genes was validated via qPCR using the same RNA pools as for RNA‐Seq (primer sequences and details listed in Table S18). In addition, qPCR was conducted with RNA extracted from a separate batch of individual larvae which had fed on ‘Riesling’ inflorescences grown at ambient or elevated CO_2_ conditions. qPCR was conducted using RevertAid First Strand cDNA Synthesis Kit (Thermo Scientific) and Maxima SYBR Green (Thermo Scientific) on an iQ5 Multicolor iCycler (Bio‐Rad). Three technical replicates were run per sample for each gene. Normalised relative expression levels were calculated based on two *L. botrana* housekeeping genes (actin, EF1α; Table S18), which were evaluated based on expression stability (*M* values) and coefficients of variation (CV) using qbase+ Version 3.3 (Biogazelle). Analysis of gene expression levels was performed using the tool ‘Do my qPCR calculation’ (Tournayre et al. 2019). Further details on quality and quantity of the RNA‐Seq annotation and validation via qPCR can be found in Supporting Information S15–S17.

### 2.6 Data Analysis

Larval RGR and plant amino acid, sugar and phenolic compound concentrations were evaluated with RStudio (R version 4.1.1), using generalised linear models (GLM, Gamma family; R package *stats*) including three factors: either CO_2_, cultivar, plant phenology or CO_2_, cultivar and herbivory, and their interactions, followed by general linear hypotheses *post hoc* test (glht, Tukey; R package *multcomp*) for multiple comparisons with *p* < 0.05 considered significant. Transcriptome data and digital gene expression analysis were evaluated using CLC Genomics workbench v12.0.2 to generate BAM mapping files and by counting the sequences to estimate expression levels, employing previously described parameters (Jacobs et al. 2016). Mapped reads were normalised and log2 transformed using GLM‐based statistical methods with the ‘empirical analysis of digital gene expression’ (EDGE) tool, implemented in CLC Genomics Workbench, allowing for a non‐constant mean–variance relationship of the read count data. The thresholds for significantly differentially expressed genes were a minimum two‐fold change in expression and a false discovery rate (FDR)‐corrected *p*‐value of < 0.05. The qPCR data were evaluated in pairwise comparisons between *L. botrana* larvae which were feeding on grapevine cultivated under ambient or elevated CO_2_ conditions using a one‐tailed Student's t‐test with a *p*‐value of < 0.05 considered significant. VENN‐diagrams were created based on the EDGE analysis results using the web application JVENN (Bardou et al. 2014). Principal component analyses (PCA) were conducted with auto‐scaled data on the web‐based application MetaboAnalyst 5.0 (https://www.metaboanalyst.ca/; The Metabolomics Innovation Centre and McGill University, Montreal, Canada) (Xia and Wishart 2016).

## 3 Results

### 3.1 Biotic and Abiotic Impact on Host Plant Quality

**FIGURE 1:**
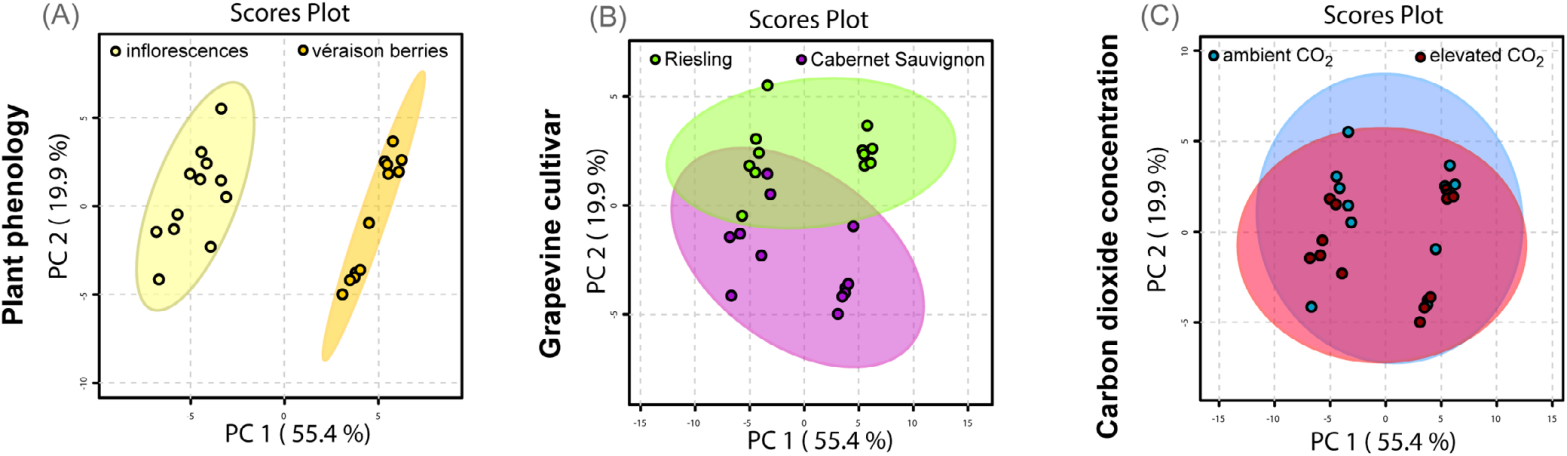
Principal component analyses of the biochemical plasticity of the reproductive organs of grapevine cultivated in the VineyardFACE, depending on plant phenology (A), grapevine cultivar (B), and atmospheric CO_2_ concentration (C). Loadings are listed in Table S5A–C. Ellipses indicate the respective 95%‐confidence intervals of the samples in the corresponding colours.

The biochemical variance of grapevine reproductive organs of two cultivars, at two different phenological stages as well as under ambient and elevated CO_2_ concentrations was analysed by PCA (Figure 1; for PCA loadings please see Table S7A–C). The plots indicate that the highest variance is related to plant phenology (Figure 1A), followed by grapevine cultivar (Figure 1B) whereas the CO_2_ concentration only manifests in minor variance (Figure 1C).

#### 3.1.1 Host Plant Nutritional Value

**FIGURE 2:**
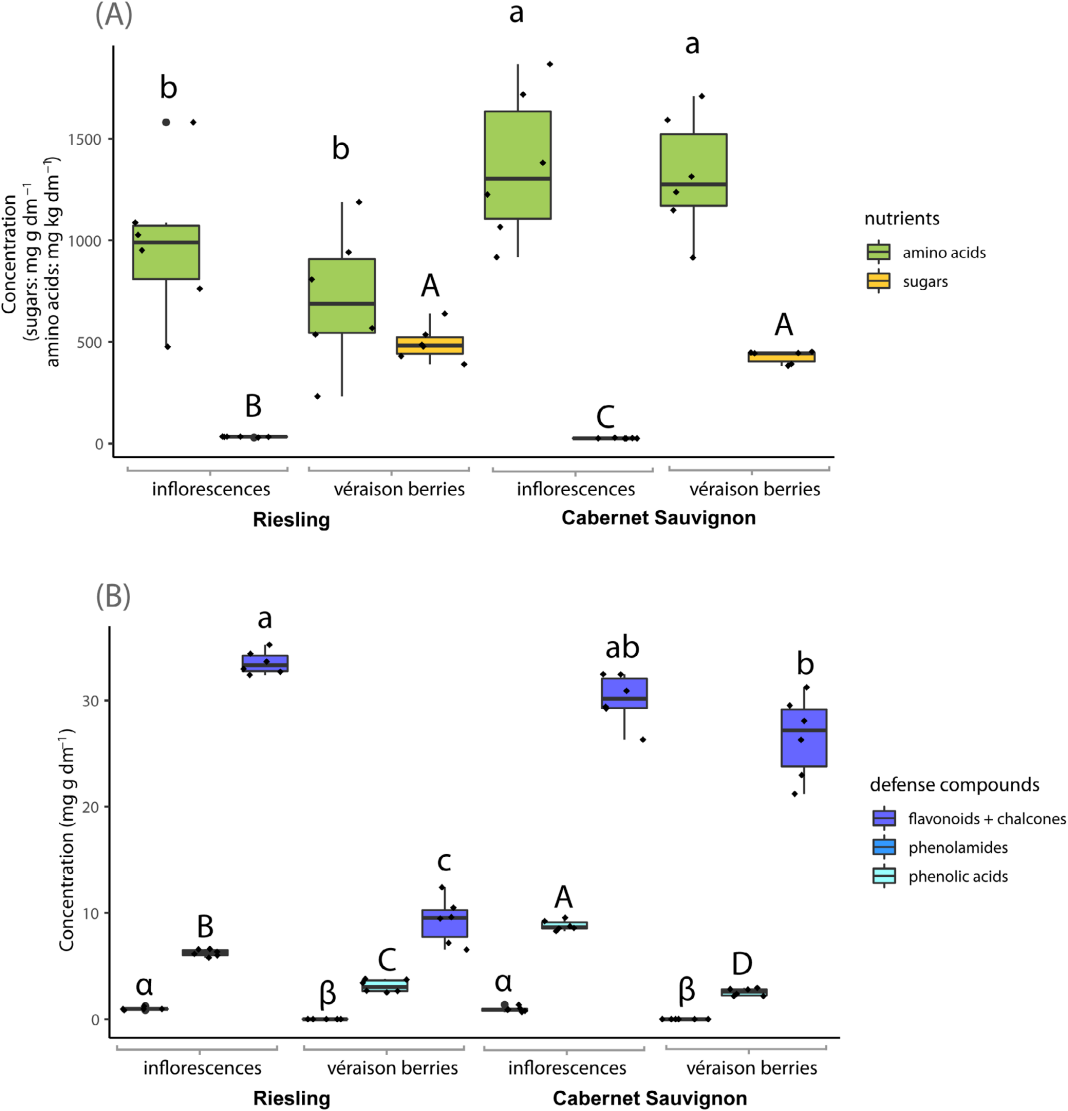
Concentrations of nutrients (A; green boxes: amino acids, yellow boxes: sugars) and defence compounds (B; dark blue: flavonoids + chalcones, light blue: phenolamids, cyan: phenolic acids) in grapevine reproductive organs as impacted by plant phenology and cultivar (GLM, *n* = 3; mg g^−1^ and kg^−1^ dry matter, respectively). Plants were cultivated in the VineyardFACE but the presented sums were not significantly impacted by CO_2_ concentration. Different upper case, lower case, and Greek letters, respectively, indicate significant differences between treatments.

**FIGURE 3:**
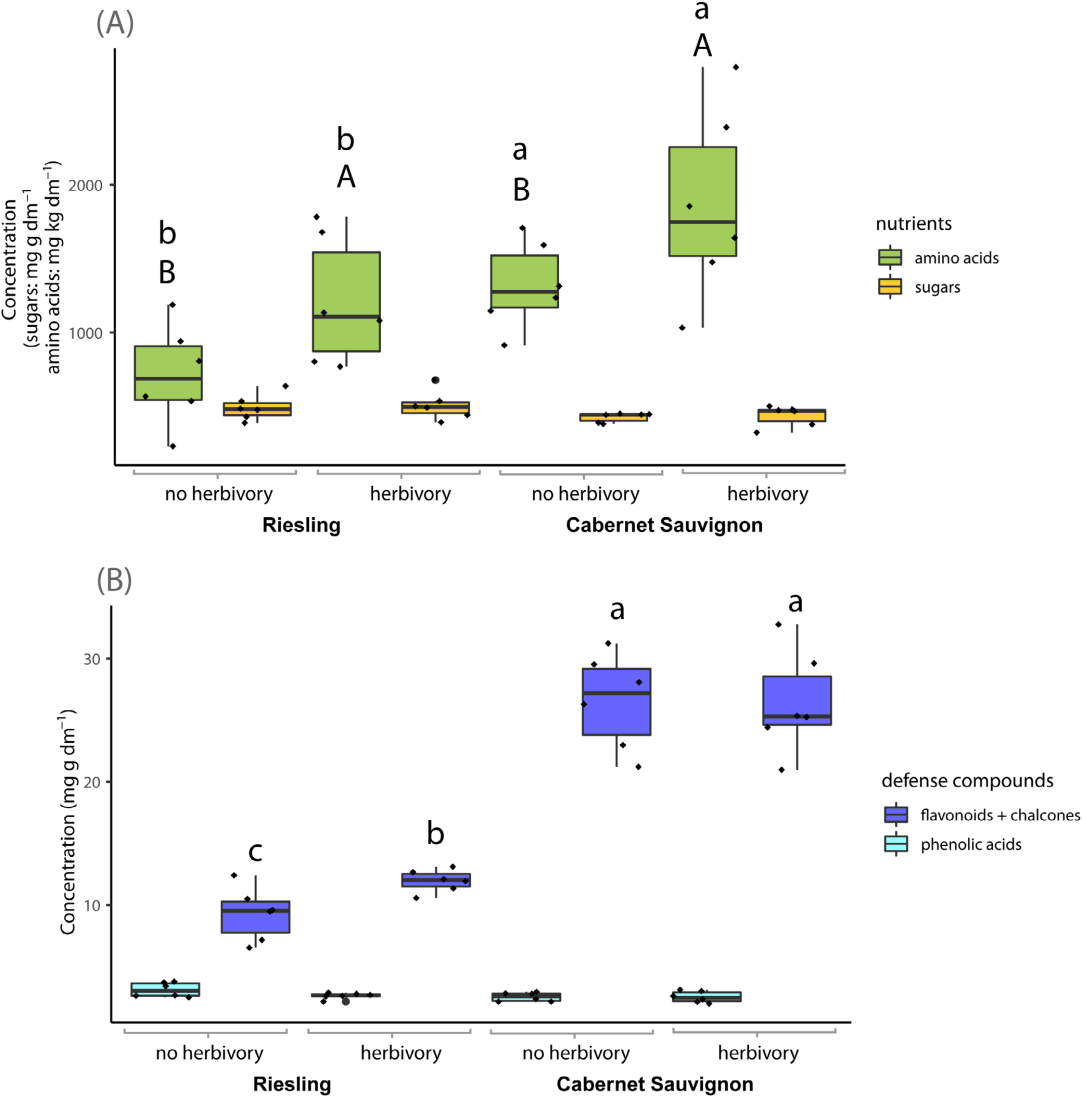
Concentrations of nutrients (A; green boxes: amino acids, yellow boxes: sugars) and defence compounds (B; dark blue: flavonoids + chalcones, cyan: phenolic acids) in grapevine reproductive organs as impacted by herbivory and cultivar (GLM, *n* = 3; mg g^−1^ and kg^−1^ dry matter, respectively). Plants were cultivated in the VineyardFACE but the presented sums were not significantly impacted by CO_2_ concentration. Different upper and lower case letters, respectively, indicate significant differences between treatments.

**FIGURE 4:**
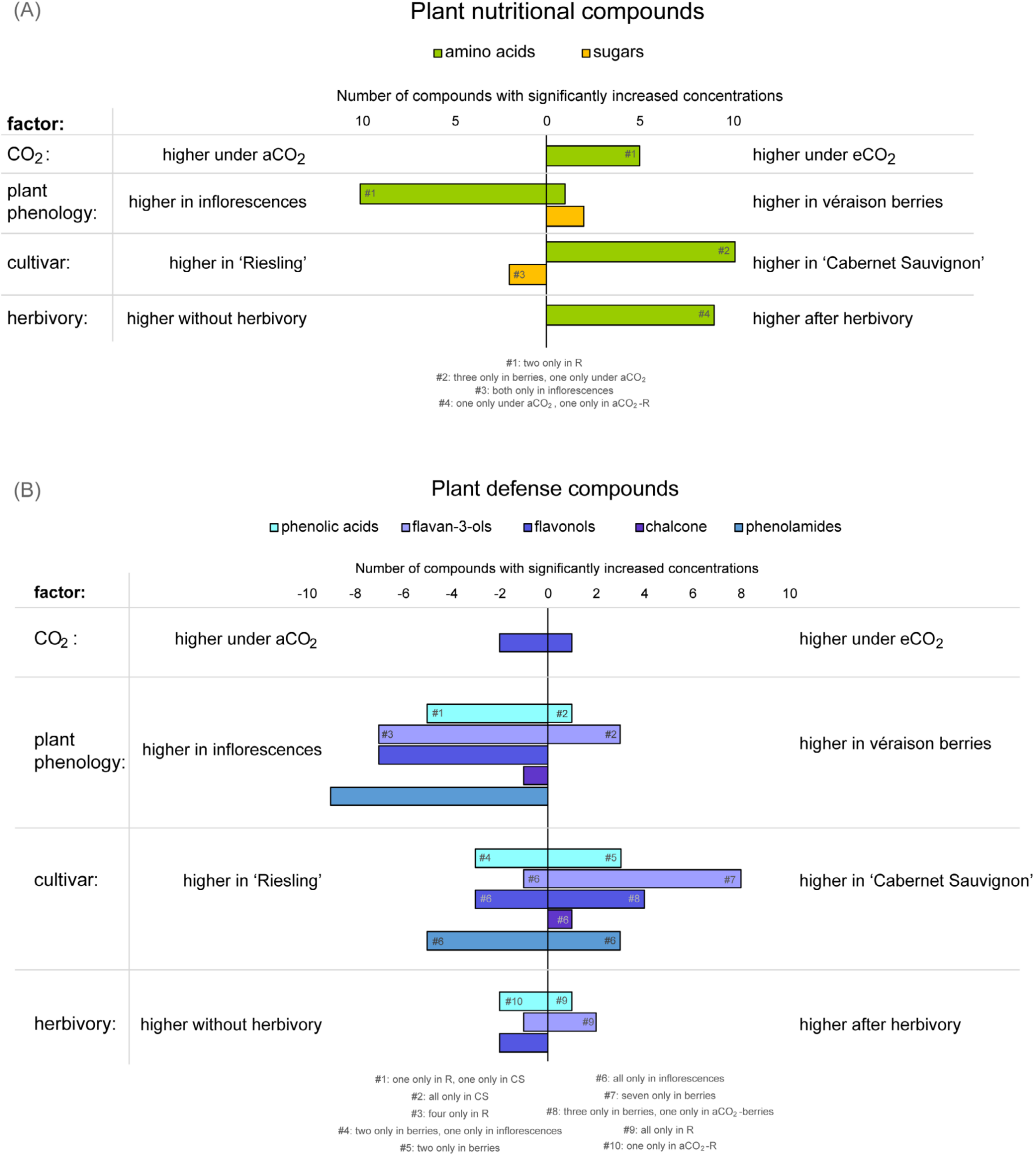
Number of (A) plant nutritional and (B) defence compounds with significantly higher concentrations in grapevine submitted to different treatments. The respective plant reproductive organs (inflorescences or véraison berries of the cultivar ‘Riesling’ or ‘Cabernet Sauvignon’) were cultivated under different CO_2_ concentrations (aCO_2_: ambient and eCO_2_: elevated) in the Geisenheim VineyardFACE. Regarding véraison berries, the impact of 7 days of *L. botrana* larval feeding was studied additionally.

In total, ‘Cabernet Sauvignon’ contained significantly higher concentrations of amino acids than ‘Riesling’ (cultivar: *F*_1,23_ = 9.9, *p* = 0.006; Table 1; Figure 2A). The concentration of sugars was significantly higher in véraison berries than in inflorescences (plant phenology: *F*_1,23_ = 11.1, *p* = 0.004; Table 1; Figure 2A). The concentration of amino acids in véraison berries that larvae had fed on was significantly higher (herbivory: *F*_1,23_ = 6.5, *p* = 0.02; Table 1; Figure 3A) whereas the concentration of sugars did not differ (herbivory: *F*_1,23_ = 0.2, *p* = 0.70; Figure 3A). Detailed changes in sugar and amino acid concentrations are shown in Figure 4 and Table S6 and described in the following paragraphs, with the respective concentrations and detailed statistical evaluations listed in Tables S8, S9, and S12A,B.

**TABLE 1:** Details of the statistical evaluation of the concentrations of amino acids and sugars in grapevine reproductive organs as influenced by CO_2_ concentration (ambient: 409 ppm, elevated: 485 ppm), cultivar (‘Riesling’ and ‘Cabernet Sauvignon’), and plant phenological stage (inflorescences and véraison) or *L. botrana* larval herbivory (GLM, family = Gamma, link = inverse).

Compound	Factor	Df	Deviance	Resid. Df	Resid. Dev	*F*	*p*‐value
Amino acids (sum)	NULL			23	4.38		
	CO_2_	1	0.34	22	4.04	2.71	0.1192
	Plant_phenology	1	0.12	21	3.92	0.96	0.3408
	**Cultivar**	1	1.24	20	2.68	9.89	**0.0063**
	CO_2_*plant_phenology	1	0.01	19	2.68	0.05	0.8336
	CO_2_*cultivar	1	0.37	18	2.31	2.97	0.1041
	Plant_phenology*cultivar	1	0.17	17	2.14	1.37	0.2591
	CO_2_*plant_phenology*cultivar	1	0.00	16	2.14	0.01	0.9252
	NULL			23	5.63		
	**Herbivory**	1	1.02	22	4.61	6.51	**0.0214**
	CO_2_	1	0.13	21	4.48	0.83	0.3753
	**Cultivar**	1	1.45	20	3.02	9.24	**0.0078**
	Herbivory*CO_2_	1	0.02	19	3.00	0.14	0.7126
	Herbivory*cultivar	1	0.22	18	2.78	1.39	0.2556
	CO_2_*cultivar	1	0.07	17	2.71	0.47	0.5021
	Herbivory*CO_2_*cultivar	1	0.09	16	2.62	0.57	0.4630
Sugars (sum)	NULL			23	36.02		
	CO_2_	1	0.00	22	36.02	0.03	0.8590
	**Plant_phenology**	1	35.59	21	0.43	2671.71	**< 0.0001**
	**Cultivar**	1	0.08	20	0.36	5.64	**0.0304**
	CO_2_*plant_phenology	1	0.00	19	0.36	0.07	0.7960
	CO_2_*cultivar	1	0.00	18	0.35	0.06	0.8100
	Plant_phenology*cultivar	1	0.15	17	0.21	11.11	**0.0042**
	CO_2_*plant_phenology*cultivar	1	0.00	16	0.20	0.15	0.7024
	NULL			23	0.63		
	Herbivory	1	0.00	22	0.62	0.15	0.6995
	CO_2_	1	0.01	21	0.62	0.18	0.6810
	Cultivar	1	0.13	20	0.49	4.41	0.0518
	Herbivory*CO_2_	1	0.01	19	0.48	0.30	0.5899
	Herbivory*cultivar	1	0.00	18	0.48	0.00	0.9952
	CO_2_*cultivar	1	0.00	17	0.48	0.04	0.8487
	Herbivory*CO_2_*cultivar	1	0.01	16	0.47	0.20	0.6599
Phenolic acids (sum)	NULL			23	6.29		
	CO_2_	1	0.00	22	6.29	0.00	0.9924
	**Plant_phenology**	1	5.51	21	0.78	367.61	**< 0.0001**
	**Cultivar**	1	0.19	20	0.59	12.53	**0.0027**
	CO_2_*plant_phenology	1	0.01	19	0.58	0.69	0.4186
	CO_2_*cultivar	1	0.01	18	0.58	0.34	0.5662
	Plant_phenology*cultivar	1	0.29	17	0.29	19.58	**0.0004**
	CO_2_*plant_phenology*cultivar	1	0.04	16	0.25	2.55	0.1295
	NULL			23	0.66		
	Herbivory	1	0.05	22	0.61	2.01	0.1759
	CO_2_	1	0.01	21	0.60	0.28	0.6008
	Cultivar	1	0.09	20	0.51	3.44	0.0820
	Herbivory*CO_2_	1	0.00	19	0.51	0.09	0.7697
	Herbivory*cultivar	1	0.04	18	0.47	1.50	0.2379
	CO_2_*cultivar	1	0.00	17	0.47	0.14	0.7088
	Herbivory*CO_2_*cultivar	1	0.05	16	0.41	2.13	0.1641
Flavonoids (sum)	NULL			23	5.59		
	CO_2_	1	0.01	22	5.59	0.24	0.6331
	**Plant_phenology**	1	1.96	21	3.63	86.19	**< 0.0001**
	**Cultivar**	1	0.44	20	3.19	19.16	**0.0005**
	CO_2_*plant_phenology	1	0.00	19	3.19	0.01	0.9349
	CO_2_*cultivar	1	0.00	18	3.19	0.15	0.6992
	Plant_phenology*cultivar	1	2.77	17	0.42	122.15	**< 0.0001**
	CO_2_*plant_phenology*cultivar	1	0.02	16	0.40	0.82	0.3795
	NULL			23	5.58		
	Herbivory	1	0.03	22	5.55	0.92	0.3520
	CO_2_	1	0.00	21	5.55	0.01	0.9045
	**Cultivar**	1	4.84	20	0.71	161.28	**< 0.0001**
	Herbivory*CO_2_	1	0.00	19	0.71	0.00	0.9493
	Herbivory*cultivar	1	0.17	18	0.54	5.62	0.0307
	CO_2_*cultivar	1	0.00	17	0.54	0.02	0.8788
	Herbivory*CO_2_*cultivar	1	0.03	16	0.51	1.07	0.3160
Phenolamides (sum)	NULL			23	187.25		
	CO_2_	1	0.00	22	187.24	0.17	0.6896
	**Plant_phenology**	1	186.86	21	0.38	10167.82	**< 0.0001**
	Cultivar	1	0.01	20	0.37	0.41	0.5302
	CO_2_*plant_phenology	1	0.00	19	0.37	0.00	1.0000
	CO_2_*cultivar	1	0.07	18	0.31	3.61	0.0756
	Plant_phenology*cultivar	1	0.00	17	0.31	0.00	0.9999
	CO_2_*plant_phenology*cultivar	1	0.00	16	0.31	0.00	0.9998

*Note:* Values in bold font indicate significant differences between treatments (*p* < 0.05).

##### 3.1.1.1 Impact of CO_2_

Grapevine reproductive organs contained significantly higher concentrations of five amino acids (isoleucine, lysine, glycine, tyrosine and methionine; only the first three significant in ‘Cabernet Sauvignon’; Figure 4A; Table S12A,B) when cultivated under elevated compared with ambient CO_2_. Regarding sugar concentration, no significant differences because of CO_2_ concentration were detected.

##### 3.1.1.2 Impact of Plant Phenology and Cultivar

Inflorescences contained significantly higher concentrations of 10 amino acids (isoleucine, lysine, methionine, valine, tryptophan, alanine, serine, glycine, histidine and proline; only the first eight significant in ‘Cabernet Sauvignon’) and significantly lower concentrations of arginine compared with véraison berries (Figure 4A; Table S12A,B). ‘Cabernet Sauvignon’ contained significantly higher concentrations of six amino acids (isoleucine, valine, tyrosine, tryptophan, arginine and glycine) than ‘Riesling’ and, additionally, concentrations of three amino acids were significantly higher in ‘Cabernet Sauvignon’ berries than ‘Riesling’ berries (phenylalanine, histidine and proline) and of one in aCO_2_‐‘Cabernet Sauvignon’ compared with aCO_2_‐‘Riesling’ (methionine; Figure 4A; Table S12A,B). Glucose and fructose concentrations were significantly higher in véraison berries than in inflorescences and in ‘Riesling’ inflorescences compared with ‘Cabernet Sauvignon’ inflorescences (Figure 4A; Table S12A,B). Sucrose concentration could only be measured in inflorescence samples, as the ratio between glucose and sucrose was too high in the véraison berries for the enzymatic kit used.

##### 3.1.1.3 Herbivory

Véraison berries that larvae had fed on contained significantly higher concentrations of a total of nine amino acids, seven in both cultivars (valine, lysine, tryptophan, arginine, proline, alanine and serine), one in ‘Riesling’ with signs of herbivory (histidine) and one in aCO_2_‐‘Riesling’ with signs of herbivory (methionine; Figure 4A; Table S12A,B). No significant differences in sugar concentrations between berries with and without signs of larval feeding were detected.

#### 3.1.2 Host Plant Defence Compounds

The concentrations of all 33 detected phenolic compounds were significantly affected by at least one of the tested treatments (Figure 4B). In total, inflorescences contained significantly higher concentrations of phenolamides (plant phenology: *F*_1,23_ = 10167.8, *p* < 0.0001; Table 1; Figure 2B), phenolic acids (plant phenology*cultivar: *F*_1,23_ = 19.6, *p* = 0.0004; Table 1; Figure 2B), and flavonoids and chalcones (only significant in ‘Riesling’; plant phenology*cultivar: *F*_1,23_ = 122.2, *p* < 0.0001; Table 1; Figure 2B) than véraison berries. ‘Riesling’ that larvae had fed on contained significantly higher concentrations of flavonoids (herbivory*cultivar: *F*_1,23_ = 5.6, *p* = 0.03; Table 1; Figure 3B) whereas the phenolic acid concentrations in both cultivars did not differ significantly (herbivory: *F*_1,23_ = 2.0, *p* = 0.60; Table 1; Figure 3B). Detailed changes of phenolic compound concentrations are shown in Figure 4 and Table S6, and are described in the following paragraphs, with the respective concentrations and detailed statistical evaluations listed in Tables S10, S11 and S13A,B.

##### 3.1.2.1 Impact of CO_2_

Grapevine reproductive organs cultivated under eCO_2_ compared with aCO_2_ contained significantly higher concentrations of one flavonol glycoside (quercetin 3‐*O*‐glucoside) but significantly lower concentrations of two others (kaempferol 3‐*O*‐glucoside, kaempferol 3‐*O*‐glucuronide; Figure 4B; Tables S6 and S13A,B).

##### 3.1.2.2 Impact of Plant Phenology and Cultivar

Compared with véraison berries, inflorescences contained significantly higher concentrations of 32 phenolic compounds (*p*‐coumaroyltartaric acid, feruloyltartaric acid, caffeoyltartaric acid, procyanidin B1, procyanidin B2, procyanidin C1, quercetin 3‐*O*‐glucoside, quercetin 3‐*O*‐glucuronide, quercetin 3‐*O*‐galactoside, quercetin 3‐*O*‐rutinoside, quercetin 3‐*O*‐(6″‐malonyl)hexoside, kaempferol 3‐*O*‐rutinoside, kaempferol 3‐*O*‐galactoside, kaempferol 3‐*O*‐glucoside, kaempferol 3‐*O*‐glucuronide, naringenin chalcone, *N* ^1^,*N* ^5^,*N* ^10^‐tri‐caffeoylspermidine, *N*,*N*′,*N*″‐di‐caffeoyl‐*p*‐coumaroylspermidine (2 isomers), *N*,*N*′,*N*″‐di‐caffeoyl‐feruloylspermidine, *N*,*N*′,*N*″‐caffeoyl‐di‐*p*‐coumaroylspermidine (2 isomers), *N*,*N*′,*N*″‐caffeoyl‐*p*‐coumaroyl‐feruloylspermidine, *N* ^1^,*N* ^5^,*N* ^10^‐tri‐*p*‐coumaroylspermidine, *N*,*N*′,*N*″‐di‐*p*‐coumaroyl‐feruloylspermidine, as well as a galloyl hexose, a procyanidin dimer gallate, (+)‐catechin, (−)‐epicatechin, an (epi)catechin gallate only in ‘Riesling’ and *p*‐coumaric acid hexose + *cis*‐caffeoyltartaric acid only in ‘Cabernet Sauvignon’) whereas, compared with inflorescences, ‘Cabernet Sauvignon’ véraison berries contained significantly higher concentrations of only four phenolic compounds (galloyl hexose, (+)‐catechin, (−)‐epicatechin and (epi)catechin gallate; Figure 4B; Tables S6 and S13A,B).

Compared with ‘Riesling’, ‘Cabernet Sauvignon’ contained significantly higher concentrations of 2 phenolic compounds (*p*‐coumaroyltartaric acid, (epi)gallocatechin) (Figure 4B; Tables S6 and S13A,B). The impact of grapevine cultivar interacted with plant phenology regarding the following compounds. In inflorescences, the concentrations of 13 phenolic compounds were significantly higher in ‘Riesling’ than in ‘Cabernet Sauvignon’ (*p*‐coumaric acid hexose + *cis*‐caffeoyltartaric acid, procyanidin B2, quercetin 3‐*O*‐(6″‐malonyl)hexoside, kaempferol 3‐*O*‐rutinoside, kaempferol 3‐*O*‐galactoside, kaempferol 3‐*O*‐glucoside, kaempferol 3‐*O*‐glucuronide, *N*,*N*′,*N*″‐di‐caffeoyl‐*p*‐coumaroylspermidine (isomer 1 and 2), *N*,*N*′,*N*″‐caffeoyl‐di‐*p*‐coumaroylspermidine (isomer 1 and 2), *N* ^1^,*N* ^5^,*N* ^10^‐tri‐*p*‐coumaroylspermidine, whereas the concentrations of four phenolic compounds was significantly higher in ‘Cabernet Sauvignon’ than in ‘Riesling’ (naringenin chalcone, *N*,*N*′,*N*″‐di‐caffeoyl‐feruloylspermidine, *N*,*N*′,*N*″‐caffeoyl‐*p*‐coumaroyl‐feruloylspermidine and *N*,*N*′,*N*″‐di‐*p*‐coumaroyl‐feruloylspermidine; Figure 4B; Tables S6 and S13A,B). In véraison berries, the concentrations of two phenolic compounds were significantly higher in ‘Riesling’ than in ‘Cabernet Sauvignon’ (caffeoyl‐ and feruloyltartaric acid) whereas the concentrations of 14 phenolic compounds were significantly higher in ‘Cabernet Sauvignon’ than in ‘Riesling’ (galloyl hexose, *p*‐coumaric acid hexose + *cis*‐caffeoyl tartaric acid, procyanidin B1, procyanidin B2, procyanidin C1, procyanidin dimer gallate, (+)‐catechin, (−)‐epicatechin, (epi)catechin gallate, quercetin 3‐*O*‐glucoside, quercetin 3‐*O*‐glucuronide, quercetin 3‐*O*‐galactoside as well as quercetin 3‐*O*‐rutinoside only in eCO_2_‐véraison berries; Figure 4B; Tables S6 and S13A,B).

##### 3.1.2.3 Herbivory

Véraison berries that larvae had fed on contained significantly lower concentrations of six phenolic compounds (caffeoyl tartaric acid, (epi)gallocatechin, quercetin 3‐*O*‐glucoside, quercetin 3‐*O*‐glucuronide as well as *p*‐coumaric acid hexose + *cis*‐caffeoyl tartaric acid only in aCO_2_‐‘Riesling’). The concentration of three additional phenolic compounds was significantly higher in ‘Riesling’ berries that larvae had fed on (galloylhexose, procyanidin B1 and procyanidin B2; Figure 4B; Tables S6 and S13A,B).

### 3.2 Biotic and Abiotic Impacts on Herbivore Performance and Transcriptomics

#### 3.2.1 Larval Relative Growth Rate

**FIGURE 5:**
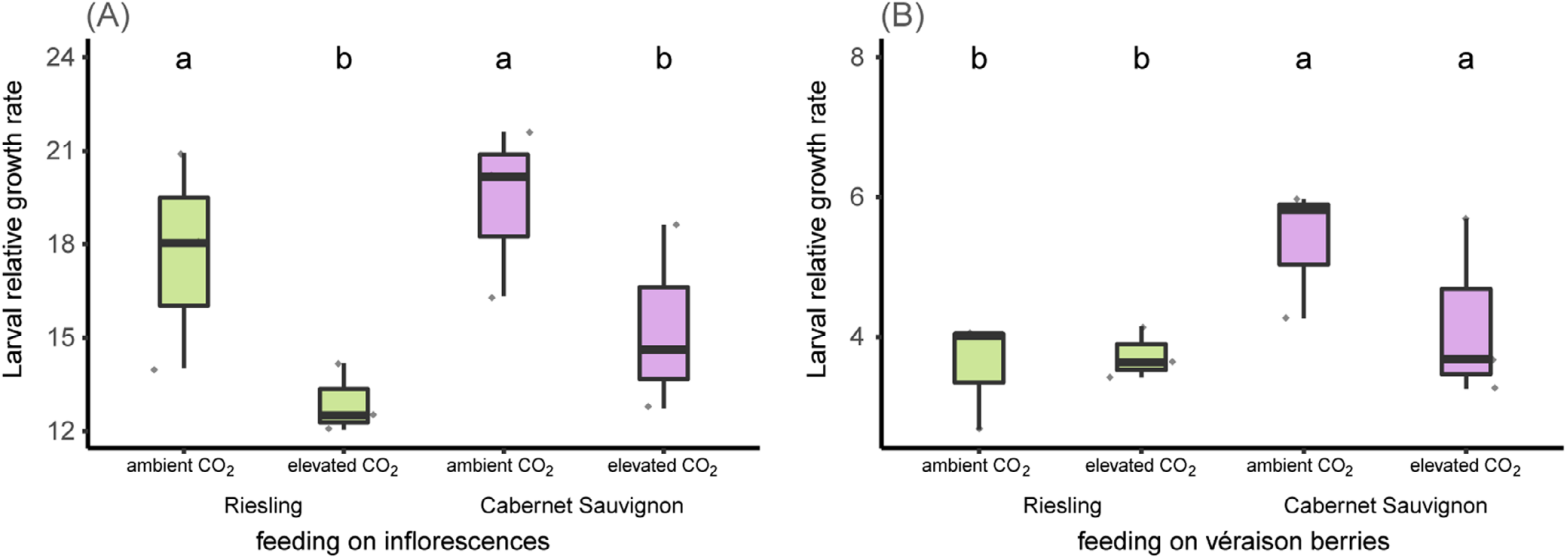
Relative growth rate of *Lobesia botrana* larvae feeding for 7 days on grapevine in different phenological stages (A: Inflorescences and B: Véraison berries) of two cultivars (green boxes: ‘Riesling’ and purple boxes: ‘Cabernet Sauvignon’) grown at elevated versus ambient CO_2_ concentration in the VineyardFACE (GLM, *n* = 3; data were pooled from six larvae feeding on six plants per FACE ring).

When feeding on grapevine inflorescences, larval RGR was affected by atmospheric CO_2_ concentration (significantly higher when feeding on aCO_2_‐ than on eCO_2_‐grapevine inflorescences; CO_2_: *χ* ^2^_10_ = 0.22, *p* = 0.004, cultivar: χ^2^_9_ = 0.05, *p* = 0.18; CO_2_*cultivar: *χ* ^2^_8_ = 0.01, *p* = 0.54; Figure 5A). When feeding on véraison berries, larval RGR was affected by grapevine cultivar (significantly higher when feeding on ‘Cabernet Sauvignon’ than on ‘Riesling’; CO_2_: *χ* ^2^_10_ = 0.04, *p* = 0.34, cultivar: *χ* ^2^_9_ = 0.21, *p* = 0.03; CO_2_*cultivar: *χ* ^2^_8_ = 0.04, *p* = 0.37; Figure 5B). Overall, the RGR was higher when larvae were feeding on inflorescences than on véraison berries.

#### 3.2.2 Larval Transcriptomic Response

**FIGURE 6:**
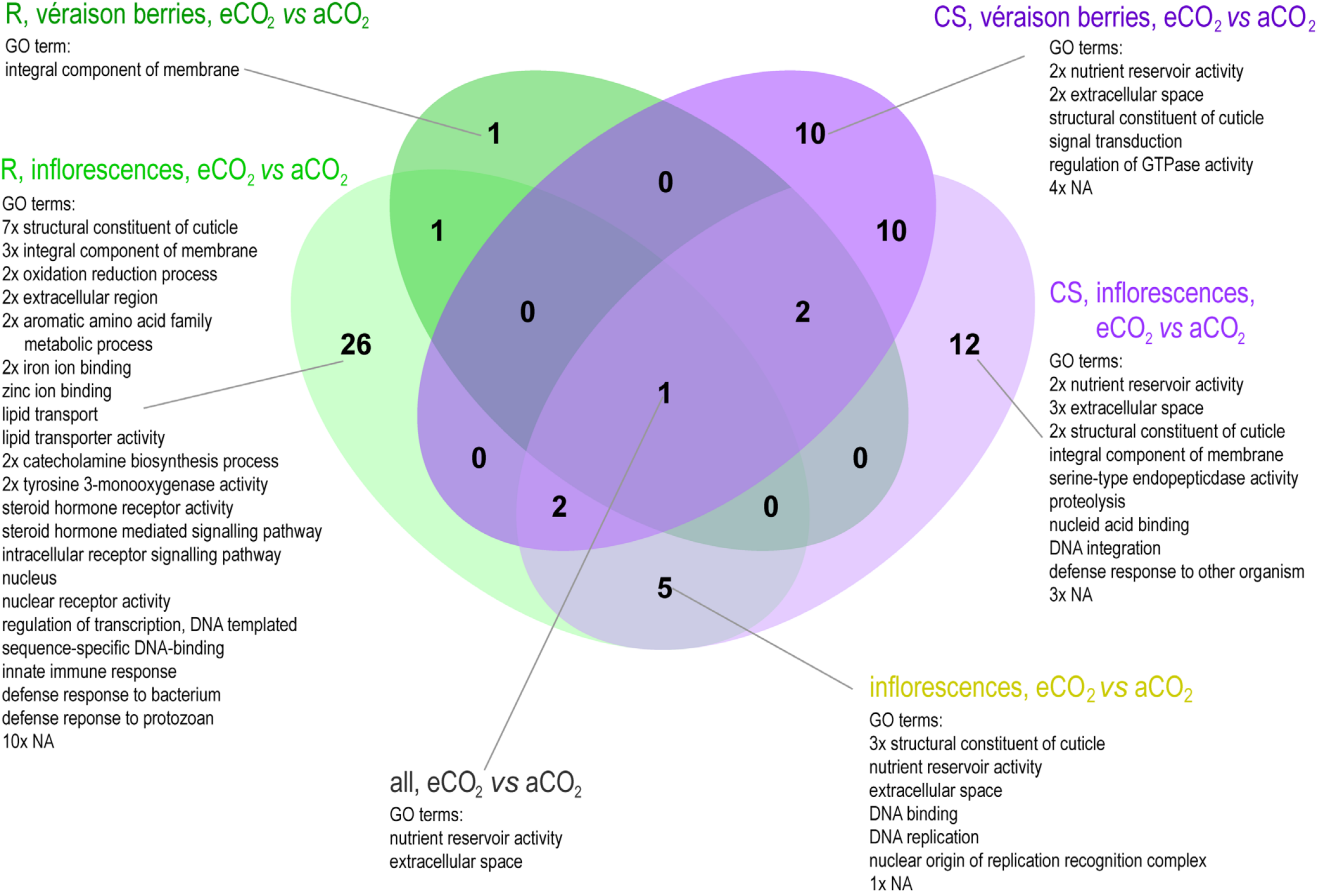
VENN diagram showing the number of significantly differentially expressed contigs in the *Lobesia botrana* larval transcriptome, after feeding on grapevine cultivars ‘Riesling’ (R) or ‘Cabernet Sauvignon’ (CS), inflorescences or véraison berries, grown under elevated (e) or ambient (a) CO_2_ concentration in the VineyardFACE and the respective gene ontology (GO) terms.

The VENN diagram (Figure 6) revealed only one significantly differentially expressed contig shared by larvae that have fed on both cultivars, plant developmental stages and CO_2_ concentrations. This contig was annotated as arylphorin precursor (GO:0005615, extracellular space; GO:0045735 nutrient reservoir activity). The majority of significantly differentially expressed contigs was associated with larval tissue (cuticle and membranes, GO:0042302; structural constituents of the cuticle, GO:0016021; integral components of membranes; Figure 6) and nutrient metabolism (GO: 0045735, nutrient reservoir activity; and others; Figure 6).

##### 3.2.2.1 Impact of CO_2_

**FIGURE 7:**
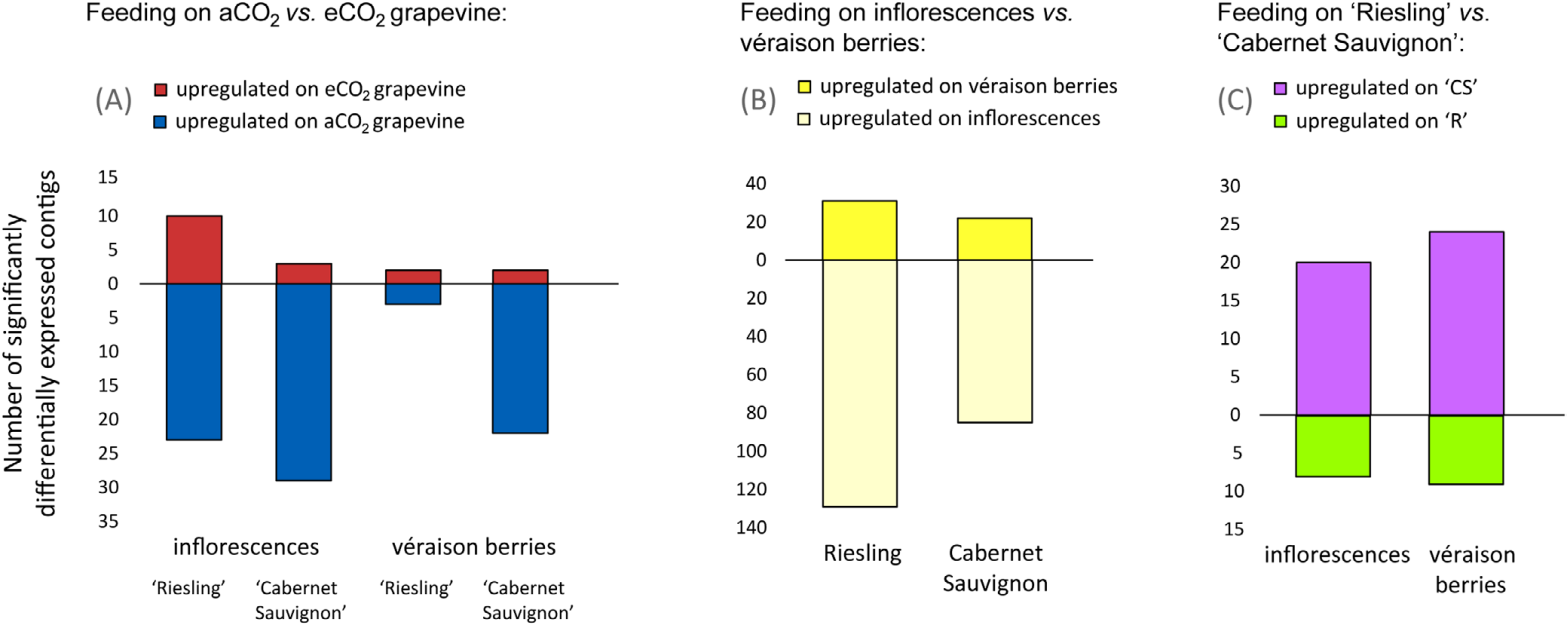
Number of contigs that were significantly differentially expressed in *Lobesia botrana* larvae after feeding for 7 days on grapevine reproductive organs (A) cultivated under two different CO_2_ concentrations (aCO_2_: Ambient; eCO_2_: Elevated), (B) at two different plant phenological stages (inflorescences, véraison berries) and (C) of two different cultivars (R: ‘Riesling’ and CS: ‘Cabernet Sauvignon’).

When larvae were feeding on grapevine cultivated under ambient versus elevated CO_2_ concentration, 77 contigs were expressed at significantly higher levels on aCO_2_‐grapevine and 17 on eCO_2_‐grapevine (Figure 7A). When feeding on inflorescences, 65 contigs were significantly differentially expressed because of CO_2_ concentration and 29 when feeding on véraison berries (Figure 7A). When larvae were feeding on ‘Riesling’ véraison berries, five larval contigs were significantly differentially expressed because of CO_2_ concentration (Figure 7A).

##### 3.2.2.2 Impact of Plant Phenology and Cultivar

When larvae were feeding on grapevine inflorescences versus véraison berries, 214 contigs were expressed at significantly higher levels when feeding on inflorescences and only 51 on véraison berries (Figure 7B). When feeding on ‘Riesling’, 160 larval contigs were significantly impacted by the plant's phenological stage. Similarly, when feeding on ‘Cabernet Sauvignon’ 105 contigs were affected (Figure 7B). When the larvae were feeding on ‘Cabernet Sauvignon’ compared to ‘Riesling’, 44 contigs were expressed at significantly higher levels, whereas only 17 were expressed on ‘Riesling’ (Figure 7C). The expression of 28 larval contigs was significantly affected by grapevine cultivar when feeding on inflorescences, and 33 contigs were affected on véraison berries (Figure 7C).

##### 3.2.2.3 Digestive Enzymes

Tables S19–S30 list all contigs that were significantly differentially expressed in larvae feeding on grapevine from the different treatments. We analysed these data with special regard to contigs assigned to enzymes contributing to digestion and detected changes in the transcript levels of peptide, lipid and carbohydrate metabolism‐related genes (Table 2).

**TABLE 2:** *Lobesia botrana* larval contigs assigned to digestive and detoxifying enzymes that showed significantly differential expression levels after feeding on grapevine inflorescences or véraison berries of the cultivars ‘Riesling’ (R) and ‘Cabernet Sauvignon’ (CS), cultivated at ambient (409 ppm) or elevated (485 ppm) CO_2_ concentration in the VineyardFACE (*p*‐value FDR‐corrected < 0.05; *n* = 3). For a complete list of significantly differentially expressed contigs per scenario please see Tables S19–S30.

Impact factor		Digestive enzymes		Detoxifying enzymes	
		Feeding on R (fold change)	Feeding on CS (fold change)	Feeding on R (fold change)	Feeding on CS (fold change)
CO_2_ (ambient vs. elevated)	Upregulated after feeding on elevated CO_2_‐grapevine	—	—	—	—
	Downregulated after feeding on elevated CO_2_‐grapevine	On inflorescences: 2 × tyrosine hydroxylase (−5.7, −5.5)	On inflorescences: 1 × trypsin CFT‐1‐like (−10.7)	—	—
Plant phenology (inflorescences vs. véraison berries)	Upregulated after feeding on véraison berries	On aCO_2_‐grapevine: 1 × fatty acid synthase (2.1) On eCO_2_‐grapevine: 2 × fatty acid synthase (3.2, 2.7)	On aCO_2_‐grapevine: 1 × fatty acid synthase (5.7) on eCO_2_‐grapevine: 1 × fatty acid synthase (3.7)	On aCO_2_‐grapevine: 1 × cytochrome P450 (2.2) On eCO_2_‐grapevine: 1 × cytochrome P450 (1.8)	On aCO_2_‐grapevine: 1 × cytochrome P450 (2.1) on eCO_2_‐grapevine: 1 × cytochrome P450 (2.4)
	Downregulated after feeding on véraison berries	On aCO_2_‐grapevine: 6 × pancreatic triacylglycerol lipase‐like (−6.6, −6.0, −3.8, −3.5, −2.2, −2.0) 2 × tyrosine hydroxylase (−16.7, −14.6) 1 × carboxypeptidase B‐like (−2.5) 1 × trypsin CFT‐1‐like (−1.8) 1 × sucrose‐6‐phosphate hydroxylase‐like (−3.7) On eCO_2_‐grapevine: 6 × pancreatic triacylglycerol lipase‐like (−8.8, −3.8, −3.3, −2.4, −2.0, −2.0) 2 × carboxypeptidase B‐like (−4.1, −1,7) 1 × trypsin CFT‐1‐like (−2.3) 1 × zinc metalloproteinase nas4‐like (−1.9) 1 × α‐amylase like (−2.7)	On aCO_2_‐grapevine: 8 × pancreatic triacylglycerol lipase‐like (−13.8, −8.8, −6.1, −5.9, −2.2, −2.2, −2.1, −1.8) 1 × trypsin CFT‐1‐like (−23.1) 2 × tyrosine hydroxylase (−7.4, −6.7) 1 × carboxypeptidase B‐like (−4.2) 1 × zinc metalloproteinase nas‐4‐like (−2.0) 1 × sucrose‐6‐phosphate hydrolase‐like (−3.3) 1 × α‐amylase 1‐like (−2.9) 1 × maltase A1‐like (−5.2) On eCO_2_‐grapevine: 4 × pancreatic triacylglycerol lipase‐like (−11.4, −6.6, −5.9, −2.2) 1 × carboxypeptidase B‐like (−4.7) 1 × trypsin CFT‐1‐like (−2.1) 1 × α‐amylase like (−3.0) 1 × maltase A1‐like (−3.1) 1 × sucrose‐6‐phosphate hydrolase‐like (−3.8)	On aCO_2_‐grapevine: ‐ On eCO_2_‐grapevine: 1 × cytochrome P450‐like (−4.2) 1 × gluthathione‐S‐transferase‐like (−3.0)	On aCO_2_‐grapevine: 1 × cytochrome P450‐like (−5.0) 1 × gluthathione‐S‐transferase‐like (−3.0) On eCO_2_‐grapevine: 2 × cytochrome P450‐like (−7.0, −4.5) 1 × gluthathione‐S‐transferase‐like (−3.2)
Cultivar (R vs. CS)	Upregulated when feeding on CS	—	—	—	—
	Downregulated when feeding on CS	—	—	—	—

###### 3.2.2.3.1 Impact of CO_2_

Compared to aCO_2_, when feeding on eCO_2_ ‘Riesling’ inflorescences, the expression of two putative larval genes associated with peptide metabolism was significantly downregulated (Table 2; Table S19). Similarly, when feeding on eCO_2_ ‘Cabernet Sauvignon’ inflorescences, the expression of one putative larval gene associated with peptide metabolism was significantly downregulated (Table 2; Table S21). No differentially expressed digestive enzyme‐annotated contigs were detected when feeding on véraison berries of either cultivar, regardless of CO_2_ concentration (Tables S20 and S22).

###### 3.2.2.3.2 Impact of Plant Phenology and Cultivar

When feeding on aCO_2_‐‘Riesling’ véraison berries compared with inflorescences, the expression of one putative larval gene associated with lipid metabolism was significantly upregulated whereas six putative larval genes associated with lipid metabolism were significantly downregulated as well as four putative larval genes associated with peptide metabolism and one with carbohydrate metabolism (Table 2; Table S23). When feeding on eCO_2_‐‘Riesling’ véraison berries compared with inflorescences, the expression of two putative larval genes associated with lipid metabolism was significantly upregulated, whereas six putative larval genes associated with lipid metabolism were significantly downregulated, along with four putative larval genes associated with peptide metabolism and one with carbohydrate metabolism (Table 2; Table S24). After the feeding process on aCO_2_‐‘Cabernet Sauvignon’ véraison berries, compared with inflorescences, the expression of one putative larval gene associated with lipid metabolism was significantly upregulated. In contrast, eight putative larval genes associated with lipid metabolism, five putative larval genes associated with peptide metabolism and three with carbohydrate metabolism were significantly downregulated (Table 2; Table S25). When feeding on eCO_2_‐‘Cabernet Sauvignon’ véraison berries compared with inflorescences, the expression of one putative larval gene associated with lipid metabolism was significantly upregulated. Additionally, four putative larval genes associated with lipid metabolism were significantly downregulated along with two putative larval genes associated with peptide metabolism and three with carbohydrate metabolism (Table 2; Table S26). Among the significantly differentially expressed larval contigs because of host plant cultivar, none were annotated as digestive enzymes (Tables S27–S30).

##### 3.2.2.4 Detoxifying Enzymes

We analysed the abovementioned data from Tables S19–S30 with special regard to enzymes involved in detoxification and detected some changes in the transcript levels of phase I and phase II enzymes but none regarding phase III transporters (Heidel‐Fischer and Vogel 2015).

###### 3.2.2.4.1 Impact of CO_2_

Among the significantly differentially expressed larval contigs because of CO_2_ concentration, none were annotated as detoxifying enzymes (Tables S19–S22).

###### 3.2.2.4.2 Impact of Plant Phenology and Cultivar

When feeding on aCO_2_‐‘Riesling’ véraison berries compared with inflorescences, the expression of one putative larval gene associated with detoxification was significantly upregulated (Table 2; Table S23). When feeding on eCO_2_‐‘Riesling’ véraison berries compared with inflorescences, the expression of one putative larval gene associated with detoxification was significantly upregulated whereas two others were significantly downregulated (Table 2; Table S24). When feeding on aCO_2_‐‘Cabernet Sauvignon’ véraison berries compared with inflorescences, the expression of one putative larval gene associated with detoxification was significantly upregulated whereas two others were significantly downregulated (Table 2, Table S25). When feeding on eCO_2_‐‘Cabernet Sauvignon’ véraison berries compared with inflorescences, the expression of one putative larval gene associated with detoxification was significantly upregulated whereas three others were downregulated (Table 2, Table S26). Among the significantly differentially expressed larval contigs due to host plant cultivar, none were annotated as detoxifying enzymes (Tables S27–S30).

##### 3.2.2.5 Defence and Reproduction.

###### 3.2.2.5.1 Impact of CO_2_

One putative defence protein‐contig (putative defence protein Hdd11, fold change: −4.8) and one related to sexual reproduction (ejaculatory bulb‐specific protein 3‐like isoform X2, fold change: −3.7) were significantly downregulated when larvae were feeding on eCO_2_‐ compared with aCO_2_‐‘Riesling’ inflorescences (Table S19).

###### 3.2.2.5.2 Impact of Plant Phenology and Cultivar

One putative defence protein‐contig (putative defence protein Hdd11) and one related to sexual reproduction (ejaculatory bulb‐specific protein 3‐like isoform X2) were significantly downregulated when larvae were feeding on aCO_2_‐‘Riesling’ véraison berries compared to inflorescences (fold change: −8.8, −4.5; Table S23).

## 4 Discussion

Here, we report on the biochemical variance of grapevine reproductive organs of two cultivars over the course of progressing phenology and under future elevated atmospheric CO_2_ concentration and describe the resulting bottom‐up effects on the plant's interaction with one of its major insect herbivores. Overall, grapevine biochemical variance was highest because of advancing plant phenology, followed by cultivar‐related differences and only then by elevated CO_2_ concentrations. This confirms our hypothesis 1. The biochemical profile of véraison berries with signs of herbivory did differ significantly from those without, supporting our hypothesis 2. The comparative analysis of gene expression patterns showed that insect transcriptomic plasticity was also highest because of advancing host plant phenology, followed by cultivar‐related differences and only then by elevated CO_2_ concentrations. Hence, it largely mirrored the host plant biochemical variance, confirming our hypothesis 3.

### 4.1 Plant Quality

We detected significant differences in sugar, amino acid and phenolic compound concentrations in grapevine inflorescences and véraison berries (Figures 2 and 4). The availability of carbon (C) and nitrogen (N) as well as the concentration of plant defence compounds in their diet affects herbivores in multiple ways (Awmack and Leather 2002) and the C/N ratio is crucial for the development of many insects, including *L. botrana* (Savopoulou‐Soultani et al. 1994). The nutritional quality with regard to amino acids as a nitrogen source was higher under elevated than ambient CO_2_, in ‘Cabernet Sauvignon’ than in ‘Riesling’, and in inflorescences than in véraison berries. Regarding sugars as energy sources, the nutritional quality was higher in véraison berries than in inflorescences. This diametrically opposed trend of N‐containing amino acid and C‐based sugar concentrations during advancing plant phenology is in line with previous reports (Bostanian, Vincent, and Isaacs 2012; Cuadros‐Inostroza et al. 2016). Based on the total concentration of phenolic compounds, inflorescences had a higher level of defence‐related compounds compared with véraison berries (Figures 2 and 4). Additionally, ‘Cabernet Sauvignon’ exhibited a higher defence level than ‘Riesling’. Phenolic compounds are ubiquitous in plants and deterrent and/or toxic effects on herbivores have been demonstrated for some of them, for example, quercetin, catechin, ferulic acid, caffeic acid and their derivatives which can negatively affect *Helicoverpa armigera* larvae (Schoonhoven, Van Loon, and Dicke 2005; War et al. 2013). Although the enzyme VvSHT ( *Vitis vinifera* spermidine *N*‐hydroxycinnamoyl transferase) has been reported in the literature (Roumani et al. 2021), this is the first detailed report of *N*,*N*′,*N*″‐tri‐hydroxycinnamoylspermidines in grapevine inflorescences. This enzyme's upregulation as a result of insect feeding has been reported in diverse plant species (Roumani et al. 2021). The occurrence of these compounds in grapevine inflorescences merits further investigation, as the structurally related *N*,*N*′‐di‐caffeoylspermidine can negatively affect *Manduca sexta* and *Spodoptera littoralis* larval growth (Kaur et al. 2010).

Although elevated CO_2_ concentration has been reported to increase concentrations of phenolic compounds in plants (Stiling and Cornelissen 2007; Lindroth 2010; Robinson, Ryan, and Newman 2012), in our experimental setting, elevated CO_2_ was found to increase the concentration of only one phenolic compound, whereas decreasing the concentration of two others (Table S6). This may be due to the relatively small increase in atmospheric CO_2_ (+20%) in the Geisenheim VineyardFACE.

### 4.2 Herbivore‐Related Host Plant Changes

Some herbivores are able to manipulate their host plant's biochemistry, rendering it a more beneficial environment by suppressing the plant's defence and/or optimising its nutrient content (Consales et al. 2012; Giron et al. 2016; Becker et al. 2021). In our experiment, the majority of insect‐essential amino acids was higher in berries that larvae had fed on whereas the concentrations of six phenolic compounds was lower (Figure 4; Table S6). This may indicate host plant defence suppression and nutrient content optimisation. At the same time, three other phenolic compounds were present in higher concentrations in berries that larvae had fed on which may indicate direct plant defence. Whether the plant defence response or its suppression is dominant depends on the specific toxicity of the respective phenolic acids, flavan‐3‐ols and flavonols for *L. botrana* which is unknown so far. More putatively herbivory‐induced compositional changes were observed in ‘Riesling’ than in ‘Cabernet Sauvignon’ which may be among the reasons for the varying susceptibilities of grapevine cultivars to *L. botrana* observed in the vineyard (Fermaud 1998). Which part of the observed compositional difference were actually due to larval food choice will pose an interesting topic for follow‐up experiments.

On the transcriptomic level, Reineke and Selim (2019) detected a stronger response of grapevine to *L. botrana* herbivory under elevated than under ambient CO_2_ concentrations. On the biochemical level, our analyses did not replicate this. In ‘Riesling’, putatively herbivory‐associated changes in biochemistry were less pronounced under elevated than under ambient CO_2_ concentration (Figure 4). Jasmonic acid (JA) plays a crucial role in plant defence against chewing insects like *L. botrana* larvae. As elevated CO_2_ can reduce JA‐signalling (DeLucia et al. 2012), it may have attenuated the plant's response. Both our study and Reineke and Selim (2019) were conducted in the same VineyardFACE investigating the effect of elevated CO_2_ on the interaction between grapevine and *L. botrana* at different plant phenological stages. However, other differences between the studies could explain why the results were not the same. While Reineke and Selim (2019) studied the plant transcriptome, here we studied plant biochemistry; while they studied the systemic defence response in leaves, here we studied the local defence response in reproductive organs.

### 4.3 Larval Development

Grapevine cultivar‐related differences are known to affect *L. botrana* life history traits such as larval development time, mating success, fecundity, fertility, female host choice, egg size and emergence phenology (Moreau, Benrey, and Thiéry 2006a, Moreau, Benrey, and Thiéry 2006b, Sharon et al. 2009, Thiéry, Monceau, and Moreau 2014). In their meta‐analysis, Stiling and Cornelissen (2007) report that larval RGR decreased when feeding on plants grown under eCO_2_. Our results show the same effect although it was only significant when feeding on inflorescences not on véraison berries (Figure 5). Higher RGR can indicate higher food quality and better larval performance (Bauce, Bidon, and Berthiaume 2002; Marlin et al. 2014), and lead to shorter development times which may reduce the window of vulnerability to predators (‘slow growth‐high mortality hypothesis’; Price et al. 1980). However, matters are complex and RGR is only one proxy that, in itself, only supplies limited information on actual fitness.

In our experiment, cultivar‐related differences in larval RGR were only significant when feeding on véraison berries but not on inflorescences. This might be related to biochemical changes at véraison when the berry colour changes and differences between red and white cultivars become more pronounced (also visible in Figure 2B). Grapevine phenology is also known to affect *L. botrana* life history traits, resulting for instance in larger females (Torres‐Vila et al. 2004), and increasing body weight and reproductive output with advancing plant phenology over the season (Torres‐Vila et al. 1999; Torres‐Vila and Rodríguez‐Molina 2013). We observed higher larval RGR on inflorescences than on véraison berries. This agrees with the reported shorter developmental time of larvae on inflorescences than on berries (Savopoulou‐Soultani et al. 1994; Torres‐Vila et al. 1999).

It is worthwhile mentioning that our larvae were reared in the laboratory which may limit the transferability of the results to wild populations. Furthermore, larvae were feeding on uniform artificial diet before being exposed to grapevine in the field. In several lepidopteran species, the quality of the parent's diet can have multiple effects on their offspring (for a review see Woestmann and Saastamoinen (2016)). Such trans‐generational effects were not part of the present study, but they would be interesting to address.

### 4.4 Larval Transcriptomic Response

Overall, transcriptomic analysis revealed an impact of CO_2_ concentration on the expression patterns of larval genes involved in defence responses, proteolysis, lipid transport, nutrient reservoir activity or signal transduction (Figure 6). A subset of these differentially expressed genes was successfully validated by qPCR (Figure S17). Of these eight genes, the expression of seven genes was downregulated in larvae feeding on elevated eCO_2_‐grapevine. Whether this translates, for instance, into reduced fitness, immune suppression or performance differences of *L. botrana* larvae under future elevated atmospheric CO_2_ conditions remains to be shown.

The diet of this multivoltine insect changes substantially between subsequent generations in the course of one season. Our study has shown that *L. botrana* larval gene expression patterns are markedly affected by advancing host plant phenology (Figure 7; Table 2). Considering that the number of significantly differentially expressed contigs was higher when larvae were feeding on inflorescences, they appear to be able to digest grapevine véraison berries more easily, using an array of digestive and detoxifying enzymes that are constitutively expressed. The transcription of digestive enzymes was affected to a larger degree than that of detoxifying enzymes (Table 2). Apart from the observed general pattern that larval transcriptomic plasticity mirrored host plant biochemical plasticity, we detected some specific links between plant nutritional and defence compounds and digestive and detoxifying enzymes. For instance, cytochrome P450‐like and glutathione‐S‐transferase‐like contigs were upregulated in larvae feeding on inflorescences compared with véraison berries which coincides with higher concentrations of phenolic compounds in inflorescences (Table 2). Yet, there were also exceptions: although ‘Cabernet Sauvignon’ contained higher concentrations of phenolic compounds than ‘Riesling’, we detected no corresponding upregulation of detoxifying enzymes. This apparent inconsistency may have several reasons: (1) some transcriptional changes regarding detoxifying enzymes may have been obscured because only about 40% of the detected contigs in this non‐model organism could be annotated; (2) adaptation may have happened on a physiological level different from transcription, for example, translation or later processes impacting enzyme activity directly, like midgut pH or redox conditions (Lazarević and Janković‐Tomanić 2015); (3) digestibility and/or toxicity of the plant compounds may have differed because of matrix effects related to co‐occurring biochemical changes linked to plant phenology and cultivars; (4) other mechanisms mitigating plant toxicity may have taken effect, such as rapid excretion and/or high toxin tolerance through target site insensitivity (Schoonhoven, Van Loon, and Dicke 2005). It would be very interesting to study the sensitivity of *L. botrana* to specific grapevine phenolics and to analyse their respective concentrations in larval frass.

Different cytochrome P450 isoforms responded differently to the treatments (Table 2). In larvae feeding on inflorescences compared with véraison berries, lower expression levels of cytochrome P450 4 g15 were accompanied by higher transcription levels of cytochrome P450 6B6‐like, indicating a finely tuned transcriptomic response to their diet. Especially members of the CYP6 families have been implicated in xenobiotic metabolism and pesticide resistance (Nauen et al. 2022). In several Lepidopteran species, the 6B6 isoform has been associated with resistance against pyrethroid or organophosphate insecticides (Li et al. 2020; Katsavou et al. 2022) and to the detoxification of plant defence compounds like nicotine and capsaicin (Hassanpour Farshour 2021, Katsavou et al. 2022). Yet, the 4 g15 isoform has also been connected to detoxification processes in the mosquito *Aedes aegyptii* (Diptera: Culicidae) and associated with pesticide resistance (Lien et al. 2019). Although it is difficult to predict the outcome of these transcriptional changes for the actual enzymatic activity, these results provide a potential link between larval diet and the detoxification of insecticides which may impact the efficacy of *L. botrana* chemical pest control.

Trypsin CFT‐1‐like is an enzyme responsible for the activation of the *Bacillus thuringiensis* (*Bt*) endotoxin in insects (Wang, Magoulas, and Hickey 1993). At present, *Bt* is a widely used plant protection agent used to control *L. botrana* larvae (Benelli et al. 2023b). The upregulation of trypsin CFT‐1‐like in larvae that were feeding on inflorescences compared with véraison berries (Table 2) might result in a higher efficacy of *Bt*‐based plant protection products against the first generation, that is, inflorescence‐feeding, larvae. In practice, however, it is rather the berry‐feeding generation against which these insecticides are applied. We observed a reduced larval transcription of trypsin CFT‐1‐like contigs under elevated CO_2_ only in one scenario: in larvae feeding on ‘Cabernet Sauvignon’ inflorescences. However, in case our results underestimate the effect (e.g., because of non‐annotated contigs), efficacy of *Bt*‐based plant protection products might decrease under future CO_2_ concentration.

The downregulation of the putative defence gene Hdd11 in larvae under elevated CO_2_ (on ‘Riesling’) and because of advanced host plant phenology (on aCO_2_‐‘Riesling’) might likewise have consequences for larval defences against biocontrol agents, possibly increasing their efficacy. Studies have shown that *L. botrana* larval defences can be affected by diet (Vogelweith et al. 2011; Thiéry et al. 2018). The effect of host plant phenology and of CO_2_ concentration, however, has not been studied yet. In the same scenarios, *L. botrana* population dynamics might be negatively affected because of the downregulation of the gene related to male sexual reproduction.

Further studies should investigate whether the observed transcriptomic plasticity is biologically relevant in the vineyard, resulting in changed larval defences against chemical and biological insecticides and in bottom‐up effects on natural enemies occurring in the vineyards.

Our results suggest that, in the near future of approximately 50 years, the grapevine‐*L. botrana* interaction will probably change less because of elevated CO_2_ concentrations than it does currently during one season. This might also be true for other highly relevant multivoltine pest insects like the pink cotton bollworm (*Pectinophora gossypiella* Saunders, Lepidoptera: Gelechiidae) or the codling moth (*Cydia pomonella* L., Lepidoptera: Tortricidae). Other climate change‐related impacts might be more dramatic for plant–insect interactions, for example, temperature‐related phenological mismatch, severe drought or flooding events, increasing levels of reactive gases like ozone and especially their interactions.

## 5 Conclusion

Generally, *L. botrana* larval gene expression patterns varied with host plant quality. Both plant biochemistry and the larval transcriptome displayed the highest plasticity because of advancing grapevine phenology whereas we did not detect a strong effect of moderately elevated CO_2_ concentrations (+20%). However, if the observed changes in larval gene expression related to advancing host plant phenology translate into biologically relevant differences, this may well be of consequence for the efficacy of currently applied pest management strategies against this grapevine pest insect, whether they are based on synthetic chemical insecticides, *Bt* or natural enemies. Climate change is a multifaceted process where many factors besides elevated CO_2_ concentrations are involved and interacting. Future studies on plant–insect interaction under climate change should take this into account.

## Author Contributions

C.B. and A.R. designed and conducted the experiment. H.V. analysed the transcriptomic data. C.B.S. conducted the LC – MS analyses and supervised quantitation of phenolic compounds. All authors interpreted the data. C.B., A.R., and C.B.S. wrote the manuscript. All authors approved the manuscript.

## Conflicts of Interest

The authors declare no conflicts of interest.

## Supporting information


**Figure S1.** Set up of the Geisenheim VineyardFACE (Free‐Air Carbon dioxide Enrichment) facility at Geisenheim University, Germany. (A) Schematic overview of one FACE ring structure, indicating the positions of the grapevine (*Vitis vinifera* L.) plants of the cultivars ‘Riesling’ (green dots) and ‘Cabernet Sauvignon’ (purple dots), respectively. (B) Close‐up view of one FACE ring structure highlighting the 36 jets mounted at 2.5 m height with fans for CO_2_ distribution and (C) areal overview of the whole VineyardFACE facility consisting of six ring structures (C), indicating the three rings with ambient CO_2_ (on average 409 ppm during the experimental periods described here) and three rings with elevated CO_2_ (on average 485 ppm during the experimental periods described here) and the CO_2_ tank.
**Table S2**. Weather conditions during the periods when experiments were conducted in the Geisenheim VineyardFACE facility in May, June, August, and September 2018. PAR: photosynthetically active radiation.
**Figure S3**. Carbon dioxide (CO_2_) concentrations in the VineyardFACE during the first (A) and second (B) experimental periods. Presented is the average value and standard deviation of all three ambient (Blue dots) and elevated (red dots) rings, respectively.
**Methods S4**. HPLC‐DAD‐ESI‐QTOF‐HR‐MS and ESI‐MS^n^ analyses of phenolic compounds.
**Table S5**. HPLC‐DAD‐ESI(−)‐QTOF‐HR‐MS and ESI(−)‐MS^n^ data of phenolic compounds extracted from *Vitis vinifera* ‘Riesling’ and ‘Cabernet Sauvignon’ inflorescences and véraison berries.
**Table S6**. Plant nutritional and defense compounds with significantly increased or decreased concentrations due to varying CO_2_ concentration, plant phenology and plant cultivar. Grapevine inflorescences (I) or véraison berries (B), of the cultivars ‘Riesling’ (R) and ‘Cabernet Sauvignon’ (CS), cultivated at ambient (409 ppm) or elevated (485 ppm) CO_2_ concentration in the VineyardFACE (*p* < 0.05, GLM, Tukey; *n* = 3). For mean concentrations, homogenous groups and statistics results, please see Tables S7–S12.
**Table S7**. Loadings of PCA presented in Figure 1A–D, regarding host plant quality parameters of the reproductive organs of grapevine cultivated in the VineyardFACE, depending on the atmospheric CO_2_ concentration, the cultivar, the plant phenology, and herbivory.
**Table S8**. Concentrations of amino acids in grapevine reproductive organs at two phenological stages (“inflorescences”, “véraison berries”) in the cultivars ‘Riesling’ (R) and ‘Cabernet Sauvignon’ (CS), cultivated at ambient (409 ppm) or elevated (485 ppm) CO_2_ concentration in the VineyardFACE, with or without herbivory of *L. botrana* larvae.
**Table S9**. Concentrations of sugars in grapevine reproductive organs at two phenological stages (“inflorescences”, “véraison berries”) in the cultivars ‘Riesling’ (R) and ‘Cabernet Sauvignon’ (CS), cultivated at ambient (409 ppm) or elevated (485 ppm) CO_2_ concentration in the VineyardFACE.
**Table S10**. Concentrations of phenolic compounds in grapevine reproductive organs in the cultivars (cv) ‘Riesling’ (R) and ‘Cabernet Sauvignon’ (CS), at different plant phenological stages (pp), cultivated at ambient (409 ppm) or elevated (485 ppm) CO_2_ concentration in the VineyardFACE.
**Table S11**. Concentrations of phenolic compounds in véraison berries with larval herbivory (h) by *Lobesia botrana* or without, of the cultivars (cv) ‘Riesling’ (R) and ‘Cabernet Sauvignon’ (CS) cultivated at ambient (409 ppm) or elevated (485 ppm) CO_2_ concentration in the VineyardFACE.
**Table S12**. (A, B) Details of the statistical evaluation of the concentrations of amino acids and sugars in grapevine reproductive organs as influenced by CO_2_ concentration (ambient: 409 ppm, elevated: 485 ppm), cultivar (‘Riesling’, ‘Cabernet Sauvignon’), and plant phenological stage (inflorescences, véraison).
**Table S13**. (A, B) Details of the statistical evaluation of the concentrations of phenolic compounds in grapevine reproductive organs as influenced by CO_2_ concentration (ambient: 409 ppm, elevated: 485 ppm), cultivar (‘Riesling’, ‘Cabernet Sauvignon’), and plant phenological stage (inflorescences, véraison).
**Table S14**. Metadata and raw RNA‐Seq sequence data accession numbers for 24 *L. botrana* samples obtained after larval feeding (1) at two different levels of CO_2_ concentration (a: ambient; e: elevated), (2) on two different host plant cultivars (R: Riesling; CS: Cabernet Sauvignon), and (3) at two different host plant phenological stages (F: inflorescences; V: véraison).
**Results S15**. Yield and annotation of RNA‐Seq contigs and results of validation per qPCR.
**Figure S16**. Top BLAST hit species distribution of the *L. botrana* transcriptome assembly. Top BLAST hit species distribution obtained by BLASTx against the NCBI non‐redundant (nr) protein database.
**Figure S17**. Confirmation of RNA‐Seq results (orange bars) by qPCR analysis. Data are reported as log2 fold changes in gene expression ± standard error of the mean (SEM) in *L. botrana* larvae which have fed on ‘Riesling’ (R) or ‘Cabernet Sauvignon’ (CS) grapevine grown under ambient and elevated CO2 conditions. A negative fold change indicates a lower expression in larvae which had fed on grapevine organs from elevated CO_2_ conditions, an asterisk indicates significant differences in pairwise comparisons (*p* < 0.05). Data obtained in qPCR were normalized to two housekeeping genes (actin and EF1α).
**Table S18**. Primer information for eight *L. botrana* differentially expressed genes and two housekeeping genes (EF1‐a and actin) used for validation of RNA‐Seq data by qPCR.
**Table S19**. Significantly differentially expressed contigs in *L. botrana* larvae feeding on inflorescences of ‘Riesling’ under ambient versus elevated CO_2_ concentration (EdgeR‐analysis).
**Table S20**. Significantly differentially expressed contigs in *L. botrana* larvae feeding on véraison berries of ‘Riesling’ under ambient versus elevated CO_2_ concentration (EdgeR‐analysis).
**Table S21**. Significantly differentially expressed contigs in *L. botrana* larvae feeding on inflorescences of ‘Cabernet Sauvignon’ under ambient versus elevated CO_2_ concentration (EdgeR‐analysis).
**Table S22**. Significantly differentially expressed contigs in *L. botrana* larvae feeding on véraison berries of ‘Cabernet Sauvignon’ under ambient versus elevated CO_2_ concentration (EdgeR‐analysis).
**Table S23**. Significantly differentially expressed contigs in *L. botrana* larvae feeding on inflorescences versus véraison berries of ‘Riesling’ under ambient CO_2_ concentration (EdgeR‐analysis).
**Table S24**. Significantly differentially expressed contigs in *L. botrana* larvae feeding on inflorescences versus véraison berries of ‘Riesling’ under elevated CO_2_ concentration (EdgeR‐analysis).
**Table S25**. Significantly differentially expressed contigs in *L. botrana* larvae feeding on inflorescences versus véraison berries of ‘Cabernet Sauvignon’ under ambient CO_2_ concentration (EdgeR‐analysis).
**Table S26**. Significantly differentially expressed contigs in *L. botrana* larvae feeding on inflorescences versus véraison berries of ‘Cabernet Sauvignon’ under elevated CO_2_ concentration (EdgeR‐analysis).
**Table S27**. Significantly differentially expressed contigs in *L. botrana* larvae feeding on inflorescences of ‘Riesling’ versus ‘Cabernet Sauvignon’ under ambient CO_2_ concentration (EdgeR‐analysis).
**Table S28**. Significantly differentially expressed contigs in *L. botrana* larvae feeding on inflorescences of ‘Riesling’ versus ‘Cabernet Sauvignon’ under elevated CO_2_ concentration (EdgeR‐analysis).
**Table S29**. Significantly differentially expressed contigs in *L. botrana* larvae feeding on véraison berries of ‘Riesling’ versus ‘Cabernet Sauvignon’ under ambient CO_2_ concentration (EdgeR‐analysis).
**Table S30**. Significantly differentially expressed contigs in *L. botrana* larvae feeding on véraison berries of ‘Riesling’ versus ‘Cabernet Sauvignon’ under elevated CO_2_ concentration (EdgeR‐analysis).

Data S2.

## Data Availability

The raw transcriptomic datasets generated during the current study are available in the NCBI Sequence Read Archive under BioProject PRJNA910346 and can be assessed in the BioSample database under accession numbers SAMN32123559‐SAMN32123582 (Table S14). The raw data on plant chemistry has been archived in the e!Dal repository under the DOI 10.5447/ipk/2024/11. Benefits Generated: Benefits from this research accrue from the sharing of our data and results on public databases as described above.
