# Supplementary material for: Change Your Diet: How CO2 , Plant Phenology and Genotype Alter Grapevine Quality and Affect Performance and Larval Transcriptome of an Insect Herbivore
Source: Mol Ecol. 2025 Jan 9;34(17):e17636. doi: 10.1111/mec.17636 (PMC12376960; doi:10.1111/mec.17636)
Supplement: Supplementary file 1 — Figure S1. Set up of the Geisenheim VineyardFACE (Free‐Air Carbon dioxide Enrichment) facility at Geisenheim University, Germany. (A) Schematic overview of one FACE ring structure, indicating the positions of the grapevine (Vitis vinifera L.) plants of the cultivars ‘Riesling’ (green dots) and ‘Cabernet Sauvignon’ (purple dots), respectively. (B) Close‐up view of one FACE ring structure highlighting the 36 jets mounted at 2.5 m height with fans for CO2 distribution and (C) areal overview of the whole VineyardFACE facility consisting of six ring structures (C), indicating the three rings with ambient CO2 (on average 409 ppm during the experimental periods described here) and three rings with elevated CO2 (on average 485 ppm during the experimental periods described here) and the CO2 tank. Table S2. Weather conditions during the periods when experiments were conducted in the Geisenheim VineyardFACE facility in May, June, August, and September 2018. PAR: photosynthetically active radiation. Figure S3. Carbon dioxide (CO2) concentrations in the VineyardFACE during the first (A) and second (B) experimental periods. Presented is the average value and standard deviation of all three ambient (Blue dots) and elevated (red dots) rings, respectively. Methods S4. HPLC‐DAD‐ESI‐QTOF‐HR‐MS and ESI‐MSn analyses of phenolic compounds. Table S5. HPLC‐DAD‐ESI(−)‐QTOF‐HR‐MS and ESI(−)‐MSn data of phenolic compounds extracted from Vitis vinifera ‘Riesling’ and ‘Cabernet Sauvignon’ inflorescences and véraison berries. Table S6. Plant nutritional and defense compounds with significantly increased or decreased concentrations due to varying CO2 concentration, plant phenology and plant cultivar. Grapevine inflorescences (I) or véraison berries (B), of the cultivars ‘Riesling’ (R) and ‘Cabernet Sauvignon’ (CS), cultivated at ambient (409 ppm) or elevated (485 ppm) CO2 concentration in the VineyardFACE (p < 0.05, GLM, Tukey; n = 3). For mean concentrations, homogenous groups and statistics res [file MEC-34-e17636-s002.docx]

**Supplemental Information for:**

**Change your diet: How CO_2_, plant phenology and genotype alter grapevine quality and affect performance and larval transcriptome of an insect herbivore**

Christine Becker, Christof B. Steingass, Heiko Vogel, Annette Reineke

**Table of Contents:**

| 1. VineyardFACE |  |
| --- | --- |
| Figure S1: Set up of the Geisenheim VineyardFACE (Free-Air Carbon dioxide Enrichment) facility at Geisenheim University, Germany. | Page 3 |
| Table S2. Weather conditions during the periods when experiments were conducted in the Geisenheim VineyardFACE | Page 3 |
| Figure S3. Carbon dioxide (CO_2_) concentrations in the VineyardFACE during the two experimental periods | Page 4 |
| 1. Plant Biochemistry |  |
| Methods S4: HPLC-DAD-ESI-QTOF-HR-MS and ESI-MS^n^ analyses of phenolic compounds. | Page 5 |
| Table S5: HPLC-DAD-ESI(−)-QTOF-HR-MS and ESI(−)-MS^n^ data of phenolic compounds | Page 6-7 |
| Table S6: Plant nutritional and defense compounds with significantly increased or decreased concentrations due to varying CO_2_ concentration, plant phenology and plant cultivar. | Page 8-9 |
| Table S8: Concentrations of amino acids in grapevine reproductive organs | Page 10-11 |
| Table S9: Concentrations of sugars in grapevine reproductive organs | Page 11 |
| Table S10: Concentrations of phenolic compounds in grapevine reproductive organs | Page 12-13 |
| Table S11: Concentrations of phenolic compounds in véraison berries with larval herbivory | Page 14 |
| 1. Larval transcriptome |  |
| Table S14. Metadata and raw RNA-Seq sequence data accession numbers for 24 *L. botrana* samples | Page 15 |
| Results S15: Yield and annotation of RNA-Seq contigs and results of validation per qPCR. | Page 16 |
| Figure S16: Top BLAST hit species distribution of the *L. botrana* transcriptome assembly. | Page 17 |
| Figure S17: Confirmation of RNA-Seq results by qPCR analysis. | Page 18-19 |
| Table S18: Primer information for *L. botrana* genes used for qPCR | Page 20 |
| Table S19: Significantly differentially expressed contigs in *L. botrana* larvae feeding on inflorescences of ‘Riesling’ under ambient *vs.* elevated CO_2_ concentration | Page 21-23 |
| Table S20: Significantly differentially expressed contigs in *L. botrana* larvae feeding on véraison berries of ‘Riesling’ under ambient *vs.* elevated CO_2_ concentration | Page 24 |
| Table S21: Significantly differentially expressed contigs in *L. botrana* larvae feeding on inflorescences of ‘Cabernet Sauvignon’ under ambient *vs.* elevated CO_2_ concentration | Page 24-26 |
| Table S22: Significantly differentially expressed contigs in *L. botrana* larvae feeding on véraison berries of ‘Cabernet Sauvignon’ under ambient *vs.* elevated CO_2_ concentration | Page 27-28 |
| Table S23: Significantly differentially expressed contigs in *L. botrana* larvae feeding on inflorescences *vs.* véraison berries of ‘Riesling’ under ambient CO_2_ concentration | Page 29-36 |
| Table S24: Significantly differentially expressed contigs in *L. botrana* larvae feeding on inflorescences *vs.* véraison berries of ‘Riesling’ under elevated CO_2_ concentration | Page 37-42 |
| Table S25: Significantly differentially expressed contigs in *L. botrana* larvae feeding on inflorescences *vs.* véraison berries of ‘Cabernet Sauvignon’ under ambient CO_2_ concentration | Page 43-29 |
| Table S26: Significantly differentially expressed contigs in *L. botrana* larvae feeding on inflorescences *vs.* véraison berries of ‘Cabernet Sauvignon’ under elevated CO_2_ concentration | Page 50-52 |
| Table S27: Significantly differentially expressed contigs in *L. botrana* larvae feeding on inflorescences of ‘Riesling’ vs. ‘Cabernet Sauvignon’ under ambient CO_2_ concentration | Page 53 |
| Table S28: Significantly differentially expressed contigs in *L. botrana* larvae feeding on inflorescences of ‘Riesling’ vs. ‘Cabernet Sauvignon’ under elevated CO_2_ concentration | Page 54-55 |
| Table S29: Significantly differentially expressed contigs in *L. botrana* larvae feeding on véraison berries of ‘Riesling’ vs. ‘Cabernet Sauvignon’ under ambient CO_2_ concentration | Page 56-57 |
| Table S30: Significantly differentially expressed contigs in *L. botrana* larvae feeding on véraison berries of ‘Riesling’ vs. ‘Cabernet Sauvignon’ under elevated CO_2_ concentration | Page 58 |

**
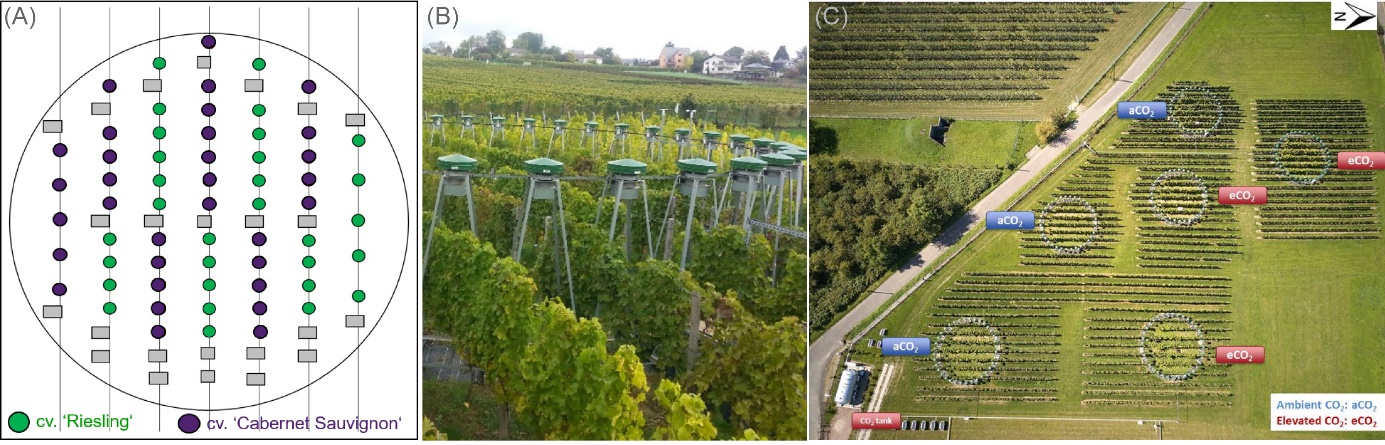
**

**Figure S1:** Set up of the Geisenheim VineyardFACE (Free-Air Carbon dioxide Enrichment) facility at Geisenheim University, Germany. (A) Schematic overview of one FACE ring structure, indicating the positions of the grapevine (*Vitis vinifera* L.) plants of the cultivars ‘Riesling’ (green dots) and ‘Cabernet Sauvignon’ (purple dots), respectively. (B) Close-up view of one FACE ring structure highlighting the 36 jets mounted at 2.5 m height with fans for CO_2_ distribution and (C) areal overview of the whole VineyardFACE facility consisting of six ring structures (C), indicating the three rings with ambient CO_2_ (on average 409 ppm during the experimental periods described here) and three rings with elevated CO_2_ (on average 485 ppm during the experimental periods described here) and the CO_2_ tank.

**Table S2.** Weather conditions during the periods when experiments were conducted in the Geisenheim VineyardFACE facility in May, June, August, and September 2018. PAR: photosynthetically active radiation

| **Experimental periods 2018** | **Air temperature [°C]** | | | **Precipitation** | **PAR [µmol m^-2^ s^-1^]** | |
| --- | --- | --- | --- | --- | --- | --- |
|  | average | min. | max. | average daily sum [mm] | average (during day) | max. |
| 29. May-5. Jun | 21.9 | 12.9 | 30.5 | 1.2 | 866.8 | 1992.5 |
| 30. May-6. Jun | 22.0 | 12.9 | 30.5 | 1.2 | 882.7 | 1992.5 |
|  |  |  |  |  |  |  |
|  |  |  |  |  |  |  |
| 9. Aug-16. Aug | 19.2 | 8.6 | 32.1 | 2.4 | 710.2 | 1914.7 |
| 10. Aug-17. Aug | 19.4 | 8.6 | 31.1 | 1.6 | 760.8 | 1914.7 |
|  |  |  |  |  |  |  |


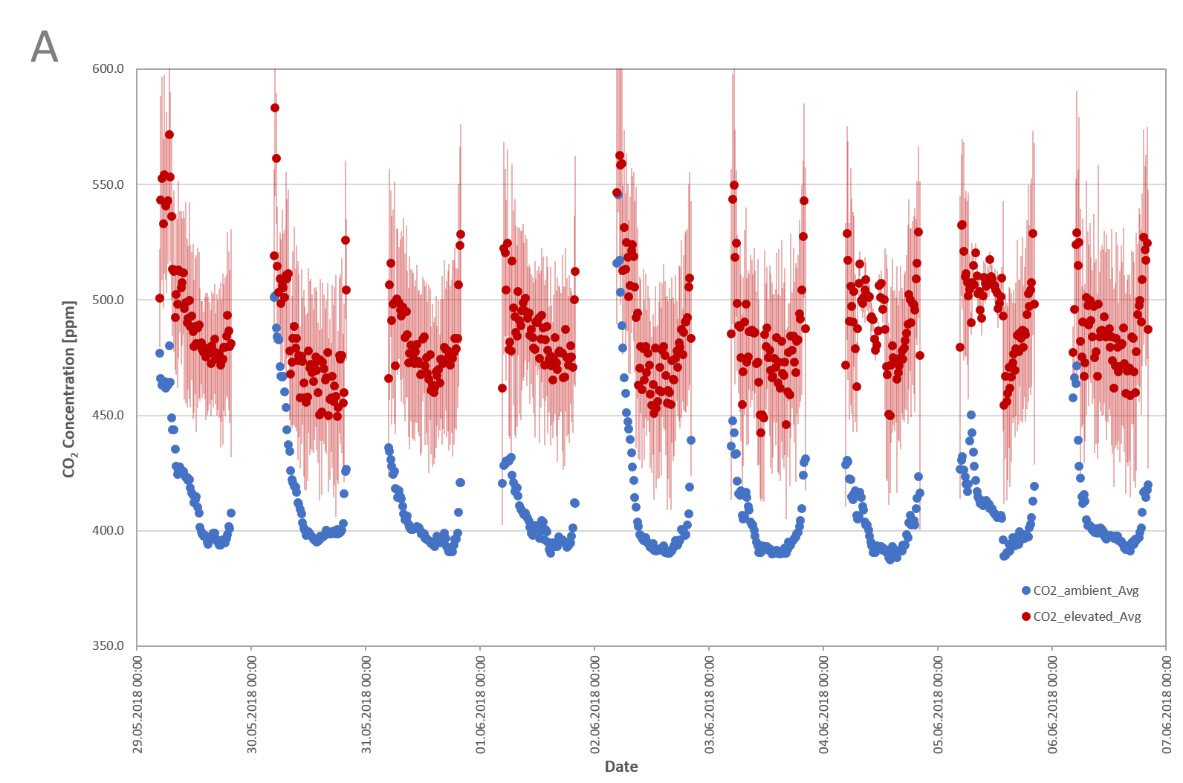

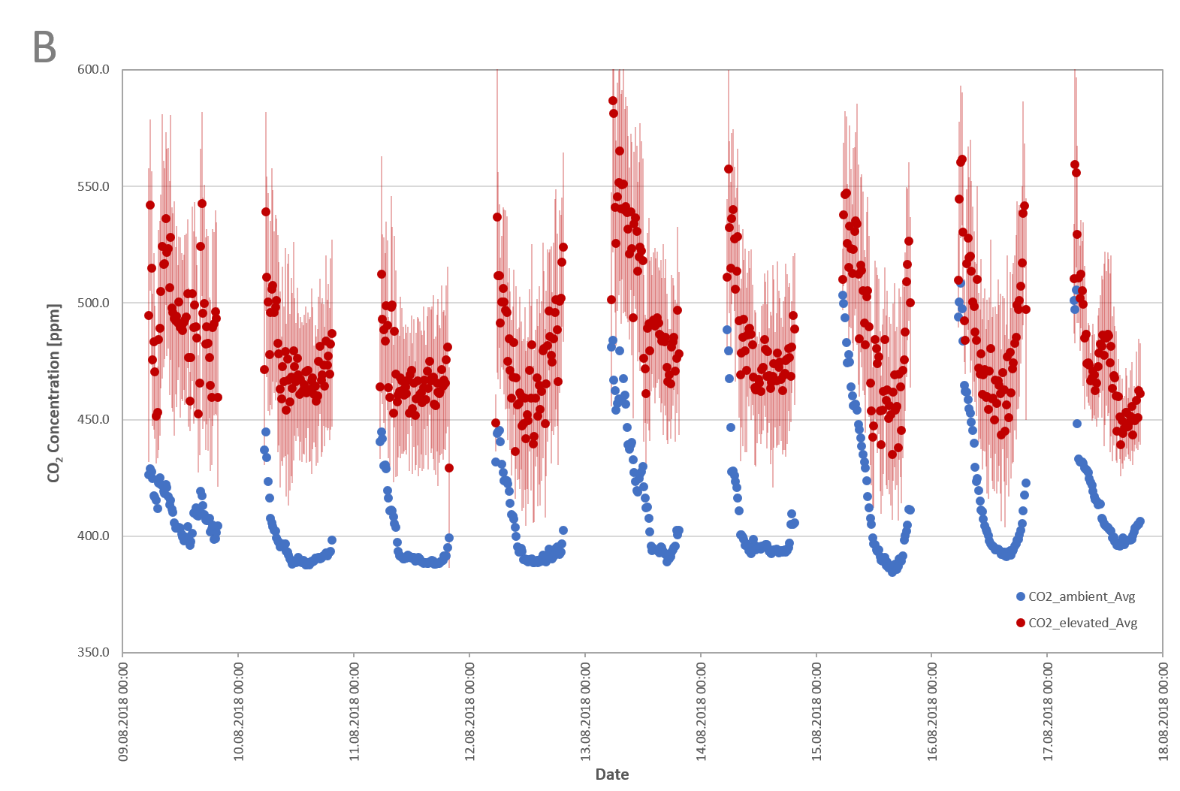


**Figure S3.** Carbon dioxide (CO_2_) concentrations in the VineyardFACE during the first (A) and second (B) experimental periods. Presented is the average value and standard deviation of all three ambient (blue dots) and elevated (red dots) rings, respectively.

**Methods S4: HPLC-DAD-ESI(**−)**-QTOF-HR-MS and ESI(**−)**-MS*^n^* analyses of phenolic compounds**

HPLC-DAD-ESI(−)-QTOF-HR-MS analyses of phenolic compounds were performed applying an Elute SP UHPLC system coupled in series to a *tims*TOF quadrupole time-of-flight mass spectrometer equipped with an electrospray ionization (ESI) source (all from Bruker Daltonik, Bremen, Germany). The HPLC was operated applying Compass HyStar version 5.1 software (Bruker). Control of the MS system and data evaluation were achieved with Bruker otofControl version 6.0 and Bruker Compass Data Analysis version 5.2 software, respectively. Chromatographic separation was achieved using a C18(2) reversed phase column (150 × 2.0 mm i.d., particle size *d*_p_ = 3 μm, Luna^®^, Phenomenex, Aschaffenburg, Germany) equipped with a guard column of the same material. Eluents A and B were H_2_O and acetonitrile/water (1:1, v/v), respectively, both containing 0.5% (v/v) formic acid. The gradient program was 4 to 50% B (20 min), 50 to 100% B (3.1 min), isocratic hold at 100% B (1.9 min), 100 to 4% B (0.1 min), and isocratic hold at 4% B (5.9 min). Total run time at a flow rate of 0.25 mL/min was 31 min, the injection volume 3 μL. Column oven temperature was set to 40 °C. Detection wavelength were 280, 320, 360, and 520 nm. UV/vis spectra were recorded at a scan speed of 5 Hz in the range of 220–600 nm.

ESI(−)-HR-MS spectra were recorded at a scan range of *m/z* 50–1,350. Nebulizing and dry gas was nitrogen at a pressure and flow rate of 2.5 bar and 7 L/min, respectively. The nebulizer temperature was set to 250 °C. The potential on the capillary was 3500 V. The HR-MS was calibrated using sodium formate clusters generated by 20 µL of the calibrant solution injected at the beginning of each analytical run.

HPLC-DAD-ESI(−)-MS*^n^* analyses were performed using an analytical system composed of an Accela autosampler, an Accela pump, and an Accela PDA diode array detector coupled on-line to an LXQ ion-trap mass spectrometer with an ESI source (all from Thermo Fisher Scientific, Dreieich, Germany). All HPLC settings were as detailed above. Mass spectra were recorded in the negative ion mode at a scan range of *m/z* 100–1,000 for MS1 and *m/z* 50–1,000 for MS*^n^* experiments. Nitrogen at 60, 1, and 10 arbitrary units served as sheath, auxiliary, and sweep gas, respectively. The source potential was 3.5 kV. Capillary temperature and voltage were 350 °C and 8.0 V, respectively. Tube lens offset was 10.0 V. The normalized collision energy for MS*^n^* fragmentations was set to 35% (activation Q: 0.25, activation time: 30 msec). Isolation widths was *m/z* 3.0. The system was controlled and data evaluation using XCalibur version 2.0.7 SP1 (Thermo Fisher Scientific). UV/vis spectra were recorded at a scan speed of 5 Hz in the range of 220–600 nm

Quantitation by HPLC-DAD was performed with an UltiMate 3000 (Thermo Fisher, Waltham, MA, USA) system applying the column and settings detailed above. Eluents were acidified with 2% acetic acid (v/v). Detection wavelengths were 280, 320, 360, and 520 nm. Linear calibration curves of authentic reference standards (gallic-, caffeic-, *p*-coumaric-, ferulic acid, (+)-catechin, (−)-epicatechin, procyanidin B1, B2, C1, quercetin 3-*O*-galactoside, quercetin 3-*O*-glucuronide, kaempferol 3-*O*-rutinoside) were used when available. If not, structurally related substances with molecular weight correction factors were used. Limits of detection and quantitation were estimated based on signal-to-noise ratios of 20:1 and 30:1, respectively.

**Table S5:** HPLC-DAD-ESI(−)-QTOF-HR-MS and ESI(−)-MS*^n^* data of phenolic compounds extracted from *Vitis vinifera* ‘Riesling’ and ‘Cabernet Sauvignon’ inflorescences and véraison berries

| No. | Proposed structure | *t*_R_ (min) | UV λ_max_ (nm) | [M−H]^− a^ (*m/z*) | Calc. *m/z* | Error (ppm) | Formula | MS^2^  (*m/z*) | MS^3^  (*m/z*) | MS^4^  (*m/z*) | λ_det._  (nm) |
| --- | --- | --- | --- | --- | --- | --- | --- | --- | --- | --- | --- |
| 1 | Galloyl hexose | 3.31 | 271 | 331.0671 | 331.0671 | -0.1 | C_13_H_15_O_10_^-^ | 168.9* | 150.9* | 106.8* | 280 |
| 2 | (Epi)gallocatechin | 5.29 | 274 | 305.0667 | 305.0667 | -0.2 | C_15_H_13_O_7_^-^ | 178.9* | 163.9* | 136.0* | 280 |
| 3a | *p*-Coumaric acid hexose | 7.09 | 320 | 325.0931 | 325.0929 | -0.6 | C_15_H_17_O_8_^-^ | 163.0, 119.1* | - | - | 320 |
| 3b | *+ cis-*Caffeoyl tartaric acid (*cis*-caftaric acid) |  |  | 311.0410 | 311.0409 | -0.6 | C_13_H_11_O_9_^-^ | 179.0, 148.9* | 102.8* | 58.8* |  |
| 4 | Caffeoyl tartaric acid (Caftaric acid) | 7.34 | sh294, 327 | 311.0409 | 311.0409 | -0.2 | C_13_H_11_O_9_^-^ | 178.9, 148.9* | 102.9* | 58.9* | 320 |
| 5 | Procyanidin B1^#^ | 8.69 | 279 | 577.1350 | 577.1351 | 0.3 | C_30_H_25_O_12_^-^ | 425.1* | 407.1* | 285.0* | 280 |
| 6 | (+)-Catechin^#^ | 10.19 | 278 | 289.0718 | 289.0718 | -0.3 | C_15_H_13_O_6_^-^ | 245.1* | 203.0* | 175.0* | 280 |
| 7 | *p*-Coumaroyl tartaric acid (coutaric acid) | 11.21 | 312 | 295.0459 | 295.0459 | 0.3 | C_13_H_11_O_8_^-^ | 162.9*, 148.9 | 118.9* | - | 320 |
| 8 | Procyanidin B2^#^ | 11.80 | 279 | 577.1350 | 577.1352 | -0.1 | C_30_H_25_O_12_^-^ | 425.2* | 407.1* | 285.0* | 280 |
| 9 | Feruloyl tartaric acid (fertaric acid) | 12.11 | 325 | 325.0568 | 325.0565 | -0.9 | C_14_H_13_O_9_^-^ | 193.0*, 148.8 | 148.9* | 133.9* | 320 |
| 10 | (−)-Epicatechin^#^ | 13.11 | 278 | 289.0718 | 289.0718 | -0.1 | C_15_H_13_O_6_^-^ | 245.1* | 203.0* | 188.0* | 280 |
| 11 | Procyanidin C1^#^ | 14.08 | 279 | 865.1988 | 865.1985 | -0.3 | C_45_H_37_O_18_^-^ | 695.2* | 543.2* | 525.2* | 280 |
| 12 | Procyanidin dimer gallate | 14.77 | 278 | 729.1461 | 729.1461 | 0.0 | C_37_H_29_O_16_^-^ | 577.1* | 559.1* | 285.1* | 280 |
| 13 | Quercetin 3-*O*-rutinoside^#^ | 17.12 | 278, 350 | 609.1463 | 609.1461 | -0.4 | C_27_H_29_O_16_^-^ | 301.0* | 178.9* | 151.0* | 360 |
| 14 | Quercetin 3-*O*-galactoside^#^ | 17.44 | 278, 350 | 463.0882 | 463.0882 | 0.0 | C_21_H_20_O_12_^-^ | 301.1* | 178.8* | 150.9* | 360 |
| 15 | (Epi)catechin gallate | 17.74 | 278 | 441.0827 | 441.0827 | 0.0 | C_22_H_17_O_10_^-^ | 289.0* | 245.0* | 188.0* | 280 |
| 16 | Quercetin 3-*O*-glucoside | 17.79 | 275, 353 | 463.0882 | 463.0882 | 0.0 | C_21_H_20_O_12_^-^ | 301.0* | 178.9* | 150.9* | 360 |
| 17 | Quercetin 3-*O*-glucuronide^#^ | 17.81 | 281, 352 | 477.0675 | 477.0675 | -0.1 | C_21_H_17_O_13_^-^ | 301.0* | 178.9* | 150.9* | 360 |
| 18a | Quercetin 3-*O*-(6ʺ-malonyl)hexoside | 20.48 | 277, 344 | 549.0889 | 549.0886 | -0.6 | C_24_H_21_O_15_^-^ | 505.1* | 301.0* | 179.0* | 360 |
| 18b | + Kaempferol 3-*O*-rutinoside^#^ |  |  | 593.1513 | 593.1512 | -0.1 | C_27_H_29_O_15_^-^ | 285.0* | 257.0* | 229.1* |  |
| 18c | + Kaempferol 3-*O*-galactoside |  |  | 447.0933 | 447.0933 | 0.1 | C_21_H_19_O_11_^-^ | - | - | - |  |
| 19 | Kaempferol 3-*O*-glucoside | 21.13 | 270, 345 | 447.0933 | 447.0933 | 0.1 | C_21_H_19_O_11_^-^ | 285.0* | 257.0* | 229.1* | 360 |
| 20 | Kaempferol 3-*O*-glucuronide | 21.40 | 269, 346 | 461.0725 | 461.0725 | 0.2 | C_21_H_17_O_12_^-^ | 285.0* | 257.0* | 229.1* | 360 |
| 21 | *N*^1^,*N^5^*,*N*^10^-Tri-caffeoylspermidine | 23.87 | 287, 319 | 630.2458 | 630.2457 | -0.1 | C_34_H_36_N_3_O_9_^-^ | 494.2, 468.3* | 332.1* | 289.1* | 320 |
| 22 | *N*,*N*ʹ,*N*ʺ-Di-caffeoyl-*p*-coumaroylspermidine (1) | 24.13 | 285, 313 | 614.2508 | 614.2508 | 0.2 | C_34_H_36_N_3_O_8_^-^ | 478.2* | 332.2* | 289.1* | 320 |
| 23 | *N*,*N*ʹ,*N*ʺ-Di-caffeoyl-*p*-coumaroylspermidine (2) | 24.38 | 295, 314 | 614.2508 | 614.2508 | 0.0 | C_34_H_36_N_3_O_8_^-^ | 478.3*, 452.2 | 358.1* | 315.2* | 320 |
| 24 | *N*,*N*ʹ,*N*ʺ-Di-caffeoyl-feruloylspermidine | 24.58 | 289, 319 | 644.2617 | 644.2614 | -0.6 | C_35_H_38_N_3_O_9_^-^ | 508.3*, 482.2 | 358.1* | 315.2* | 320 |
| 25 | *N*,*N*ʹ,*N*ʺ-Caffeoyl-di-*p*-coumaroylspermidine (1) | 24.76 | 294, 311 | 598.2559 | 598.2559 | -0.1 | C_34_H_36_N_3_O_7_^-^ | 478.3, 462.2* | 342.1* | 299.1* | 320 |
| 26 | *N*,*N*ʹ,*N*ʺ-Caffeoyl-di-*p*-coumaroylspermidine (2) | 24.83 | 293, 310 | 598.2559 | 598.2559 | 0.0 | C_34_H_36_N_3_O_7_^-^ | 478.3* | 358.1* | 315.2* | 320 |
| 27 | *N*,*N*ʹ,*N*ʺ-Caffeoyl-*p*-coumaroyl-feruloylspermidine | 24.94 | 287, 308 | 628.2664 | 628.2664 | 0.1 | C_35_H_38_N_3_O_8_^-^ | 508.2*, 492.2, 478.3 | 358.1* | 315.2* | 320 |
| 28 | *N*^1^*,N*^5^*,N*^10^-Tri-*p*-coumaroylspermidine | 25.21 | 293, 310 | 582.2608 | 582.2610 | 0.3 | C_34_H_36_N_3_O_6_^-^ | 462.2*, 436.2 | 342.1* | 299.1* | 320 |
| 29 | *N*,*N*ʹ,*N*ʺ-Di-*p*-coumaroyl-feruloylspermidine | 25.36 | 291, 308 | 612.2716 | 612.2715 | -0.1 | C_35_H_38_N_3_O_7_^-^ | 492.3* | 449.2* | 299.3* | 320 |
| 30 | Naringenin chalcone^#^ | 25.47 | 368 | 271.0612 | 271.0612 | 0.0 | C_15_H_11_O_5_^-^ | 150.8* | 106.8* | 64.8* | 360 |

^a^ Phenolic compounds were detected as deprotonated molecules ([M−H]^−^). Base peaks are labelled with asterisks (*)

Assignment of compounds labelled by # were verified using authentic reference standards.

**Table S6**: Plant nutritional and defense compounds with significantly increased or decreased concentrations due to varying CO_2_ concentration, plant phenology and plant cultivar. Grapevine inflorescences (I) or véraison berries (B), of the cultivars ‘Riesling’ (R) and ‘Cabernet Sauvignon’ (CS), cultivated at ambient (409 ppm) or elevated (485 ppm) CO_2_ concentration in the VineyardFACE (p<0.05, GLM, Tukey; n=3). For mean concentrations, homogenous groups and statistics results, please see Tables S7-S10 and Tables S11-S12.

|  |  | nutritional compounds | | defense compounds (phenolic compounds) | | | | |
| --- | --- | --- | --- | --- | --- | --- | --- | --- |
| impact factor |  | **amino acids** | **sugars** | **phenolic acids** | **flavan-3-ols** | **flavonols** | **chalcone** | **phenolamides** |
| CO_2_ | **conc. higher under elevated CO_2_** | Ile  Lys  Gly  Tyr (in R)  Met (in R) |  |  |  | Q3Glc |  |  |
|  | **conc. higher under ambient CO_2_** |  |  |  |  | K3Glc  K3Glucuro |  |  |
| plant phenology | **conc. higher in inflorescences** | Ile  Lys  Met  Val  Trp  Ala  Ser  Gly  His (in R)  Pro (in R) |  | *p*-coum.tart. acid  ferul.tart. acid  gall.hexose (in R)  caff.tart. acid  *p*-coumaric acid hexose + *cis*-caff.tart. acid (in CS) | procyanidin B1  procyanidin B2  procyanidin C1  procyanidin dimer gallate (in R)  catechin (in R)  epicatechin (in R)  (epi)catechin gallate (in R) | Q3Glc  Q3Glucuro  Q3Gal  Q3R  Q3MH+K3R+K3Gal  K3Glc  K3Glucuro | naringenin chalcone | TCaffSp  DCaffCoumSp DCaffCoumSp2  DCaffFerS  CaffDCoumSp1  CaffDCoumSp2  CaffCoumFerSp  TCoumSp  DCoumFerSp |
|  | **conc. higher in berries** | Arg | glucose  fructose | gall. hexose (in CS) | catechin (in CS)  epicatechin (in CS) (epi)catechin gallate (in CS) |  |  |  |
| cultivar | **conc. higher in Riesling** | (Thr: tendency in I) | glucose (in I)  fructose (in I) | caff.tart. acid (in berries)  *p*-coumaric acid hexose + *cis*-caff.tart. acid (in I)  ferul.tart. acid (in B) | procyanidin B2 (in I) | Q3MH+K3R+K3Gal (in I)  K3Glc (in I)  K3Glucuro (in I) |  | DCaffCoumSp2 (in I)  CaffDCoumSp1 (in I)  CaffDCoumSp2 (in I) DCaffCoumSp1 (in I)  TCoumSp (in I) |
|  | **conc. higher in Cabernet Sauvignon** | Ile  Val  Tyr  Trp  Arg  Gly  Phe (in B)  His (in B)  Pro (in B)  Met (under aCO_2_) |  | *p*-coum.tart. acid  gall. hexose (in B)  *p*-coumaric acid hexose + *cis*-caff.tart. acid (in B) | procyanidin B1 (in B)  procyanidin B2 (in B)  procyanidin C1 (in B)  procyanidin dimer gallate (in B)  catechin (in B)  epicatechin (in B)  (epi)catechin gallate (in B)  (epi)gallocatechin | Q3Glc (in B)  Q3Glucur (in B)  Q3Gal (in B)  Q3R (in eCO_2_-berries) | naringenin chalcone (in I) | DCaffFerS (in I)  CaffCoumFerSp (in I)  DCoumFerSp (in I) |
| herbivory  (in berries only) | **conc. higher under herbivory** | Val  Lys  Trp  Arg  Pro  Ala  Ser  His (in R)  Met (in aCO_2_-R) |  | gall. hexose (in R) | procyanidin B1 (in R)  procyanidin B2 (in R) |  |  |  |
|  | **conc. lower under herbivory** |  |  | caff.tart. acid  coumaric acid hexose + *cis*-caff.tart. acid (in aCO_2_-R) | (epi)gallocatechin | Q3Glc  Q3Glucuro |  |  |

Arg: arginine, Pro: proline, Trp: tryptophan, Leu: leucine, Lys: lysine, His: histidine, Met: methionine, Phe: phenylalanine, Thr: threonine, Val: valine, Tyr: tyrosine, Ile: isoleucine, Ser: serine, Gly: glycine, Ala: alanine, caff. : caffeoyl, coum.: coumaroyl, ferul.: feruloyl, gall.: galloyl, tart.: tartaric, Q3R: quercetin 3-*O*-rutinoside, Q3Gal: quercetin 3-*O*-galactoside, Q3Glc: quercetin 3-*O*-glucoside, Q3Glucuro: quercetin 3-*O*-glucuronide, Q3MH: quercetin 3-*O*-(6ʺ-malonyl)hexoside, K3R: Kaempferol 3-*O*-rutinoside, K3Gal: Kaempferol 3-*O*-galactoside, K3Glc: Kaempferol 3-*O*-glucoside, K3Glucuro: Kaempferol 3-*O*-glucuronide, TCaffSp: *N*^1^,*N*^5^,*N*^10^-Tri-caffeoylspermidine, DCaffCoumSp1: *N,N*ʹ*,N*ʺ-Di-caffeoyl-*p*-coumaroylspermidine 1, DCaffCoumSp2: *N,N*ʹ*,N*ʺ-Di-caffeoyl-*p*-coumaroylspermidine 2, DCaffFerS: *N,N*ʹ*,N*ʺ-Di-caffeoyl-feruloylspermidine, CaffDCoumSp1: *N,N*ʹ*,N*ʺ-Caffeoyl-di-*p*-coumaroylspermidine 1, CaffDCoumSp2: *N,N*ʹ*,N*ʺ-Caffeoyl-di-*p*-coumaroylspermidine 2, CaffCoumFerSp: *N,N*ʹ*,N*ʺ-Caffeoyl-coumaroyl-feruloylspermidine, TCoumSp: *N^1^,N^5^,N^10^*-Tri-*p*-coumaroylspermidine, DCoumFerSp: *N,N*ʹ*,N*ʺ-Di-*p*-coumaroyl-feruloylspermidine

**Table** **S8**: Concentrations of amino acids in grapevine reproductive organs in the cultivars (cv) ‘Riesling’ (R) and ‘Cabernet Sauvignon’ (CS), cultivated at ambient (409 ppm) or elevated (485 ppm) CO_2_ concentration in the VineyardFACE. The upper part of the table shows the impact of plant phenology (pp), the lower part shows the impact of herbivory (h) on véraison berries. Values represent the mean concentration per treatment (n=3) in mg kg^-1^ dry matter. Asterisks indicate the factors or interactions that had a significant impact (GLM). Different lower case, upper case or Greek letters, respectively, indicate significant differences in one row.

|  |  | | | |  | | | |  | statistics – impact of: | | | | | | |
| --- | --- | --- | --- | --- | --- | --- | --- | --- | --- | --- | --- | --- | --- | --- | --- | --- |
| pp | **inflorescences** | | | | **véraison berries** | | | |  | **CO_2_** | **cv** | **pp** | **CO_2_ * cv** | **CO_2_ * pp** | **cv * pp** | **CO_2_ * cv * pp** |
| CO_2_ | **ambient** | | **elevated** | | **ambient** | | **elevated** | |  |  |  |  |  |  |  |  |
| cv | **R** | **CS** | **R** | **CS** | **R** | **CS** | **R** | **CS** |  |  |  |  |  |  |  |  |
| Ala | 103 A | 173 A | 170 A | 178 A | 70 B | 90 B | 91 B | 109 B |  |  |  | * |  |  |  |  |
| Gly | 9 A b β | 19 A a β | 16 A b α | 22 A a α | 8 B b β | 8 B a β | 8 B b α | 9 B a α |  | * | * | * |  |  |  |  |
| Ser | 134 A | 286 A | 239 A | 315 A | 63 B | 45 B | 79 B | 51 B |  |  | * | * |  |  | * |  |
| Arg | 93 B b | 241 B a | 159 B b | 282 B a | 230 A b | 517 A a | 384 A b | 602 A a |  |  | * | * |  |  |  |  |
| His | 73 a | 134 a | 113 a | 154 a | 42 b | 112 a | 61 b | 122 a |  |  | * | * |  |  | * |  |
| Ile | 15 A b β | 28 A a β | 26 A b α | 30 A a α | 11 B b β | 18 B a β | 14 B b α | 24 B a α |  | * | * | * |  |  |  |  |
| Leu | 25 A b | 45 A ab | 43 A ab | 49 A a | 10 B b | 31 A ab | 12 B ab | 37 A a |  |  | * | * | * |  | * |  |
| Lys | 65 A β | 79 A β | 113 A α | 90 A α | 7 B β | 11 B β | 10 B α | 13 B α |  | * |  | * |  |  |  |  |
| Met | 3 A b | 6 A a | 7 A a | 7 A a | 2 B b | 5 B a | 4 B a | 5 B a |  | * | * | * | * |  |  |  |
| Phe | 17 ab | 21 ab | 24 ab | 21 ab | 11 b | 24 a | 17 b | 28 a |  |  | * |  |  |  | * |  |
| Pro | 304 a | 407 a | 523 a | 466 a | 121 b | 411 a | 167 b | 340 a |  |  |  | * |  |  | * |  |
| Thr | 50 ab | 108 a | 89 ab | 116 a | 81 ab | 50 b | 110 ab | 62 b |  |  |  |  |  |  | * |  |
| Trp | 38 A b | 113 A a | 55 A b | 117 A a | 36 B b | 52 B a | 51 B b | 75 B a |  |  | * | * |  |  |  |  |
| Tyr | 8 c | 28 a | 17 b | 30 a | 8 c | 25 a | 11 b | 35 a |  | * | * |  | * |  |  |  |
| Val | 39 A b | 75 A a | 64 A b | 82 A a | 11 B a | 17 B b | 13 B a | 23 B b |  |  | * | * |  |  |  |  |
|  |  |  |  |  |  |  |  |  |  |  |  |  |  |  |  |  |
| h | **no herbivory** | | | | **herbivory** | | | |  | **h** | **CO_2_** | **cv** | **h * CO_2_** | **h * cv** | **CO_2_ * cv** | **h * CO_2_ * cv** |
| CO_2_ | **ambient** | | **elevated** | | **ambient** | | **elevated** | |  |  |  |  |  |  |  |  |
| cv | R | **CS** | **R** | **CS** | **R** | **CS** | **R** | **CS** |  |  |  |  |  |  |  |  |
| Ala | 70 B | 90 B | 91 B | 109 B | 122 A | 140 A | 134 A | 136 A |  | * |  |  |  |  |  |  |
| Gly | 8 | 8 | 8 | 9 | 7 | 8 | 8 | 10 |  | * |  | * |  |  |  |  |
| Ser | 63 B a | 45 B b | 79 B A a | 51 B b | 104 A a | 68 A b | 105 A a | 74 A b |  |  |  |  |  |  |  |  |
| Arg | 230 B | 517 B | 384 B | 602 B | 535 A | 783 A | 669 A | 922 A |  | * |  |  |  |  |  |  |
| His | 42 c | 112 ab | 61 c | 122 ab | 92 b | 134 a | 87 b | 149 a |  | * |  | * |  | * |  |  |
| Ile | 10.8 b | 17.7 a | 13.6 b | 23.8 a | 14.8 b | 19.9 a | 16.6 b | 22.4 a |  |  |  | * |  |  |  |  |
| Leu | 9.7 b | 31.5 a | 12.1 b | 37.0 a | 14.8 b | 34.7 a | 15.6 b | 39.3 a |  |  |  | * |  |  |  |  |
| Lys | 7.3 B b | 10.7 B a | 10.2 B b | 12.9 B a | 12.3 A b | 14.6 A a | 12.6 A b | 17.6 A a |  | * |  | * |  |  |  |  |
| Met | 1.8 b | 5.3 a | 4.0 ab | 4.5 a | 4.4 a | 4.8 a | 5.4 a | 5.3 a |  |  |  |  |  | * |  | * |
| Phe | 11.4 b | 23.7 a | 16.8 b | 28.0 a | 16.7 b | 22.9 a | 22.5 b | 31.3 a |  |  |  | * |  |  |  |  |
| Pro | 121 B | 411 B | 167 B | 340 B | 223 A | 562 A | 241 A | 577 A |  | * |  | * |  |  |  |  |
| Thr | 80.7 a | 50.3 b | 110.5 a | 61.9 b | 112.5 a | 65.5 b | 122.1 a | 72.0 b |  |  |  | * |  |  |  |  |
| Trp | 36.4 B β | 52.3 B β | 51.1 B α | 74.9 B α | 62.6 A β | 66.6 A β | 75.0 A α | 76.4 A α |  | * | * |  |  |  |  |  |
| Tyr | 7.5 b | 25.0 a | 11.2 b | 34.6 a | 14.1 b | 28.7 a | 13.2 b | 32.0 a |  |  |  | * |  |  |  |  |
| Val | 11.3 B b | 16.8 B a | 13.2 B b | 23.0 B a | 17.0 A b | 22.3 A a | 20.0 A b | 27.7 A a |  | * |  | * |  |  |  |  |

Arg: arginine, Pro: proline, Trp: tryptophan, Leu: leucine, Lys: lysine, His: histidine, Met: methionine, Phe: phenylalanine, Thr: threonine, Val: valine, Tyr: tyrosine, Ile: isoleucine, Ser: serine, Gly: glycine, Ala: alanine.

**Table** **S9**: Concentrations of sugars in grapevine reproductive organs in the cultivars ‘Riesling’ (R) and ‘Cabernet Sauvignon’ (CS), cultivated at ambient (409 ppm) or elevated (485 ppm) CO_2_ concentration in the VineyardFACE. Values represent the mean concentration per treatment (n=3) in mg g^-1^ dry matter. Asterisks indicate the factors or interactions that had a significant impact (GLM). Different letters indicate significant differences in one row. NA: not analyzed; the concentration of sucrose could only be measured in inflorescences samples because the ratio between glucose and sucrose was too high in the véraison berry-samples.

|  |  | | | |  | | | |  | statistics – impact of: | | | | | | |
| --- | --- | --- | --- | --- | --- | --- | --- | --- | --- | --- | --- | --- | --- | --- | --- | --- |
| pp | **inflorescences** | | | | **véraison berries** | | | |  | **CO_2_** | **cv** | **pp** | **CO_2_ * cv** | **CO_2_ * pp** | **cv * pp** | **CO_2_ * cv * pp** |
| CO_2_ | **ambient** | | **elevated** | | **ambient** | | **elevated** | |  |  |  |  |  |  |  |  |
| cv | **R** | **CS** | **R** | **CS** | **R** | **CS** | **R** | **CS** |  |  |  |  |  |  |  |  |
| sucrose | 3 | 3.6 | 4 | 3.5 | NA | NA | NA | NA |  |  |  | - |  | - | - | - |
| glucose | 17 b | 13 c | 16 b | 14 c | 261 a | 233 a | 268 a | 232 a |  |  | * | * |  |  | * |  |
| fructose | 13 b | 9 c | 12 b | 9 c | 225 a | 197 a | 232 a | 193 a |  |  | * | * |  |  | * |  |

**Table** **S10**: Concentrations of phenolic compounds in grapevine reproductive organs in the cultivars (cv) ‘Riesling’ (R) and ‘Cabernet Sauvignon’ (CS), at different plant phenological stages (pp), cultivated at ambient (409 ppm) or elevated (485 ppm) CO_2_ concentration in the VineyardFACE. Values represent the mean concentration per treatment (n=3) in mg g^-1^ dry matter. Asterisks indicate the factors or interactions that had a significant impact (GLM). These significant differences in one row are indicated in detail by different lower and upper case letters, respectively.

|  |  |  |  |  |  |  |  |  |  |  | statistics – impact of: | | | | | | |
| --- | --- | --- | --- | --- | --- | --- | --- | --- | --- | --- | --- | --- | --- | --- | --- | --- | --- |
|  | **pp** | **inflorescences** | | | | **véraison berries** | | | |  | **CO_2_** | **cv** | **pp** | **CO_2_ * cv** | **CO_2_ * pp** | **cv* pp** | **CO_2_ * cv * pp** |
|  | **CO_2_** | **ambient** | | **elevated** | | **ambient** | | **elevated** | |  |  |  |  |  |  |  |  |
| compound class | **cv** | **R** | **CS** | **R** | **CS** | **R** | **CS** | **R** | **CS** |  |  |  |  |  |  |  |  |
| phenolic acids | galloyl hexose | 0.07 b | 0.08 b | 0.08 b | 0.07 b | 0.05 c | 0.19 a | 0.05 c | 0.24 a |  |  | * | * |  |  | * |  |
|  | caffeoyl tartaric acid | 4.28 b | 6.67 a | 4.42 b | 6.77 a | 2.25 c | 1.26 d | 1.91 c | 1.32 d |  |  | * | * |  |  | * |  |
|  | *p*-coumaric acid hexose + *cis*-caffeoyl tartaric acid | 0.06 bc | 0.18 a | 0.07 bc | 0.17 a | 0.08 b | 0.03 c | 0.06 b | 0.05 c |  |  | * | * |  |  | * |  |
|  | *p*-coumaroyl tartaric acid | 1.47 B a | 1.65 A a | 1.48 B a | 1.71 A a | 0.88 B b | 0.92 A b | 0.74 B b | 0.99 A b |  |  | * | * |  |  |  |  |
|  | feruloyl tartaric acid | 0.28 a | 0.18 b | 0.28 a | 0.18 b | 0.12 c | 0.04 d | 0.11 c | 0.05 d |  |  | * | * |  |  | * |  |
| flavan-3-ols | procyanidin B1 | 2.08 a | 2.55 a | 2.45 a | 2.84 a | 0.82 c | 1.77 b | 0.75 c | 2.02 b |  |  | * | * |  |  | * |  |
|  | procyanidin B2 | 2.04 a | 1.45 b | 2.80 a | 1.61 b | 0.74 c | 1.71 ab | 0.66 c | 1.89 ab |  |  |  | * |  |  | * |  |
|  | procyanidin C1 | 2.70 a | 2.42 a | 2.71 a | 2.54 a | 0.88 c | 1.72 b | 0.80 c | 1.91 b |  |  | * | * |  |  | * |  |
|  | procyanidin dimer gallate | 5.91 a | 4.43 a | 6.18 a | 4.92 a | 2.10 b | 4.88 a | 1.97 b | 4.20 a |  |  |  | * |  |  | * |  |
|  | (epi)gallocatechin | 0.23 b | 0.27 a | 0.25 b | 0.32 a | 0.24 b | 0.29 a | 0.23 b | 0.33 a |  |  | * |  |  |  |  |  |
|  | catechin | 3.87 b | 3.56 b | 3.84 b | 3.99 b | 1.77 c | 5.32 a | 1.53 c | 5.61 a |  |  | * |  |  |  | * |  |
|  | epicatechin | 1.26 b | 1.35 b | 1.45 b | 1.44 b | 1.16 c | 3.71 a | 0.94 c | 3.77 a |  |  | * | * |  |  | * |  |
|  | (epi)catechin gallate | 3.73 b | 3.78 b | 3.70 b | 3.95 b | 1.84 c | 5.82 a | 1.62 c | 6.91 a |  |  | * |  |  |  | * |  |
| chalcone | naringenin chalcone | 0.09 b A | 0.21 a A | 0.07 b A | 0.24 a A | 0^#^ B | 0^#^ B | 0^#^ B | 0^#^ B |  |  | * | * |  |  |  |  |
| flavonols | Q3R | 0.46 a | 0.28 a | 0.43 a | 0.27 a | 0.02 bc | 0.03 b | 0.02 c | 0.04 b |  |  | * | * |  |  | * | * |
|  | Q3Gal | 0.12 a | 0.11 a | 0.11 a | 0.11 a | 0.01 c | 0.04 b | 0.01 c | 0.05 b |  |  | * | * |  |  | * |  |
|  | Q3Glc | 0.22 a β | 0.23 a β | 0.28 a α | 0.26 a α | 0.03 c β | 0.13 b β | 0.04 c α | 0.17 b α |  | * | * | * |  |  | * |  |
|  | Q3Glucuro | 9.90 a | 8.46 a | 9.30 a | 8.20 a | 0.21 c | 0.36 b | 0.18 c | 0.43 b |  |  | * | * |  |  | * | * |
|  | Q3MH+ K3R + K3Gal | 0.27 a A | 0.13 b A | 0.25 a A | 0.13 b A | 0 B | 0 B | 0 B | 0 B |  |  | * | * |  |  |  |  |
|  | K3Glc | 0.06 a A α | 0.03 b A α | 0.05 a A β | 0.03 b A β | 0 B | 0 B | 0 B | 0 B |  | * | * | * |  |  |  |  |
|  | K3Glucuro | 0.16 a A α | 0.08 b A α | 0.15 a A β | 0.07 b A β | 0 B | 0 B | 0 B | 0 B |  | * | * | * |  |  |  |  |
| phenolamides | TCaff Sp | 0.23 a | 0.20 a | 0.20 a | 0.23 a | 0^#^ b | 0^#^ b | 0^#^ b | 0^#^ b |  |  |  | * | * |  |  |  |
|  | DCaff CoumSp 1 | 0.08 A a | 0.07 B a | 0.08 A a | 0.07 B a | 0^#^ b | 0^#^ b | 0^#^ b | 0^#^ b |  |  | * | * |  |  |  |  |
|  | DCaff CoumSp 2 | 0.27 A a | 0.19 B a | 0.25 A a | 0.22 B a | 0^#^ b | 0^#^ b | 0^#^ b | 0^#^ b |  |  | * | * |  |  |  |  |
|  | DCaff FerSp | 0.07 B a | 0.10 A a | 0.06 B a | 0.12 A a | 0^#^ b | 0^#^ b | 0^#^ b | 0^#^ b |  |  | * | * |  |  |  |  |
|  | Caff DCoumSp 1 | 0.10 A a | 0.05 B a | 0.08 A a | 0.07 B a | 0^#^ b | 0^#^ b | 0^#^ b | 0^#^ b |  |  | * | * |  |  |  |  |
|  | Caff DCoumSp 2 | 0.20 A a | 0.12 B a | 0.17 A a | 0.15 B a | 0^#^ b | 0^#^ b | 0^#^ b | 0^#^ b |  |  | * | * |  |  |  |  |
|  | CaffCoumFerSp | 0.01 B a | 0.04 A a | 0.01 B a | 0.06 A a | 0^#^ b | 0^#^ b | 0^#^ b | 0^#^ b |  |  | * | * |  |  |  |  |
|  | TCoumSp | 0.08 A a | 0.05 B a | 0.07 A a | 0.06 B a | 0^#^ b | 0^#^ b | 0^#^ b | 0^#^ b |  |  | * | * |  |  |  |  |
|  | DCoumFerSp | 0 b | 0.03 a | 0 b | 0.04 a | 0^#^ b | 0^#^ b | 0^#^ b | 0^#^ b |  |  | * | * |  |  | * |  |
| Total |  | 40.3 a | 39.0 a | 41.3 a | 40.9 a | 13.2 c | 28.2 b | 11.6 c | 30.0 b |  |  | * | * |  |  | * |  |

Q3R: quercetin 3-*O*-rutinoside, Q3Gal: quercetin 3-*O*-galactoside, Q3Glc: quercetin 3-*O*-glucoside, Q3Glucuro: quercetin 3-*O*-glucuronide, Q3MH: quercetin 3-*O*-(6ʺ-malonyl)hexoside, K3R: Kaempferol 3-*O*-rutinoside, K3Gal: Kaempferol 3-*O*-galactoside, K3Glc: Kaempferol 3-*O*-glucoside, K3Glucuro: Kaempferol 3-*O*-glucuronide. TCaffSp: *N*^1^,*N*^5^,*N*^10^-Tri-caffeoylspermidine, DCaffCoumSp 1: *N,N*ʹ*,N*ʺ-Di-caffeoyl-*p*-coumaroylspermidine 1, DCaffCoumSp 2: *N,N*ʹ*,N*ʺ-Di-caffeoyl-*p*-coumaroylspermidine 2, DCaffFerS: *N,N*ʹ*,N*ʺ-Di-caffeoyl-feruloylspermidine, CaffDCoumSp 1: *N,N*ʹ*,N*ʺ-Caffeoyl-di-*p*-coumaroylspermidine 1, CaffDCoumSp 2: *N,N*ʹ*,N*ʺ-Caffeoyl-di-*p*-coumaroylspermidine 2, CaffCoumFerSp: *N,N*ʹ*,N*ʺ-Caffeoyl-coumaroyl-feruloylspermidine, TCoumSp: *N^1^,N^5^,N^10^*-Tri-*p*-coumaroylspermidine, DCoumFerSp: *N,N*ʹ*,N*ʺ-Di-*p*-coumaroyl-feruloylspermidine. ^#^: not detected

**Table S11**: Concentrations of phenolic compounds in véraison berries with larval herbivory (h) by *Lobesia botrana* or without, of the cultivars (cv) ‘Riesling’ (R) and ‘Cabernet Sauvignon’ (CS) cultivated at ambient (409 ppm) or elevated (485 ppm) CO_2_ concentration in the VineyardFACE. Values represent the mean concentration per treatment (n=3) in mg g^-1^ dry matter. Asterisks indicate the factors or interactions that had a significant impact (GLM). These significant differences in one row are indicated in detail by different lower and upper case letters, respectively.

|  |  |  |  |  |  |  |  |  |  | statistics - impact of: | | | | | | |
| --- | --- | --- | --- | --- | --- | --- | --- | --- | --- | --- | --- | --- | --- | --- | --- | --- |
|  | **h** | **no herbivory** | | | | **herbivory** | | | | **h** | **CO_2_** | **cv** | **h* CO_2_** | **h* cv** | **CO_2_* cv** | **h* CO_2_* cv** |
|  | **CO_2_** | **ambient** | | **elevated** | | **ambient** | | **elevated** | |  |  |  |  |  |  |  |
| compound class | **cv** | **R** | **CS** | **R** | **CS** | **R** | **CS** | **R** | **CS** |  |  |  |  |  |  |  |
| phenolic acids | galloyl hexose | 0.05 c | 0.19 a | 0.05 c | 0.24 a | 0.07 b | 0.24 a | 0.08 b | 0.25 a |  |  | ***** |  | ***** |  |  |
|  | caffeoyl tartaric acid | 2.25 A a | 1.26 A b | 1.91 A a | 1.32 A b | 1.64 B a | 1.33 B b | 1.75 B a | 1.19 B b | ***** |  | ***** |  |  |  |  |
|  | *p*-coumaric acid hexose + cis-caffeoyl tartaric acid | 0.08 a | 0.03 b | 0.06 ab | 0.05 b | 0.04 b | 0.04 b | 0.04 b | 0.04 b | ***** |  | ***** |  | ***** |  | ***** |
|  | p-coumaroyl tartaric acid | 0.88 b | 0.92 a | 0.74 b | 0.99 a | 0.70 b | 1.00 a | 0.73 b | 0.90 a |  |  | ***** |  |  |  |  |
|  | feruloyl tartaric acid | 0.12 a | 0.04 b | 0.11 a | 0.05 b | 0.10 a | 0.05 b | 0.11 a | 0.05 b |  |  | ***** |  |  |  |  |
| flavan-3-ols | procyanidin B1 | 0.82 c | 1.77 a | 0.75 c | 2.02 a | 0.95 b | 1.96 a | 1.12 b | 1.98 a |  |  | ***** |  | ***** |  |  |
|  | procyanidin B2 | 0.74 c | 1.71 a | 0.66 c | 1.89 a | 0.96 b | 1.97 a | 1.07 b | 2.00 a | ***** |  | ***** |  | ***** |  |  |
|  | procyanidin C1 | 0.88 b | 1.72 a | 0.80 b | 1.91 a | 1.03 b | 1.89 a | 1.14 b | 1.84 a |  |  | ***** |  |  |  |  |
|  | procyanidin dimer gallate | 2.10 b | 4.88 a | 1.97 b | 4.20 a | 2.72 b | 5.46 a | 2.96 b | 5.42 a |  |  | ***** |  |  |  |  |
|  | (epi)gallocatechin | 0.24 A b | 0.29 A a | 0.23 A b | 0.33 A a | 0.18 B b | 0.27 B a | 0.18 B b | 0.25 B a | ***** |  | ***** |  |  |  |  |
|  | catechin | 1.77 b | 5.32 a | 1.53 b | 5.61 a | 1.92 b | 5.25 a | 1.99 b | 4.99 a |  |  | ***** |  |  |  |  |
|  | epi-catechin | 1.16 c | 3.71 a | 0.94 c | 3.77 a | 1.39 b | 3.87 a | 1.43 b | 3.62 a |  |  | ***** |  | ***** |  |  |
|  | (epi)catechin gallate | 1.84 b | 5.82 a | 1.62 b | 6.91 a | 2.24 b | 5.66 a | 2.28 b | 5.57 a |  |  | ***** |  | ***** |  |  |
| flavonols | Q3R | 0.02 ab | 0.03 ab | 0.02 ab | 0.04 a | 0.01 b | 0.03 ab | 0.02 ab | 0.03 ab |  |  | ***** |  |  |  | ***** |
|  | Q3Gal | 0.01 b | 0.04 a | 0.01 b | 0.05 a | 0.00 b | 0.03 a | 0.01 b | 0.02 a |  |  | ***** |  |  |  |  |
|  | Q3Glc | 0.03 c | 0.13 a | 0.04 c | 0.17 a | 0.01 d | 0.07 b | 0.01 d | 0.07 b | ***** |  | ***** |  | ***** |  |  |
|  | Q3Glucuro | 0.21 B a | 0.36 A a | 0.18 B a | 0.43 A a | 0.12 B b | 0.29 A b | 0.16 B b | 0.28 A b | ***** |  | ***** |  |  |  |  |

Q3R: quercetin 3-*O*-rutinoside, Q3Gal: quercetin 3-*O*-galactoside, Q3Glc: quercetin 3-*O*-glucoside, Q3Glucuro: quercetin 3-*O*-glucuronide, Q3MH: quercetin 3-*O*-(6ʺ-malonyl)hexoside

**Table S14:** Metadata and raw RNA-Seq sequence data accession numbers for 24 *L. botrana* samples obtained after larval feeding (1) at two different levels of CO_2_ concentration (a: ambient; e: elevated), (2) on two different host plant cultivars (R: ‘Riesling’; CS: ‘Cabernet Sauvignon’), and (3) at two different host plant phenological stages (F: flowering; V: véraison). Three RNA pools (indicated by 1, 2, 3) representing 3 biological replicates (each from one FACE ring) for each CO_2_ concentration, cultivar and phenological stage were sequenced.

| **Sample ID** | **CO_2_ level** | **Host plant cultivar** | **Host plant phenology** | **BioSample accessions** |
| --- | --- | --- | --- | --- |
| aR_F1 | ambient | Riesling | Flowering | SAMN32123559 |
| aR_F2 | ambient | Riesling | Flowering | SAMN32123560 |
| aR_F3 | ambient | Riesling | Flowering | SAMN32123561 |
| aR_V1 | ambient | Riesling | Véraison | SAMN32123562 |
| aR_V2 | ambient | Riesling | Véraison | SAMN32123563 |
| aR_V3 | ambient | Riesling | Véraison | SAMN32123564 |
| eR_F1 | elevated | Riesling | Flowering | SAMN32123565 |
| eR_F2 | elevated | Riesling | Flowering | SAMN32123566 |
| eR_F3 | elevated | Riesling | Flowering | SAMN32123567 |
| eR_V1 | elevated | Riesling | Véraison | SAMN32123568 |
| eR_V2 | elevated | Riesling | Véraison | SAMN32123569 |
| eR_V3 | elevated | Riesling | Véraison | SAMN32123570 |
| aCS_F1 | ambient | Cabernet Sauvignon | Flowering | SAMN32123571 |
| aCS_F2 | ambient | Cabernet Sauvignon | Flowering | SAMN32123572 |
| aCS_F3 | ambient | Cabernet Sauvignon | Flowering | SAMN32123573 |
| aCS_V1 | ambient | Cabernet Sauvignon | Véraison | SAMN32123574 |
| aCS_V2 | ambient | Cabernet Sauvignon | Véraison | SAMN32123575 |
| aCS_V3 | ambient | Cabernet Sauvignon | Véraison | SAMN32123576 |
| eCS_F1 | elevated | Cabernet Sauvignon | Flowering | SAMN32123577 |
| eCS_F2 | elevated | Cabernet Sauvignon | Flowering | SAMN32123578 |
| eCS_F3 | elevated | Cabernet Sauvignon | Flowering | SAMN32123579 |
| eCS_V1 | elevated | Cabernet Sauvignon | Véraison | SAMN32123580 |
| eCS_V2 | elevated | Cabernet Sauvignon | Véraison | SAMN32123581 |
| eCS_V3 | elevated | Cabernet Sauvignon | Véraison | SAMN32123582 |

**Results S15:** The *de novo* assembly of the combined RNA-Seq datasets of 24 pools of *L. botrana* larvae that were feeding on grapevine reproductive organs from the different treatments yielded 61,294 contigs (minimum 3x coverage cut-off criteria) with an N50 contig length of 1,025 bp. Of these contigs, 24,229 (39.5%) could be annotated. The top 10 insect species with the highest number of best NCBI (nr database) BLAST hits against the assembled *L. botrana* contigs were other Lepidoptera for which full length genome sequences are available such as *Helicoverpa armigera, Trichoplusia ni*, and *Plutella xylostella*. Figure S16 shows the complete list of species with top BLAST hits.

From the genes which were significantly differentially expressed under elevated compared to ambient CO_2_ concentrations, a set of eight genes was selected to validate the RNA-Seq results via qPCR. A combination of two *L. botrana* housekeeping genes (elongation factor 1-alpha and actin) with consistent expression levels was found to be suitable as reference for normalization of gene expression (*M* = 0.253, CV = 0.088). In general, the qPCR results confirmed the differences in gene expression levels indicated by RNA-Seq analysis (Figure S17). For larvae which were feeding on ‘Riesling’ inflorescences, expression of four genes were assessed in qPCR both with the same RNA pools initially used for RNA-Seq analysis as well as with a separate batch of individual larvae. The gene expression levels indicated by RNA-Seq were consistent across all qPCR samples, with three genes (chemosensory protein CSP10, putative defense protein Hdd11 and tyrosine hydroxylase) being down and one gene (apolipophorin isoform X1) upregulated in larvae feeding on elevated compared to ambient CO_2_-‘Riesling’ inflorescences (Figure S17A). Consistent expression trends between RNA-Seq and qPCR were obtained for four additional genes, of which two each were downregulated in larvae feeding on ‘Cabernet Sauvignon’ inflorescences (trypsin CFT-1-like protein and arylphorin precursor, Figure S17B) and on ‘Cabernet Sauvignon’ véraison berries (juvenile hormone-suppressible protein 1-like and a probable Ras GTPase-activating protein, Figure S17C), respectively.


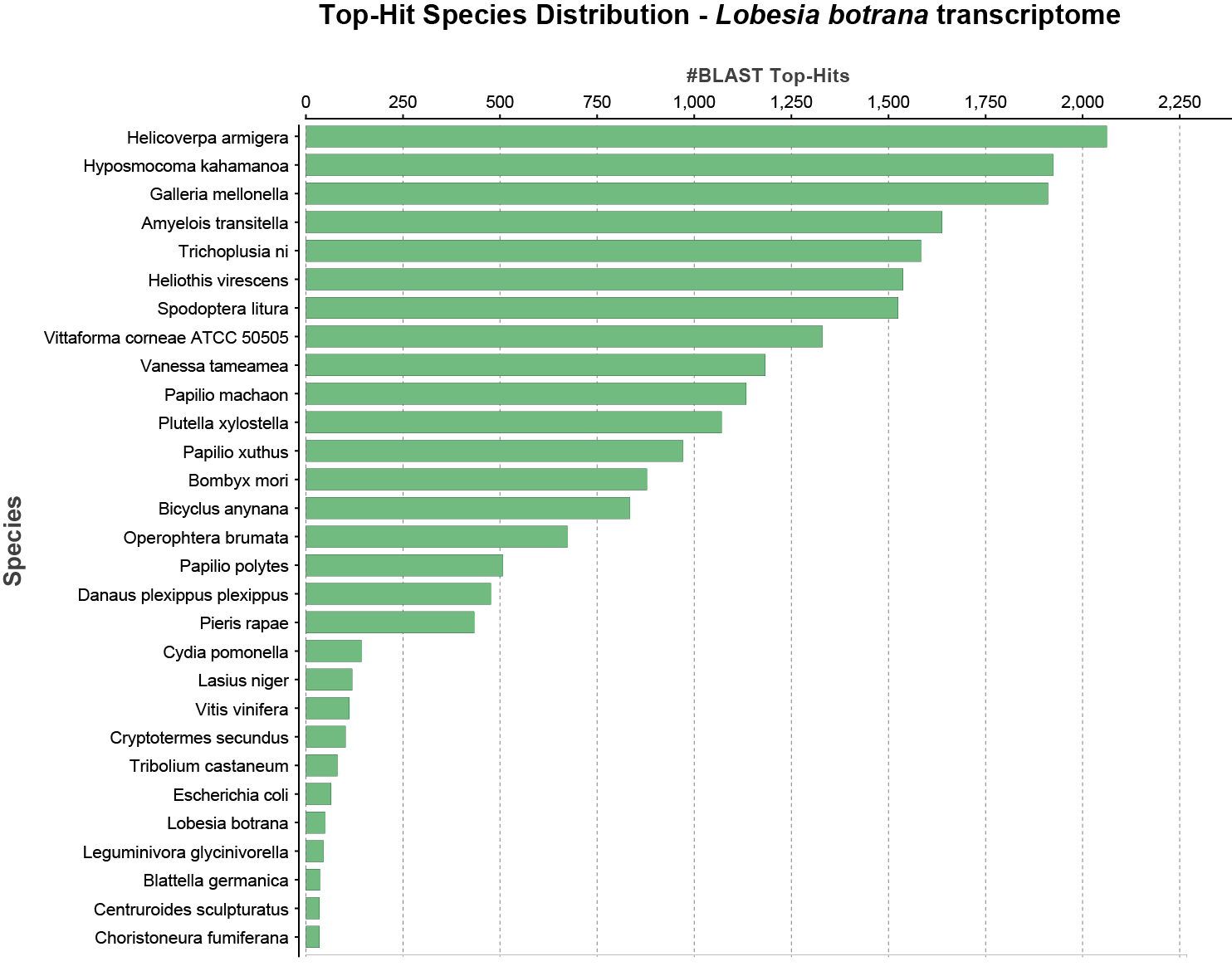


**Figure S16:** Top BLAST hit species distribution of the *L. botrana* transcriptome assembly. Top BLAST hit species distribution obtained by BLASTx against the NCBI non-redundant (nr) protein database. The number of top BLAST hits per species is shown on the x-axis.


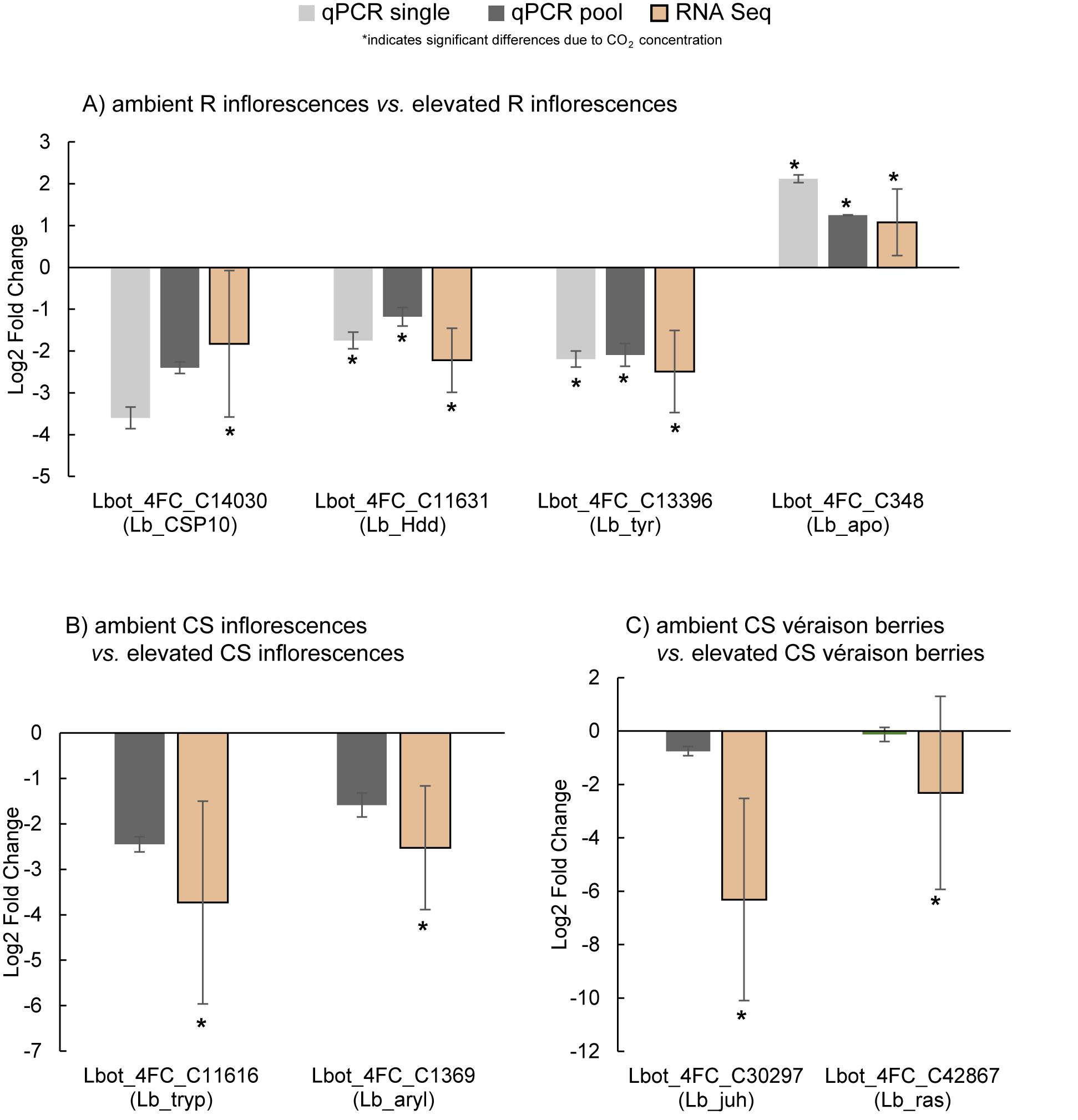


**Figure S17**: Confirmation of RNA-Seq results (orange bars) by qPCR analysis. Data are reported as log2 fold changes in gene expression ± standard error of the mean (SEM) in *L. botrana* larvae which have fed on ‘Riesling’ (R) or ‘Cabernet Sauvignon’ (CS) grapevine grown under ambient and elevated CO_2_ conditions. A negative fold change indicates a lower expression in larvae which had fed on grapevine organs from elevated CO_2_ conditions, an asterisk indicates significant differences in pairwise comparisons (*p* < 0.05). Data obtained in qPCR were normalized to two housekeeping genes (actin and EF1α).

A) Log2 fold changes in gene expression in *L. botrana* larvae which had fed on ‘Riesling’ inflorescences. qPCR was performed with single *L. botrana* larvae (light grey bars) as well as with the same RNA pools used for RNA-Seq analysis (dark grey bars). Top blast descriptors are as follows: CSP10 is a chemosensory protein; Hdd is putative defense protein Hdd11; tyr is a tyrosine hydroxylase and apo is apolipophorins isoform X1.

B) Log2 fold changes in gene expression in *L. botrana* larvae which had fed on ‘Cabernet Sauvignon’ inflorescences. qPCR was performed with the same RNA pools used for RNA-Seq analysis (dark grey bars). Top blast descriptors are as follows: tryp is a trypsin CFT-1-like protein and aryl is arylphorin precursor.

C) Log2 fold changes in gene expression in *L. botrana* larvae which had fed on ‘Cabernet Sauvignon’ berries at véraison. qPCR was performed with the same RNA pools used for RNA-Seq analysis (dark grey bars). Top blast descriptors are as follows: juh is a juvenile hormone-suppressible protein 1-like and ras is a probable Ras GTPase-activating protein.

**Table S18:** Primer information for eight *L. botrana* differentially expressed genes and two housekeeping genes (EF1α and actin) used for validation of RNA-Seq data by qPCR.

| **Contig** | **Putative Gene and Gene Code** | **Primer Sequence Forward** | **Primer Sequence Reverse** | **Efficiency (%)** | **R^2^** |
| --- | --- | --- | --- | --- | --- |
| Lbot_4FC_C11631 | putative defense protein Hdd11 (Lb_Hdd) | 5’- GATTCCACTCCCACCCAGAA -3’ | 5’- CGAGAAAGACGACCAGCAGA -3’ | 98 | 0.995 |
| Lbot_4FC_C348 | apolipophorins isoform X1 (Lb_apo) | 5’- TCCTGGATTTGGAGCTCGTT -3’ | 5’- ATCAGTGCTGGGTACAAGCT -3’ | 97 | 0.997 |
| Lbot_4FC_C13396 | tyrosine hydroxylase (Lb_tyr) | 5’- AGAGTTCGCCAATGGAGGAA -3’ | 5’- CTAGCTTCGCCCAGTTCTCT -3’ | 100.7 | 0.997 |
| Lbot_4FC_C14030 | chemsosensory protein CSP10 (Lb_CSP10) | 5’- TACTTGTCAGCGGCAAACAC -3’ | 5’- TTGAGTGACGGTCGCAAAAG -3’ | 116.2 | 0.979 |
| Lbot_4FC_C11616 | trypsin CFT-1-like (Lb_tryp) | 5’- ACCGTCATCTCCTTCAGCAA -3’ | 5’- ATCCTACAGCCCACACAGAC -3’ | 101.7 | 0.996 |
| Lbot_4FC_C42867 | probable Ras GTPase-activating protein isoform X6 (Lb_ras) | 5’- CGTTGTCGGGCATAGTGAAG -3’ | 5’- TCGCAGCTCAACTACCAGAA -3’ | 101.2 | 0.991 |
| Lbot_4FC_C30297 | basic juvenile hormone-suppressible protein 1-like (Lb_juh) | 5’- ATATCCCTCATGCCATGCGA -3’ | 5’- AAGGAGCGGAGAGGAGAAAC -3’ | 105.3 | 0.992 |
| Lbot_4FC_C1369 | arylphorin precursor (Lb_aryl) | 5’- AACGGAGATGTAGACCCTGC-3‘ | 5’- TCATCGTTGCCTGTTACCCA-3‘ | 101.9 | 0.999 |
|  | EF1α | 5’- TGGTACATCACAGGCCGATT -3’ | 5’- TTTCCTCGAAACGGGACTCA -3’ | 98.1 | 0.999 |
|  | Actin | 5’- TACTCTTTCACCACCACCGC -3’ | 5’- TCGGGAAGCTCGTAGGACTT -3’ | 98.9 | 0.999 |

**Results EDGE test**

**Table S19:** Significantly differentially expressed contigs in *L. botrana* larvae feeding on inflorescences of ‘Riesling’ under ambient *vs.* elevated CO_2_ concentration (EdgeR-analysis). A negative fold change indicates that expression levels were lower in larvae feeding on eCO_2_-plants.

| Feature ID | Fold change | Weighted difference | p-value (FDR corrected) | Sequence Description | Blast Top Hit Taxonomy Name | Annotation GO ID | Annotation GO Term |
| --- | --- | --- | --- | --- | --- | --- | --- |
| Lbot_4FC_C6445 | -38.25 | -1.51E-05 | <0.0001 | arylphorin precursor | *Leguminivora glycinivorella* | GO:0005615;GO:0045735 | extracellular space;nutrient reservoir activity |
| Lbot_4FC_C37066 | -34.43 | -9.76E-06 | <0.0001 | arylphorin subunit beta-like | *Leguminivora glycinivorella* | GO:0005615;GO:0045735 | extracellular space;nutrient reservoir activity |
| Lbot_4FC_C3540 | -20.36 | -7.46E-06 | 0.0118 | ---NA--- |  |  |  |
| Lbot_4FC_C48141 | -12.49 | -8.95E-06 | 0.0048 | uncharacterized protein LOC113512554 | *Galleria mellonella* |  |  |
| Lbot_4FC_C15311 | -10.29 | -1.06E-05 | 0.0011 | cuticle protein 3 | *Amyelois transitella* | GO:0042302 | structural constituent of cuticle |
| Lbot_4FC_C9736 | -8.01 | -1.58E-05 | <0.0001 | cuticle protein 1-like | *Papilio xuthus* | GO:0005576;GO:0016021 | extracellular region;integral component of membrane |
| Lbot_4FC_C38667 | -7.09 | -1.66E-05 | <0.0001 | ---NA--- |  |  |  |
| Lbot_4FC_C12628 | -6.98 | -1.65E-05 | <0.0001 | cell wall protein RBR3-like | *Amyelois transitella* | GO:0042302 | structural constituent of cuticle |
| Lbot_4FC_C10477 | -5.78 | -1.44E-05 | 0.0003 | ---NA--- |  |  |  |
| Lbot_4FC_C13396 | -5.71 | -1.49E-05 | 0.0003 | tyrosine hydroxylase | *Vanessa tameamea* | GO:0004511; GO:0005506; GO:0009072; GO:0042423; GO:0055114 | tyrosine 3-monooxygenase activity; iron ion binding; aromatic amino acid family metabolic process; catecholamine biosynthetic process;oxidation-reduction process |
| Lbot_4FC_C57559 | -5.48 | -1.24E-05 | 0.0047 | tyrosine hydroxylase | *Heliothis virescens* | GO:0004511;GO:0005506;GO:0009072;GO:0042423;GO:0055114 | tyrosine 3-monooxygenase activity;iron ion binding;aromatic amino acid family metabolic process;catecholamine biosynthetic process;oxidation-reduction process |
| Lbot_4FC_C60087 | -5.18 | -1.46E-05 | 0.0008 | hypothetical protein B5X24_HaOG206415 | *Helicoverpa armigera* |  |  |
| Lbot_4FC_C11631 | -4.77 | -1.38E-05 | 0.0018 | putative defense protein Hdd11 | *Heliothis virescens* | GO:0005576; GO:0042742; GO:0042832; GO:0045087 | extracellular region; defense response to bacterium; defense response to protozoan; innate immune response |
| Lbot_4FC_C36854 | -4.06 | -1.68E-05 | 0.0007 | mediator of RNA polymerase II transcription subunit 15-like | *Amyelois transitella* |  |  |
| Lbot_4FC_C8166 | -3.76 | -1.22E-05 | 0.0387 | flexible cuticle protein 12-like | *Plutella xylostella* | GO:0042302 | structural constituent of cuticle |
| Lbot_4FC_C14030 | -3.65 | -1.23E-05 | 0.0279 | ejaculatory bulb-specific protein 3-like isoform X2 | *Lobesia botrana* |  |  |
| Lbot_4FC_C60932 | -3.51 | -2.16E-05 | 0.0003 | larval cuticle protein 1-like | *Choristoneura fumiferana* | GO:0003677;GO:0005664;GO:0006260;GO:0042302 | DNA binding;nuclear origin of replication recognition complex;DNA replication;structural constituent of cuticle |
| Lbot_4FC_C20712 | -3.34 | -2.50E-05 | 0.0001 | larval cuticle protein LCP-17-like | *Hyposmocoma kahamanoa* | GO:0042302 | structural constituent of cuticle |
| Lbot_4FC_C5107 | -3.04 | -7.22E-05 | <0.0001 | endocuticle structural glycoprotein ABD-5-like | *Pieris rapae* | GO:0042302 | structural constituent of cuticle |
| Lbot_4FC_C850 | -3.02 | -1.68E-05 | 0.0126 | ---NA--- |  |  |  |
| Lbot_4FC_C60285 | -2.91 | -3.28E-05 | <0.0001 | larval cuticle protein LCP-17-like | *Spodoptera litura* | GO:0042302 | structural constituent of cuticle |
| Lbot_4FC_C4427 | -2.72 | -3.46E-05 | 0.0001 | larval cuticle protein LCP-17-like | *Papilio polytes* | GO:0042302 | structural constituent of cuticle |
| Lbot_4FC_C55 | -2.48 | -7.80E-05 | <0.0001 | flexible cuticle protein 12-like | *Nicrophorus vespilloides* | GO:0042302 | structural constituent of cuticle |
| Lbot_4FC_C348 | 1.99 | 3.22E-05 | 0.0441 | apolipophorins isoform X1 | *Spodoptera litura* | GO:0005319;GO:0006869 | lipid transporter activity;lipid transport |
| Lbot_4FC_C3539 | 5.32 | 1.11E-05 | 0.0058 | ---NA--- |  |  |  |
| Lbot_4FC_C4648 | 8.84 | 8.08E-06 | 0.0315 | adhesive plaque matrix protein-like | *Bombyx mori* | GO:0016021;GO:0042302 | integral component of membrane;structural constituent of cuticle |
| Lbot_4FC_C32778 | 12.38 | 1.29E-05 | <0.0001 | ---NA--- |  |  |  |
| Lbot_4FC_C4681 | 15.07 | 1.18E-05 | <0.0001 | uncharacterized LOC106122463 precursor | *Spodoptera litura* | GO:0016021 | integral component of membrane |
| Lbot_4FC_C17829 | 26.82 | 1.21E-05 | <0.0001 | probable nuclear hormone receptor HR3 isoform X1 | *Galleria mellonella* | GO:0003707;GO:0004879;GO:0005634;GO:0006355;GO:0008270;GO:0030522;GO:0043401;GO:0043565 | steroid hormone receptor activity;nuclear receptor activity;nucleus;regulation of transcription, DNA-templated;zinc ion binding;intracellular receptor signaling pathway;steroid hormone mediated signaling pathway;sequence-specific DNA binding |
| Lbot_4FC_C23329 | 48.28 | 5.86E-06 | 0.0224 | uncharacterized protein LOC110993417 | *Pieris rapae* |  |  |
| Lbot_4FC_C91807 | 70.9 | 8.66E-06 | 0.0003 | uncharacterized protein LOC113519226 | *Galleria mellonella* |  |  |
| Lbot_4FC_C53688 | 94.15 | 1.15E-05 | <0.0001 | osiris 9F | *Vanessa tameamea* | GO:0016021 | integral component of membrane |
| Lbot_4FC_C57137 | 99.71 | 2.39E-05 | <0.0001 | urbain precursor | *Galleria mellonella* |  |  |

**Table S20:** Significantly differentially expressed contigs in *L. botrana* larvae feeding on véraison berries of ‘Riesling’ under ambient *vs.* elevated CO_2_ concentration (EdgeR-analysis). A negative fold change indicates that the expression levels were lower in larvae feeding on eCO_2_-plants.

| Feature ID | Fold change | Weighted difference | FDR p-value correction | Sequence Description | Blast Top Hit Taxonomy Name | Annotation GO ID | Annotation GO Term |
| --- | --- | --- | --- | --- | --- | --- | --- |
| Lbot_4FC_C86817 | -10.32 | -6.38E-05 | <0.0001 | acidic juvenile hormone-suppressible protein 1-like | *Plutella xylostella* |  |  |
| Lbot_4FC_C37067 | -8.37 | -6.71E-05 | <0.0001 | arylphorin subunit beta-like | *Leguminivora glycinivorella* | GO:0005615; GO:0045735 | extracellular space; nutrient reservoir activity |
| Lbot_4FC_C6445 | -7.73 | -8.35E-05 | <0.0001 | arylphorin precursor | *Leguminivora glycinivorella* | GO:0005615; GO:0045735 | extracellular space; nutrient reservoir activity |
| Lbot_4FC_C53688 | 88.54 | 1.08E-05 | <0.0001 | osiris 9F | *Vanessa tameamea* | GO:0016021 | integral component of membrane |
| Lbot_4FC_C85940 | 111.05 | 1.36E-05 | <0.0001 | osiris 9E | *Hyposmocoma kahamanoa* | GO:0016021 | integral component of membrane |

**Table S21:** Significantly differentially expressed contigs in *L. botrana* larvae feeding on inflorescences of ‘Cabernet Sauvignon’ under ambient *vs.* elevated CO_2_ concentration (EdgeR-analysis). A negative fold change indicates that the expression levels were lower in larvae feeding on eCO_2_-plants.

| Feature ID | Fold change | Weighted difference | FDR p-value correction | Sequence Description | Blast Top Hit Taxonomy Name | Annotation GO ID | Annotation GO Term |
| --- | --- | --- | --- | --- | --- | --- | --- |
| Lbot_4FC_C60369 | -49.25 | -1.10E-05 | <0.0001 | endocuticle structural glycoprotein SgAbd-5-like | *Trichoplusia ni* | GO:0042302 | structural constituent of cuticle |
| Lbot_4FC_C60020 | -43.71 | -1.85E-05 | <0.0001 | ---NA--- |  |  |  |
| Lbot_4FC_C60171 | -38.07 | -9.42E-06 | 0.0007 | cuticle protein 8-like | *Hyposmocoma kahamanoa* | GO:0042302 | structural constituent of cuticle |
| Lbot_4FC_C59918 | -37.4 | -1.85E-05 | <0.0001 | ecdysteroid regulated protein | *Vanessa tameamea* | GO:0016021 | integral component of membrane |
| Lbot_4FC_C60039 | -36.8 | -2.62E-05 | <0.0001 | ---NA--- |  |  |  |
| Lbot_4FC_C37067 | -34.6 | -8.47E-05 | <0.0001 | arylphorin subunit beta-like | *Leguminivora glycinivorella* | GO:0005615; GO:0045735 | extracellular space; nutrient reservoir activity |
| Lbot_4FC_C59726 | -26.56 | -1.67E-05 | <0.0001 | larval cuticle protein 8-like | *Galleria mellonella* | GO:0042302 | structural constituent of cuticle |
| Lbot_4FC_C17381 | -26.17 | -1.39E-05 | <0.0001 | methionine-rich storage protein | *Choristoneura fumiferana* |  |  |
| Lbot_4FC_C6445 | -19.84 | -1.27E-04 | <0.0001 | arylphorin precursor | *Leguminivora glycinivorella* | GO:0005615; GO:0045735 | extracellular space; nutrient reservoir activity |
| Lbot_4FC_C86817 | -17.68 | -2.91E-05 | 0.0001 | acidic juvenile hormone-suppressible protein 1-like | *Plutella xylostella* |  |  |
| Lbot_4FC_C59790 | -16.63 | -1.12E-05 | 0.0001 | glycine-rich cell wall structural protein | *Papilio machaon* |  |  |
| Lbot_4FC_C51784 | -14.03 | -9.47E-06 | 0.0015 | ---NA--- |  |  |  |
| Lbot_4FC_C37066 | -12.22 | -3.63E-05 | 0.0001 | arylphorin subunit beta-like | *Leguminivora glycinivorella* | GO:0005615; GO:0045735 | extracellular space; nutrient reservoir activity |
| Lbot_4FC_C11616 | -10.72 | -9.18E-06 | 0.0074 | trypsin CFT-1-like | *Galleria mellonella* | GO:0004252; GO:0005615; GO:0006508 | serine-type endopeptidase activity; extracellular space; proteolysis |
| Lbot_4FC_C41869 | -10.55 | -1.35E-05 | <0.0001 | endocuticle structural glycoprotein SgAbd-5-like | *Papilio machaon* | GO:0016021; GO:0042302 | integral component of membrane; structural constituent of cuticle |
| Lbot_4FC_C27656 | -9.36 | -2.84E-05 | <0.0001 | ---NA--- |  |  |  |
| Lbot_4FC_C1813 | -8.96 | -1.44E-05 | <0.0001 | uncharacterized protein LOC113508223 | *Trichoplusia ni* | GO:0003676; GO:0015074 | nucleic acid binding; DNA integration |
| Lbot_4FC_C36705 | -8.79 | -1.63E-05 | <0.0001 | endocuticle structural glycoprotein SgAbd-8-like | *Trichoplusia ni* | GO:0042302 | structural constituent of cuticle |
| Lbot_4FC_C60097 | -8.17 | -3.46E-05 | <0.0001 | endocuticle structural glycoprotein ABD-4-like | *Hyposmocoma kahamanoa* | GO:0016021; GO:0042302 | integral component of membrane; structural constituent of cuticle |
| Lbot_4FC_C27655 | -7.01 | -2.87E-05 | <0.0001 | ---NA--- |  |  |  |
| Lbot_4FC_C1369 | -5.23 | -6.21E-05 | <0.0001 | arylphorin precursor | *Leguminivora glycinivorella* | GO:0005615; GO:0045735 | extracellular space; nutrient reservoir activity |
| Lbot_4FC_C36854 | -4.88 | -1.49E-05 | 0.0008 | mediator of RNA polymerase II transcription subunit 15-like | *Amyelois transitella* |  |  |
| Lbot_4FC_C22063 | -4.85 | -1.26E-05 | 0.0078 | cuticle protein 3-like | *Galleria mellonella* | GO:0042302 | structural constituent of cuticle |
| Lbot_4FC_C60932 | -4.02 | -1.80E-05 | 0.0007 | larval cuticle protein 1-like | *Choristoneura fumiferana* | GO:0003677; GO:0005664; GO:0006260; GO:0042302 | DNA binding; nuclear origin of replication recognition complex; DNA replication; structural constituent of cuticle |
| Lbot_4FC_C1374 | -4 | -2.68E-05 | <0.0001 | ---NA--- |  |  |  |
| Lbot_4FC_C60285 | -3.57 | -3.29E-05 | <0.0001 | larval cuticle protein LCP-17-like | *Spodoptera litura* | GO:0042302 | structural constituent of cuticle |
| Lbot_4FC_C705 | -3.51 | -8.38E-05 | 0.0004 | arylphorin precursor | *Leguminivora glycinivorella* | GO:0005615; GO:0045735 | extracellular space; nutrient reservoir activity |
| Lbot_4FC_C20712 | -2.79 | -2.13E-05 | 0.0078 | larval cuticle protein LCP-17-like | *Hyposmocoma kahamanoa* | GO:0042302 | structural constituent of cuticle |
| Lbot_4FC_C270 | -2 | -4.74E-05 | 0.0049 | PREDICTED: uncharacterized protein LOC106709627 | *Papilio machaon* |  |  |
| Lbot_4FC_C22002 | 1.59 | 7.25E-04 | 0.0261 | Cuticular protein glycine-rich 20 | *Vanessa tameamea* | GO:0098542 | defense response to other organism |
| Lbot_4FC_C57137 | 25.65 | 6.34E-06 | 0.0117 | urbain precursor | *Galleria mellonella* |  |  |
| Lbot_4FC_C3539 | 93.98 | 1.53E-05 | <0.0001 | ---NA--- |  |  |  |

**Table S22:** Significantly differentially expressed contigs in *L. botrana* larvae feeding on véraison berries of ‘Cabernet Sauvignon’ under ambient *vs.* elevated CO_2_ concentration (EdgeR-analysis). A negative fold change indicates that the expression levels were lower in larvae feeding on eCO_2_-plants.

| Feature ID | Fold change | Weighted difference | FDR p-value correction | Sequence Description | Blast Top Hit Taxonomy Name | Annotation GO ID | Annotation GO Term |
| --- | --- | --- | --- | --- | --- | --- | --- |
| Lbot_4FC_C86817 | -332.19 | -4.90E-05 | <0.0001 | acidic juvenile hormone-suppressible protein 1-like | *Plutella xylostella* |  |  |
| Lbot_4FC_C61342 | -89.6 | -1.10E-05 | <0.0001 | ---NA--- |  |  |  |
| Lbot_4FC_C3539 | -60.2 | -7.33E-06 | 0.0015 | ---NA--- |  |  |  |
| Lbot_4FC_C37067 | -59.36 | -1.76E-05 | <0.0001 | arylphorin subunit beta-like | *Leguminivora glycinivorella* | GO:0005615; GO:0045735 | extracellular space; nutrient reservoir activity |
| Lbot_4FC_C17381 | -57.7 | -2.19E-05 | <0.0001 | methionine-rich storage protein | *Choristoneura fumiferana* |  |  |
| Lbot_4FC_C6445 | -55.63 | -2.16E-05 | <0.0001 | arylphorin precursor | *Leguminivora glycinivorella* | GO:0005615; GO:0045735 | extracellular space; nutrient reservoir activity |
| Lbot_4FC_C60370 | -52.08 | -1.03E-05 | <0.0001 | endocuticle structural glycoprotein SgAbd-5-like | *Helicoverpa armigera* | GO:0042302 | structural constituent of cuticle |
| Lbot_4FC_C30297 | -50.8 | -1.76E-05 | <0.0001 | basic juvenile hormone-suppressible protein 1-like | *Danaus plexippus plexippus* | GO:0005615; GO:0045735 | extracellular space; nutrient reservoir activity |
| Lbot_4FC_C60020 | -49.98 | -6.07E-06 | 0.0079 | ---NA--- |  |  |  |
| Lbot_4FC_C60369 | -47.37 | -9.45E-06 | <0.0001 | endocuticle structural glycoprotein SgAbd-5-like | *Trichoplusia ni* | GO:0042302 | structural constituent of cuticle |
| Lbot_4FC_C60039 | -40.09 | -6.49E-06 | 0.0044 | ---NA--- |  |  |  |
| Lbot_4FC_C42322 | -37.82 | -1.27E-05 | <0.0001 | basic juvenile hormone-suppressible protein 1-like | *Helicoverpa armigera* |  |  |
| Lbot_4FC_C59790 | -37.5 | -1.10E-05 | <0.0001 | glycine-rich cell wall structural protein | *Papilio machaon* |  |  |
| Lbot_4FC_C71661 | -29.73 | -1.17E-05 | <0.0001 | basic juvenile hormone-suppressible protein 1-like | *Heliconius erato* | GO:0005615; GO:0045735 | extracellular space; nutrient reservoir activity |
| Lbot_4FC_C59726 | -27.11 | -7.70E-06 | 0.0002 | larval cuticle protein 8-like | *Galleria mellonella* | GO:0042302 | structural constituent of cuticle |
| Lbot_4FC_C51784 | -20.41 | -6.19E-06 | 0.0044 | ---NA--- |  |  |  |
| Lbot_4FC_C60865 | -19.59 | -7.26E-06 | 0.0008 | ---NA--- |  |  |  |
| Lbot_4FC_C60353 | -19.57 | -6.43E-06 | 0.0026 | ---NA--- |  |  |  |
| Lbot_4FC_C36705 | -17.45 | -1.20E-05 | <0.0001 | endocuticle structural glycoprotein SgAbd-8-like | *Trichoplusia ni* | GO:0042302 | structural constituent of cuticle |
| Lbot_4FC_C41869 | -13.7 | -6.29E-06 | 0.024 | endocuticle structural glycoprotein SgAbd-5-like | *Papilio machaon* | GO:0016021; GO:0042302 | integral component of membrane; structural constituent of cuticle |
| Lbot_4FC_C60097 | -9.63 | -1.65E-05 | <0.0001 | endocuticle structural glycoprotein ABD-4-like | *Hyposmocoma kahamanoa* | GO:0016021; GO:0042302 | integral component of membrane; structural constituent of cuticle |
| Lbot_4FC_C42867 | -5.05 | -2.28E-05 | <0.0001 | probable Ras GTPase-activating protein isoform X6 | *Bicyclus anynana* | GO:0007165; GO:0043087 | signal transduction; regulation of GTPase activity |
| Lbot_4FC_C84525 | 18.34 | 6.51E-06 | 0.0044 | ---NA--- |  |  |  |
| Lbot_4FC_C57137 | 51.33 | 1.05E-05 | <0.0001 | urbain precursor | *Galleria mellonella* |  |  |

**Table S23:** Significantly differentially expressed contigs in *L. botrana* larvae feeding on inflorescences *vs.* véraison berries of ‘Riesling’ under ambient CO_2_ concentration (EdgeR-analysis). A negative fold change indicates that the expression levels were lower in larvae feeding on véraison berries.

| Feature ID | Fold change | Weighted difference | FDR p-value correction | Sequence Description | Blast Top Hit Taxonomy Name | Annotation GO ID | Annotation GO Term |
| --- | --- | --- | --- | --- | --- | --- | --- |
| Lbot_4FC_C60097 | -368.53 | -4.5514E-05 | <0.0001 | endocuticle structural glycoprotein ABD-4-like | *Hyposmocoma kahamanoa* | GO:0016021; GO:0042302 | integral component of membrane;structural constituent of cuticle |
| Lbot_4FC_C27656 | -225.22 | -2.7766E-05 | <0.0001 | ---NA--- |  |  |  |
| Lbot_4FC_C60039 | -188.13 | -2.3173E-05 | <0.0001 | ---NA--- |  |  |  |
| Lbot_4FC_C59918 | -114.69 | -1.4079E-05 | <0.0001 | ecdysteroid regulated protein | *Vanessa tameamea* | GO:0016021 | integral component of membrane |
| Lbot_4FC_C60020 | -82.60 | -1.0105E-05 | <0.0001 | ---NA--- |  |  |  |
| Lbot_4FC_C60369 | -78.92 | -9.6496E-06 | <0.0001 | endocuticle structural glycoprotein SgAbd-5-like | *Trichoplusia ni* | GO:0042302 | structural constituent of cuticle |
| Lbot_4FC_C60171 | -77.38 | -9.4587E-06 | <0.0001 | cuticle protein 8-like | *Hyposmocoma kahamanoa* | GO:0042302 | structural constituent of cuticle |
| Lbot_4FC_C59726 | -67.82 | -2.0128E-05 | <0.0001 | larval cuticle protein 8-like | *Galleria mellonella* | GO:0042302 | structural constituent of cuticle |
| Lbot_4FC_C36705 | -58.64 | -2.1049E-05 | <0.0001 | endocuticle structural glycoprotein SgAbd-8-like | *Trichoplusia ni* | GO:0042302 | structural constituent of cuticle |
| Lbot_4FC_C41869 | -54.59 | -1.3546E-05 | <0.0001 | endocuticle structural glycoprotein SgAbd-5-like | *Papilio machaon* | GO:0016021; GO:0042302 | integral component of membrane;structural constituent of cuticle |
| Lbot_4FC_C22063 | -53.84 | -1.4314E-05 | <0.0001 | cuticle protein 3-like | *Galleria mellonella* | GO:0042302 | structural constituent of cuticle |
| Lbot_4FC_C20712 | -53.23 | -3.5066E-05 | <0.0001 | larval cuticle protein LCP-17-like | *Hyposmocoma kahamanoa* | GO:0042302 | structural constituent of cuticle |
| Lbot_4FC_C60932 | -50.71 | -2.9558E-05 | <0.0001 | larval cuticle protein 1-like | *Choristoneura fumiferana* | GO:0003677; GO:0005664; GO:0006260; GO:0042302 | DNA binding;nuclear origin of replication recognition complex;DNA replication;structural constituent of cuticle |
| Lbot_4FC_C59790 | -48.95 | -1.3142E-05 | <0.0001 | glycine-rich cell wall structural protein | *Papilio machaon* |  |  |
| Lbot_4FC_C29817 | -48.44 | -1.3419E-05 | <0.0001 | ---NA--- |  |  |  |
| Lbot_4FC_C51784 | -46.94 | -7.7936E-06 | 0.0005 | ---NA--- |  |  |  |
| Lbot_4FC_C61212 | -46.44 | -6.4842E-06 | 0.0053 | ---NA--- |  |  |  |
| Lbot_4FC_C36854 | -44.21 | -2.1757E-05 | <0.0001 | mediator of RNA polymerase II transcription subunit 15-like | *Amyelois transitella* |  |  |
| Lbot_4FC_C48141 | -43.43 | -9.505E-06 | <0.0001 | uncharacterized protein LOC113512554 | *Galleria mellonella* |  |  |
| Lbot_4FC_C61822 | -42.45 | -5.1326E-06 | 0.0460 | TPA: putative cuticle protein | *Helicoverpa armigera* | GO:0042302 | structural constituent of cuticle |
| Lbot_4FC_C60285 | -38.39 | -4.866E-05 | <0.0001 | larval cuticle protein LCP-17-like | *Spodoptera litura* | GO:0042302 | structural constituent of cuticle |
| Lbot_4FC_C54056 | -35.18 | -6.0945E-06 | 0.0094 | pupal cuticle protein 36-like | *Galleria mellonella* | GO:0042302 | structural constituent of cuticle |
| Lbot_4FC_C59977 | -34.62 | -1.5627E-05 | <0.0001 | endocuticle structural glycoprotein SgAbd-2-like | *Papilio machaon* | GO:0042302 | structural constituent of cuticle |
| Lbot_4FC_C55973 | -33.82 | -8.5345E-06 | 0.0002 | pupal cuticle protein-like | *Plutella xylostella* |  |  |
| Lbot_4FC_C60353 | -32.03 | -6.3903E-06 | 0.0053 | ---NA--- |  |  |  |
| Lbot_4FC_C56678 | -30.39 | -6.5852E-06 | 0.0031 | golgin subfamily A member 6-like protein 22 | *Amyelois transitella* |  |  |
| Lbot_4FC_C53647 | -26.50 | -7.5583E-06 | 0.0050 | Cuticular protein PpolCPG14 | *Operophtera brumata* |  |  |
| Lbot_4FC_C59598 | -26.48 | -7.5794E-06 | 0.0050 | paternally-expressed gene 3 protein | *Papilio machaon* |  |  |
| Lbot_4FC_C44941 | -25.75 | -5.5137E-06 | 0.0150 | circadian clock-controlled protein-like | *Papilio polytes* |  |  |
| Lbot_4FC_C15311 | -25.68 | -1.1261E-05 | <0.0001 | cuticle protein 3 | *Amyelois transitella* | GO:0042302 | structural constituent of cuticle |
| Lbot_4FC_C30968 | -22.33 | -7.2014E-06 | 0.0084 | pupal cuticle protein-like | *Hyposmocoma kahamanoa* |  |  |
| Lbot_4FC_C38651 | -21.88 | -7.0991E-06 | 0.0131 | organic cation transporter protein-like | *Amyelois transitella* | GO:0016021; GO:0022857; GO:0055085 | integral component of membrane;transmembrane transporter activity;transmembrane transport |
| Lbot_4FC_C8166 | -21.64 | -1.5887E-05 | <0.0001 | flexible cuticle protein 12-like | *Plutella xylostella* | GO:0042302 | structural constituent of cuticle |
| Lbot_4FC_C51783 | -21.18 | -5.5766E-06 | 0.0150 | ---NA--- |  |  |  |
| Lbot_4FC_C13396 | -16.65 | -1.6964E-05 | <0.0001 | tyrosine hydroxylase | *Vanessa tameamea* | GO:0004511; GO:0005506; GO:0009072; GO:0042423; GO:0055114 | tyrosine 3-monooxygenase activity;iron ion binding;aromatic amino acid family metabolic process;catecholamine biosynthetic process;oxidation-reduction process |
| Lbot_4FC_C58155 | -16.58 | -6.8516E-06 | 0.0131 | cysteine sulfinic acid decarboxylase | *Galleria mellonella* | GO:0004068; GO:0004351; GO:0019752; GO:0030170 | aspartate 1-decarboxylase activity;glutamate decarboxylase activity;carboxylic acid metabolic process;pyridoxal phosphate binding |
| Lbot_4FC_C27655 | -15.14 | -2.5615E-05 | <0.0001 | ---NA--- |  |  |  |
| Lbot_4FC_C37629 | -14.96 | -8.4195E-06 | 0.0010 | pupal cuticle protein 36-like | *Vanessa tameamea* | GO:0042302 | structural constituent of cuticle |
| Lbot_4FC_C38267 | -14.90 | -1.1462E-05 | <0.0001 | uncharacterized LOC106124634 precursor | *Trichoplusia ni* |  |  |
| Lbot_4FC_C57559 | -14.58 | -1.4121E-05 | <0.0001 | tyrosine hydroxylase | *Heliothis virescens* | GO:0004511; GO:0005506; GO:0009072; GO:0042423; GO:0055114 | tyrosine 3-monooxygenase activity;iron ion binding;aromatic amino acid family metabolic process;catecholamine biosynthetic process;oxidation-reduction process |
| Lbot_4FC_C36971 | -13.98 | -1.2205E-05 | 0.0001 | ---NA--- |  |  |  |
| Lbot_4FC_C20745 | -13.56 | -6.7663E-06 | 0.0131 | mediator of DNA damage checkpoint protein 1-like isoform X3 | *Bombyx mori* | GO:0016021 | integral component of membrane |
| Lbot_4FC_C34282 | -13.41 | -1.2302E-05 | <0.0001 | cuticle protein 16.5-like | *Galleria mellonella* |  |  |
| Lbot_4FC_C12628 | -12.50 | -1.7681E-05 | <0.0001 | cell wall protein RBR3-like | *Amyelois transitella* | GO:0042302 | structural constituent of cuticle |
| Lbot_4FC_C49758 | -10.64 | -9.4697E-06 | 0.0021 | uncharacterized LOC106128368 precursor | *Amyelois transitella* | GO:0016021 | integral component of membrane |
| Lbot_4FC_C11631 | -8.83 | -1.5488E-05 | <0.0001 | putative defense protein Hdd11 | *Heliothis virescens* | GO:0005576; GO:0042742; GO:0042832; GO:0045087 | extracellular region;defense response to bacterium;defense response to protozoan;innate immune response |
| Lbot_4FC_C43016 | -8.11 | -8.5598E-06 | 0.0053 | ---NA--- |  |  |  |
| Lbot_4FC_C39036 | -7.70 | -8.2681E-06 | 0.0084 | cuticle protein 8-like | *Amyelois transitella* | GO:0042302 | structural constituent of cuticle |
| Lbot_4FC_C26308 | -6.59 | -8.7227E-06 | 0.0111 | pancreatic triacylglycerol lipase-like | *Mamestra configurata* | GO:0005576; GO:0006629; GO:0052689 | extracellular region;lipid metabolic process;carboxylic ester hydrolase activity |
| Lbot_4FC_C11400 | -6.29 | -7.9898E-06 | 0.0248 | protein obstructor-E isoform X1 | *Galleria mellonella* | GO:0005576; GO:0006030; GO:0008061 | extracellular region;chitin metabolic process;chitin binding |
| Lbot_4FC_C883 | -5.99 | -3.3187E-05 | <0.0001 | pancreatic triacylglycerol lipase-like | *Bombyx mori* | GO:0005576; GO:0006629; GO:0052689 | extracellular region;lipid metabolic process;carboxylic ester hydrolase activity |
| Lbot_4FC_C705 | -5.91 | -3.0562E-05 | <0.0001 | arylphorin precursor | *Leguminivora glycinivorella* | GO:0005615; GO:0045735 | extracellular space;nutrient reservoir activity |
| Lbot_4FC_C1475 | -5.16 | -1.5405E-05 | 0.0003 | C-type lectin 27kD | *Vanessa tameamea* | GO:0030246 | carbohydrate binding |
| Lbot_4FC_C14030 | -4.47 | -1.3162E-05 | 0.0027 | ejaculatory bulb-specific protein 3-like isoform X2 | *Lobesia botrana* |  |  |
| Lbot_4FC_C3370 | -4.45 | -1.4118E-05 | 0.0013 | uncharacterized oxidoreductase YrbE-like | *Galleria mellonella* | GO:0016491; GO:0055114 | oxidoreductase activity;oxidation-reduction process |
| Lbot_4FC_C9736 | -4.40 | -1.3908E-05 | 0.0009 | cuticle protein 1-like | *Papilio xuthus* | GO:0005576; GO:0016021 | extracellular region;integral component of membrane |
| Lbot_4FC_C29756 | -4.17 | -1.0131E-05 | 0.0276 | ---NA--- |  |  |  |
| Lbot_4FC_C38667 | -4.14 | -1.4626E-05 | 0.0031 | ---NA--- |  |  |  |
| Lbot_4FC_C882 | -3.75 | -0.00011195 | <0.0001 | pancreatic triacylglycerol lipase-like | *Operophtera brumata* | GO:0005576; GO:0006629; GO:0052689 | extracellular region;lipid metabolic process;carboxylic ester hydrolase activity |
| Lbot_4FC_C1065 | -3.70 | -1.1162E-05 | 0.0203 | sucrose-6-phosphate hydrolase-like | *Galleria mellonella* | GO:0004564; GO:0005737; GO:0005975 | beta-fructofuranosidase activity;cytoplasm;carbohydrate metabolic process |
| Lbot_4FC_C63 | -3.53 | -5.357E-05 | <0.0001 | pancreatic triacylglycerol lipase-like | *Spodoptera litura* | GO:0005576; GO:0006629; GO:0052689 | extracellular region;lipid metabolic process;carboxylic ester hydrolase activity |
| Lbot_4FC_C60087 | -3.35 | -1.267E-05 | 0.0273 | hypothetical protein B5X24_HaOG206415 | *Helicoverpa armigera* |  |  |
| Lbot_4FC_C5107 | -3.34 | -7.541E-05 | <0.0001 | endocuticle structural glycoprotein ABD-5-like | *Pieris rapae* | GO:0042302 | structural constituent of cuticle |
| Lbot_4FC_C3183 | -3.13 | -1.4386E-05 | 0.0136 | fatty acid-binding protein, liver-like | *Plutella xylostella* |  |  |
| Lbot_4FC_C270 | -3.06 | -2.6115E-05 | 0.0001 | PREDICTED: uncharacterized protein LOC106709627 | *Papilio machaon* |  |  |
| Lbot_4FC_C850 | -3.01 | -1.6741E-05 | 0.0094 | ---NA--- |  |  |  |
| Lbot_4FC_C2121 | -2.94 | -1.5771E-05 | 0.0124 | chondroitin proteoglycan 2-like | *Spodoptera exigua* | GO:0005576; GO:0006030; GO:0008061 | extracellular region;chitin metabolic process;chitin binding |
| Lbot_4FC_C3345 | -2.81 | -2.5809E-05 | 0.0004 | esterase FE4-like | *Cydia pomonella* | GO:0016787 | hydrolase activity |
| Lbot_4FC_C145 | -2.54 | -1.9091E-05 | 0.0131 | carboxypeptidase B-like | *Papilio xuthus* | GO:0004181; GO:0004930; GO:0006508; GO:0007186; GO:0008270; GO:0016021 | metallocarboxypeptidase activity;G protein-coupled receptor activity;proteolysis;G protein-coupled receptor signaling pathway;zinc ion binding;integral component of membrane |
| Lbot_4FC_C4427 | -2.37 | -3.1574E-05 | 0.0013 | larval cuticle protein LCP-17-like | *Papilio polytes* | GO:0042302 | structural constituent of cuticle |
| Lbot_4FC_C826 | -2.20 | -2.9989E-05 | 0.0056 | pancreatic triacylglycerol lipase-like | *Antheraea pernyi* | GO:0005576; GO:0006629; GO:0052689 | extracellular region;lipid metabolic process;carboxylic ester hydrolase activity |
| Lbot_4FC_C55 | -2.14 | -6.9594E-05 | <0.0001 | flexible cuticle protein 12-like | *Nicrophorus vespilloides* | GO:0042302 | structural constituent of cuticle |
| Lbot_4FC_C2691 | -2.00 | -2.6191E-05 | 0.0361 | pancreatic triacylglycerol lipase-like | *Antheraea yamamai* | GO:0005576; GO:0006629; GO:0052689 | extracellular region;lipid metabolic process;carboxylic ester hydrolase activity |
| Lbot_4FC_C1721 | -1.98 | -3.5037E-05 | 0.0119 | fatty acid-binding protein 2-like | *Papilio machaon* | GO:0005737; GO:0008289 | cytoplasm;lipid binding |
| Lbot_4FC_C25179 | -1.81 | -0.0049439 | <0.0001 | predicted protein | *Nematostella vectensis* | 0 | 0 |
| Lbot_4FC_C278 | -1.79 | -4.6258E-05 | 0.0201 | trypsin CFT-1-like | *Choristoneura fumiferana* | GO:0004252; GO:0005615; GO:0006508 | serine-type endopeptidase activity;extracellular space;proteolysis |
| Lbot_4FC_C151 | -1.77 | -0.00013815 | 0.0013 | chitin-binding protein | *Tribolium castaneum* |  |  |
| Lbot_4FC_C22692 | 1.55 | 0.01827679 | 0.0201 | predicted protein | *Atta colombica* |  |  |
| Lbot_4FC_C22002 | 1.57 | 0.00077612 | 0.0150 | Cuticular protein glycine-rich 20 | *Vanessa tameamea* | GO:0098542 | defense response to other organism |
| Lbot_4FC_C13362 | 1.74 | 5.0545E-05 | 0.0346 | ---NA--- |  |  |  |
| Lbot_4FC_C9606 | 1.89 | 4.6214E-05 | 0.0100 | ---NA--- |  |  |  |
| Lbot_4FC_C5045 | 1.96 | 3.0631E-05 | 0.0280 | probable cyclin-dependent serine/threonine-protein kinase DDB_G0292550 isoform X1 | *Galleria mellonella* |  |  |
| Lbot_4FC_C2175 | 2.02 | 2.6847E-05 | 0.0361 | ---NA--- |  |  |  |
| Lbot_4FC_C7358 | 2.05 | 2.497E-05 | 0.0320 | fatty acid synthase | *Bombyx mori* | GO:0004312; GO:0009058; GO:0016491; GO:0016788; GO:0031177; GO:0055114 | fatty acid synthase activity;biosynthetic process;oxidoreductase activity;hydrolase activity, acting on ester bonds;phosphopantetheine binding;oxidation-reduction process |
| Lbot_4FC_C2434 | 2.06 | 3.7616E-05 | 0.0047 | odorant binding protein 1 | *Lobesia botrana* | GO:0005549 | odorant binding |
| Lbot_4FC_C70 | 2.18 | 0.00014468 | <0.0001 | cytochrome P450 4g15 | *Plutella xylostella* | GO:0004497; GO:0005506; GO:0016021; GO:0016705; GO:0020037; GO:0055114 | monooxygenase activity;iron ion binding;integral component of membrane;oxidoreductase activity, acting on paired donors, with incorporation or reduction of molecular oxygen;heme binding;oxidation-reduction process |
| Lbot_4FC_C4103 | 2.79 | 1.7803E-05 | 0.0097 | vanin-like protein 2 isoform X1 | *Papilio machaon* | GO:0006807 | nitrogen compound metabolic process |
| Lbot_4FC_C2221 | 3.05 | 1.4327E-05 | 0.0168 | diapausin precursor | *Spodoptera exigua* | GO:0005576; GO:0031640; GO:0042742; GO:0045087; GO:0050832 | extracellular region;killing of cells of other organism;defense response to bacterium;innate immune response;defense response to fungus |
| Lbot_4FC_C6445 | 6.18 | 8.0401E-05 | 0.0000 | arylphorin precursor | *Leguminivora glycinivorella* | GO:0005615; GO:0045735 | extracellular space;nutrient reservoir activity |
| Lbot_4FC_C12738 | 7.97 | 7.3362E-06 | 0.0303 | hypothetical protein P168DRAFT_121299 | *Aspergillus campestris IBT 28561* |  |  |
| Lbot_4FC_C27792 | 14.79 | 6.7653E-06 | 0.0131 | ---NA--- |  |  |  |
| Lbot_4FC_C37067 | 19.18 | 7.2177E-05 | <0.0001 | arylphorin subunit beta-like | *Leguminivora glycinivorella* | GO:0005615; GO:0045735 | extracellular space;nutrient reservoir activity |
| Lbot_4FC_C86817 | 20.66 | 6.7221E-05 | <0.0001 | acidic juvenile hormone-suppressible protein 1-like | *Plutella xylostella* |  |  |
| Lbot_4FC_C77814 | 21.43 | 5.4143E-06 | 0.0150 | ---NA--- |  |  |  |

**Table S24:** Significantly differentially expressed contigs in *L. botrana* larvae feeding on inflorescences *vs.* véraison berries of ‘Riesling’ under elevated CO_2_ concentration (EdgeR-analysis). A negative fold change indicates that the expression levels were lower in larvae feeding on véraison berries.

| Feature ID | Fold change | Weighted difference | FDR p-value correction | Sequence Description | Blast Top Hit Taxonomy Name | Annotation GO ID | Annotation GO Term |
| --- | --- | --- | --- | --- | --- | --- | --- |
| Lbot_4FC_C60039 | -55.91 | -1.19E-05 | <0.0001 | ---NA--- |  |  |  |
| Lbot_4FC_C60020 | -53.87 | -7.59E-06 | 0.0007 | ---NA--- |  |  |  |
| Lbot_4FC_C27656 | -48.54 | -1.18E-05 | <0.0001 | ---NA--- |  |  |  |
| Lbot_4FC_C31065 | -42.58 | -5.15E-06 | 0.0361 | ---NA--- |  |  |  |
| Lbot_4FC_C59918 | -35.12 | -5.79E-06 | 0.012 | ecdysteroid regulated protein | *Vanessa tameamea* | GO:0016021 | integral component of membrane |
| Lbot_4FC_C60097 | -30.95 | -2.10E-05 | <0.0001 | endocuticle structural glycoprotein ABD-4-like | *Hyposmocoma kahamanoa* | GO:0016021; GO:0042302 | integral component of membrane; structural constituent of cuticle |
| Lbot_4FC_C60369 | -28.46 | -6.63E-06 | 0.0037 | endocuticle structural glycoprotein SgAbd-5-like | *Trichoplusia ni* | GO:0042302 | structural constituent of cuticle |
| Lbot_4FC_C4028 | -27.49 | -8.77E-06 | 0.0001 | Myrosinase 1 | *Papilio polytes* | GO:0004553; GO:0005975 | hydrolase activity, hydrolyzing O-glycosyl compounds; carbohydrate metabolic process |
| Lbot_4FC_C59726 | -27.15 | -1.21E-05 | <0.0001 | larval cuticle protein 8-like | *Galleria mellonella* | GO:0042302 | structural constituent of cuticle |
| Lbot_4FC_C36705 | -23.87 | -8.13E-06 | 0.0002 | endocuticle structural glycoprotein SgAbd-8-like | *Trichoplusia ni* | GO:0042302 | structural constituent of cuticle |
| Lbot_4FC_C59790 | -14.97 | -6.59E-06 | 0.0021 | glycine-rich cell wall structural protein | *Papilio machaon* |  |  |
| Lbot_4FC_C27655 | -13.23 | -1.07E-05 | <0.0001 | ---NA--- |  |  |  |
| Lbot_4FC_C32778 | -11.61 | -1.29E-05 | <0.0001 | ---NA--- |  |  |  |
| Lbot_4FC_C173 | -9.86 | -1.22E-05 | <0.0001 | myrosinase 1-like | *Papilio polytes* | GO:0004553; GO:0005975 | hydrolase activity, hydrolyzing O-glycosyl compounds; carbohydrate metabolic process |
| Lbot_4FC_C883 | -8.77 | -3.86E-05 | <0.0001 | pancreatic triacylglycerol lipase-like | *Bombyx mori* | GO:0005576; GO:0006629; GO:0052689 | extracellular region; lipid metabolic process; carboxylic ester hydrolase activity |
| Lbot_4FC_C60932 | -7.1 | -7.37E-06 | 0.0269 | larval cuticle protein 1-like | *Choristoneura fumiferana* | GO:0003677; GO:0005664; GO:0006260; GO:0042302 | DNA binding; nuclear origin of replication recognition complex; DNA replication; structural constituent of cuticle |
| Lbot_4FC_C2121 | -6.34 | -1.96E-05 | <0.0001 | chondroitin proteoglycan 2-like | *Spodoptera exigua* | GO:0005576; GO:0006030; GO:0008061 | extracellular region; chitin metabolic process; chitin binding |
| Lbot_4FC_C1058 | -6.03 | -3.01E-05 | <0.0001 | epidermal retinol dehydrogenase 2-like | *Vanessa tameamea* | GO:0016021; GO:0016491; GO:0055114 | integral component of membrane; oxidoreductase activity; oxidation-reduction process |
| Lbot_4FC_C3370 | -5.85 | -2.21E-05 | <0.0001 | uncharacterized oxidoreductase YrbE-like | *Galleria mellonella* | GO:0016491; GO:0055114 | oxidoreductase activity; oxidation-reduction process |
| Lbot_4FC_C2607 | -5.29 | -1.12E-05 | 0.001 | juvenile hormone esterase-like | *Cydia pomonella* | GO:0016787 | hydrolase activity |
| Lbot_4FC_C4648 | -5.23 | -7.37E-06 | 0.0495 | adhesive plaque matrix protein-like | *Bombyx mori* | GO:0016021; GO:0042302 | integral component of membrane; structural constituent of cuticle |
| Lbot_4FC_C1968 | -4.93 | -1.28E-05 | 0.0005 | Endonuclease G, mitochondrial | *Spodoptera litura* | GO:0003676; GO:0004519; GO:0046872; GO:0090305 | nucleic acid binding; endonuclease activity; metal ion binding; nucleic acid phosphodiester bond hydrolysis |
| Lbot_4FC_C705 | -4.92 | -2.29E-05 | <0.0001 | arylphorin precursor | *Leguminivora glycinivorella* | GO:0005615; GO:0045735 | extracellular space; nutrient reservoir activity |
| Lbot_4FC_C60285 | -4.73 | -1.35E-05 | 0.0022 | larval cuticle protein LCP-17-like | *Spodoptera litura* | GO:0042302 | structural constituent of cuticle |
| Lbot_4FC_C2644 | -4.2 | -1.42E-05 | 0.0008 | cytochrome P450 6B6-like | *Hyposmocoma kahamanoa* | GO:0004497; GO:0005506; GO:0016705; GO:0020037; GO:0055114 | monooxygenase activity; iron ion binding; oxidoreductase activity, acting on paired donors, with incorporation or reduction of molecular oxygen; heme binding; oxidation-reduction process |
| Lbot_4FC_C17829 | -4.11 | -9.53E-06 | 0.0361 | probable nuclear hormone receptor HR3 isoform X1 | *Galleria mellonella* | GO:0003707; GO:0004879; GO:0005634; GO:0006355; GO:0008270; GO:0030522; GO:0043401; GO:0043565 | steroid hormone receptor activity; nuclear receptor activity; nucleus; regulation of transcription, DNA-templated; zinc ion binding; intracellular receptor signaling pathway; steroid hormone mediated signaling pathway; sequence-specific DNA binding |
| Lbot_4FC_C145 | -4.09 | -3.34E-05 | <0.0001 | carboxypeptidase B-like | *Papilio xuthus* | GO:0004181; GO:0004930; GO:0006508; GO:0007186; GO:0008270; GO:0016021 | metallocarboxypeptidase activity; G protein-coupled receptor activity;proteolysis; G protein-coupled receptor signaling pathway; zinc ion binding; integral component of membrane |
| Lbot_4FC_C63 | -3.78 | -8.37E-05 | <0.0001 | pancreatic triacylglycerol lipase-like | *Spodoptera litura* | GO:0005576; GO:0006629; GO:0052689 | extracellular region; lipid metabolic process; carboxylic ester hydrolase activity |
| Lbot_4FC_C57137 | -3.73 | -1.77E-05 | 0.0002 | urbain precursor | *Galleria mellonella* | 0 | 0 |
| Lbot_4FC_C4027 | -3.7 | -9.96E-06 | 0.0379 | juvenile hormone epoxide hydrolase-like | *Helicoverpa armigera* | GO:0005789; GO:0016021; GO:0019439; GO:0033961 | endoplasmic reticulum membrane; integral component of membrane; aromatic compound catabolic process; cis-stilbene-oxide hydrolase activity |
| Lbot_4FC_C882 | -3.33 | -1.26E-04 | <0.0001 | pancreatic triacylglycerol lipase-like | *Operophtera brumata* | GO:0005576; GO:0006629; GO:0052689 | extracellular region; lipid metabolic process; carboxylic ester hydrolase activity |
| Lbot_4FC_C3035 | -3.28 | -2.02E-05 | 0.0002 | beta-1,3-glucan-binding protein-like | *Vanessa tameamea* | GO:0004553; GO:0005975 | hydrolase activity, hydrolyzing O-glycosyl compounds; carbohydrate metabolic process |
| Lbot_4FC_C4483 | -3.19 | -3.91E-05 | <0.0001 | fatty acid-binding protein, liver-like | *Plutella xylostella* |  |  |
| Lbot_4FC_C270 | -3.12 | -2.36E-05 | 0.0002 | PREDICTED: uncharacterized protein LOC106709627 | *Papilio machaon* |  |  |
| Lbot_4FC_C8460 | -3 | -1.17E-05 | 0.0368 | glutathione S-transferase-like | *Choristoneura fumiferana* | GO:0004364; GO:0005515 | glutathione transferase activity; protein binding |
| Lbot_4FC_C2954 | -2.85 | -1.22E-05 | 0.0445 | 3-ketoacyl-CoA thiolase, mitochondrial-like | *Galleria mellonella* | GO:0016747 | transferase activity, transferring acyl groups other than amino-acyl groups |
| Lbot_4FC_C3183 | -2.83 | -1.49E-05 | 0.0273 | fatty acid-binding protein, liver-like | *Plutella xylostella* |  |  |
| Lbot_4FC_C3173 | -2.71 | -2.49E-05 | 0.0004 | alpha-amylase 1-like | *Amyelois transitella* | GO:0004556; GO:0005975; GO:0043169; GO:0103025 | alpha-amylase activity; carbohydrate metabolic process; cation binding; alpha-amylase activity (releasing maltohexaose) |
| Lbot_4FC_C3345 | -2.7 | -2.78E-05 | 0.0001 | esterase FE4-like | *Cydia pomonella* | GO:0016787 | hydrolase activity |
| Lbot_4FC_C5213 | -2.69 | -1.75E-05 | 0.006 | uncharacterized family 31 glucosidase KIAA1161-like | *Bombyx mori* | GO:0004553; GO:0005975 | hydrolase activity, hydrolyzing O-glycosyl compounds; carbohydrate metabolic process |
| Lbot_4FC_C497 | -2.61 | -1.40E-05 | 0.0361 | retinal dehydrogenase 1-like | *Papilio xuthus* | GO:0016620; GO:0055114 | oxidoreductase activity, acting on the aldehyde or oxo group of donors, NAD or NADP as acceptor; oxidation-reduction process |
| Lbot_4FC_C1721 | -2.53 | -4.63E-05 | <0.0001 | fatty acid-binding protein 2-like | *Papilio machaon* | GO:0005737; GO:0008289 | cytoplasm; lipid binding |
| Lbot_4FC_C2437 | -2.36 | -3.02E-05 | 0.0008 | pancreatic triacylglycerol lipase-like | *Heliothis virescens* | GO:0005576; GO:0006629; GO:0052689 | extracellular region; lipid metabolic process; carboxylic ester hydrolase activity |
| Lbot_4FC_C278 | -2.32 | -7.47E-05 | <0.0001 | trypsin CFT-1-like | *Choristoneura fumiferana* | GO:0004252; GO:0005615; GO:0006508 | serine-type endopeptidase activity; extracellular space; proteolysis |
| Lbot_4FC_C2735 | -2.24 | -2.13E-05 | 0.0194 | probable medium-chain specific acyl-CoA dehydrogenase, mitochondrial isoform X1 | *Plutella xylostella* | GO:0003995; GO:0050660; GO:0055114 | acyl-CoA dehydrogenase activity; flavin adenine dinucleotide binding; oxidation-reduction process |
| Lbot_4FC_C2691 | -2.02 | -3.57E-05 | 0.005 | pancreatic triacylglycerol lipase-like | *Antheraea yamamai* | GO:0005576; GO:0006629; GO:0052689 | extracellular region; lipid metabolic process; carboxylic ester hydrolase activity |
| Lbot_4FC_C605 | -1.99 | -5.64E-05 | 0.0008 | pancreatic triacylglycerol lipase-like | *Papilio xuthus* | GO:0005576; GO:0052689 | extracellular region; carboxylic ester hydrolase activity |
| Lbot_4FC_C425 | -1.87 | -7.21E-05 | 0.0015 | zinc metalloproteinase nas-4-like | *Amyelois transitella* | GO:0004222; GO:0006508; GO:0008270 | metalloendopeptidase activity; proteolysis; zinc ion binding |
| Lbot_4FC_C808 | -1.78 | -6.53E-05 | 0.0101 | chitin deacetylase 1 | *Galleria mellonella* | GO:0004099; GO:0005975; GO:0051060 | chitin deacetylase activity; carbohydrate metabolic process; pullulanase activity |
| Lbot_4FC_C478 | -1.67 | -6.03E-05 | 0.0446 | carboxypeptidase B-like | *Helicoverpa armigera* | GO:0004181; GO:0006508; GO:0008270 | metallocarboxypeptidase activity; proteolysis; zinc ion binding |
| Lbot_4FC_C85 | -1.67 | -1.69E-04 | 0.0072 | ---NA--- |  |  |  |
| Lbot_4FC_C151 | -1.66 | -1.44E-04 | 0.0118 | chitin-binding protein | *Tribolium castaneum* |  |  |
| Lbot_4FC_C22692 | 1.53 | 1.47E-02 | 0.0377 | predicted protein | *Atta colombica* |  |  |
| Lbot_4FC_C150 | 1.79 | 3.76E-05 | 0.0442 | ---NA--- |  |  |  |
| Lbot_4FC_C9584 | 1.79 | 3.82E-05 | 0.0404 | ---NA--- |  |  |  |
| Lbot_4FC_C13362 | 1.81 | 4.52E-05 | 0.0173 | ---NA--- |  |  |  |
| Lbot_4FC_C70 | 1.82 | 1.24E-04 | 0.0007 | cytochrome P450 4g15 | *Plutella xylostella* | GO:0004497; GO:0005506; GO:0016021; GO:0016705; GO:0020037; GO:0055114 | monooxygenase activity; iron ion binding; integral component of membrane; oxidoreductase activity, acting on paired donors, with incorporation or reduction of molecular oxygen; heme binding; oxidation-reduction process |
| Lbot_4FC_C26723 | 2.04 | 4.25E-05 | 0.0016 | ---NA--- |  |  |  |
| Lbot_4FC_C2175 | 2.06 | 2.71E-05 | 0.0137 | ---NA--- |  |  |  |
| Lbot_4FC_C1374 | 2.63 | 2.14E-05 | 0.0024 | ---NA--- |  |  |  |
| Lbot_4FC_C1680 | 2.72 | 1.44E-05 | 0.0206 | fatty acid synthase | *Helicoverpa assulta* | GO:0004312; GO:0009058; GO:0016491; GO:0016788; GO:0031177; GO:0055114 | fatty acid synthase activity; biosynthetic process; oxidoreductase activity; hydrolase activity, acting on ester bonds; phosphopantetheine binding; oxidation-reduction process |
| Lbot_4FC_C7358 | 3.24 | 2.97E-05 | <0.0001 | fatty acid synthase | *Bombyx mori* | GO:0004312; GO:0009058; GO:0016491; GO:0016788; GO:0031177; GO:0055114 | fatty acid synthase activity; biosynthetic process; oxidoreductase activity; hydrolase activity, acting on ester bonds; phosphopantetheine binding; oxidation-reduction process |
| Lbot_4FC_C10605 | 3.58 | 1.06E-05 | 0.0382 | ---NA--- |  |  |  |
| Lbot_4FC_C37067 | 23.95 | 8.72E-06 | 0.0002 | arylphorin subunit beta-like | *Leguminivora glycinivorella* | GO:0005615; GO:0045735 | extracellular space; nutrient reservoir activity |
| Lbot_4FC_C6445 | 30.58 | 1.20E-05 | <0.0001 | arylphorin precursor | *Leguminivora glycinivorella* | GO:0005615; GO:0045735 | extracellular space; nutrient reservoir activity |
| Lbot_4FC_C86817 | 44.73 | 6.69E-06 | 0.0007 | acidic juvenile hormone-suppressible protein 1-like | *Plutella xylostella* |  |  |

**Table S25:** Significantly differentially expressed contigs in *L. botrana* larvae feeding on inflorescences *vs.* véraison berries of ‘Cabernet Sauvignon’ under ambient CO_2_ concentration (EdgeR-analysis). A negative fold change indicates that the expression levels were lower in larvae feeding on véraison berries.

| Feature ID | Fold change | Weighted difference | FDR p-value correction | Sequence Description | Blast Top Hit Taxonomy Name | Annotation GO ID | Annotation GO Term |
| --- | --- | --- | --- | --- | --- | --- | --- |
| Lbot_4FC_C59918 | -42.48 | -1.85E-05 | <0.0001 | ecdysteroid regulated protein | *Vanessa tameamea* | GO:0016021 | integral component of membrane |
| Lbot_4FC_C4028 | -28.62 | -9.95E-06 | <0.0001 | Myrosinase 1 | *Papilio polytes* | GO:0004553; GO:0005975 | hydrolase activity, hydrolyzing O-glycosyl compounds;carbohydrate metabolic process |
| Lbot_4FC_C37066 | -27.14 | -3.81E-05 | <0.0001 | arylphorin subunit beta-like | *Leguminivora glycinivorella* | GO:0005615; GO:0045735 | extracellular space;nutrient reservoir activity |
| Lbot_4FC_C11616 | -23.13 | -9.68E-06 | 0.0001 | trypsin CFT-1-like | *Galleria mellonella* | GO:0004252; GO:0005615; GO:0006508 | serine-type endopeptidase activity;extracellular space;proteolysis |
| Lbot_4FC_C173 | -15.55 | -1.71E-05 | <0.0001 | myrosinase 1-like | *Papilio polytes* | GO:0004553; GO:0005975 | hydrolase activity, hydrolyzing O-glycosyl compounds;carbohydrate metabolic process |
| Lbot_4FC_C1369 | -15.11 | -7.17E-05 | <0.0001 | arylphorin precursor | *Leguminivora glycinivorella* | GO:0005615; GO:0045735 | extracellular space;nutrient reservoir activity |
| Lbot_4FC_C705 | -13.81 | -0.00010875 | <0.0001 | arylphorin precursor | *Leguminivora glycinivorella* | GO:0005615; GO:0045735 | extracellular space;nutrient reservoir activity |
| Lbot_4FC_C883 | -13.75 | -4.79E-05 | <0.0001 | pancreatic triacylglycerol lipase-like | *Bombyx mori* | GO:0005576; GO:0006629; GO:0052689 | extracellular region;lipid metabolic process;carboxylic ester hydrolase activity |
| Lbot_4FC_C1728 | -13.23 | -6.99E-06 | 0.0048 | myrosinase 1-like | *Bombyx mori* | GO:0004553; GO:0005975; GO:0016021 | hydrolase activity, hydrolyzing O-glycosyl compounds;carbohydrate metabolic process;integral component of membrane |
| Lbot_4FC_C1727 | -9.72 | -6.29E-06 | 0.0139 | myrosinase 1-like | *Bombyx mori* | GO:0004553; GO:0005975; GO:0016021 | hydrolase activity, hydrolyzing O-glycosyl compounds;carbohydrate metabolic process;integral component of membrane |
| Lbot_4FC_C26308 | -8.81 | -7.69E-06 | 0.0052 | pancreatic triacylglycerol lipase-like | *Mamestra configurata* | GO:0005576; GO:0006629; GO:0052689 | extracellular region;lipid metabolic process;carboxylic ester hydrolase activity |
| Lbot_4FC_C36854 | -7.78 | -1.63E-05 | <0.0001 | mediator of RNA polymerase II transcription subunit 15-like | *Amyelois transitella* |  |  |
| Lbot_4FC_C13396 | -7.36 | -9.45E-06 | 0.0045 | tyrosine hydroxylase | *Vanessa tameamea* | GO:0004511; GO:0005506; GO:0009072; GO:0042423; GO:0055114 | tyrosine 3-monooxygenase activity;iron ion binding;aromatic amino acid family metabolic process;catecholamine biosynthetic process;oxidation-reduction process |
| Lbot_4FC_C270 | -6.85 | -8.10E-05 | <0.0001 | PREDICTED: uncharacterized protein LOC106709627 | *Papilio machaon* |  |  |
| Lbot_4FC_C57559 | -6.74 | -7.67E-06 | 0.0212 | tyrosine hydroxylase | *Heliothis virescens* | GO:0004511; GO:0005506; GO:0009072; GO:0042423; GO:0055114 | tyrosine 3-monooxygenase activity;iron ion binding;aromatic amino acid family metabolic process;catecholamine biosynthetic process;oxidation-reduction process |
| Lbot_4FC_C27656 | -6.56 | -2.69E-05 | <0.0001 | ---NA--- |  |  |  |
| Lbot_4FC_C882 | -6.15 | -0.00015037 | <0.0001 | pancreatic triacylglycerol lipase-like | *Operophtera brumata* | GO:0005576; GO:0006629; GO:0052689 | extracellular region;lipid metabolic process;carboxylic ester hydrolase activity |
| Lbot_4FC_C6445 | -6.05 | -0.00011119 | <0.0001 | arylphorin precursor | *Leguminivora glycinivorella* | GO:0005615; GO:0045735 | extracellular space;nutrient reservoir activity |
| Lbot_4FC_C63 | -5.91 | -9.87E-05 | <0.0001 | pancreatic triacylglycerol lipase-like | *Spodoptera litura* | GO:0005576; GO:0006629; GO:0052689 | extracellular region;lipid metabolic process;carboxylic ester hydrolase activity |
| Lbot_4FC_C27655 | -5.76 | -2.77E-05 | <0.0001 | ---NA--- |  |  |  |
| Lbot_4FC_C2847 | -5.19 | -1.47E-05 | 0.0001 | maltase A1-like | *Heliothis virescens* | GO:0003824; GO:0005975 | catalytic activity;carbohydrate metabolic process |
| Lbot_4FC_C3192 | -5.15 | -1.01E-05 | 0.0031 | myrosinase 1-like | *Heliothis virescens* | GO:0004553; GO:0005975 | hydrolase activity, hydrolyzing O-glycosyl compounds;carbohydrate metabolic process |
| Lbot_4FC_C11358 | -4.95 | -7.74E-06 | 0.0494 | cytochrome P450 4d2-like | *Danaus plexippus plexippus* | GO:0004497; GO:0005506; GO:0016705; GO:0020037; GO:0055114 | monooxygenase activity;iron ion binding;oxidoreductase activity, acting on paired donors, with incorporation or reduction of molecular oxygen;heme binding;oxidation-reduction process |
| Lbot_4FC_C37067 | -4.87 | -6.93E-05 | 0.0001 | arylphorin subunit beta-like | *Leguminivora glycinivorella* | GO:0005615; GO:0045735 | extracellular space;nutrient reservoir activity |
| Lbot_4FC_C5710 | -4.76 | -1.03E-05 | 0.0052 | myrosinase 1-like | *Heliothis virescens* | GO:0004553; GO:0005975 | hydrolase activity, hydrolyzing O-glycosyl compounds;carbohydrate metabolic process |
| Lbot_4FC_C3370 | -4.55 | -1.70E-05 | <0.0001 | uncharacterized oxidoreductase YrbE-like | *Galleria mellonella* | GO:0016491; GO:0055114 | oxidoreductase activity;oxidation-reduction process |
| Lbot_4FC_C145 | -4.21 | -3.02E-05 | <0.0001 | carboxypeptidase B-like | *Papilio xuthus* | GO:0004181; GO:0004930; GO:0006508; GO:0007186; GO:0008270; GO:0016021 | metallocarboxypeptidase activity;G protein-coupled receptor activity;proteolysis;G protein-coupled receptor signaling pathway;zinc ion binding;integral component of membrane |
| Lbot_4FC_C1968 | -4.19 | -9.63E-06 | 0.0199 | Endonuclease G, mitochondrial | *Spodoptera litura* | GO:0003676; GO:0004519; GO:0046872; GO:0090305 | nucleic acid binding;endonuclease activity;metal ion binding;nucleic acid phosphodiester bond hydrolysis |
| Lbot_4FC_C60039 | -4.05 | -2.03E-05 | 0.0001 | ---NA--- |  |  |  |
| Lbot_4FC_C1232 | -3.96 | -1.53E-05 | 0.0003 | mitochondrial enolase superfamily member 1-like | *Galleria mellonella* | GO:0009063; GO:0016052; GO:0050023 | cellular amino acid catabolic process;carbohydrate catabolic process;L-fuconate dehydratase activity |
| Lbot_4FC_C1058 | -3.94 | -2.25E-05 | <0.0001 | epidermal retinol dehydrogenase 2-like | *Vanessa tameamea* | GO:0016021; GO:0016491; GO:0055114 | integral component of membrane;oxidoreductase activity;oxidation-reduction process |
| Lbot_4FC_C2954 | -3.91 | -1.72E-05 | 0.0001 | 3-ketoacyl-CoA thiolase, mitochondrial-like | *Galleria mellonella* | GO:0016747 | transferase activity, transferring acyl groups other than amino-acyl groups |
| Lbot_4FC_C1721 | -3.82 | -9.55E-05 | <0.0001 | fatty acid-binding protein 2-like | *Papilio machaon* | GO:0005737; GO:0008289 | cytoplasm;lipid binding |
| Lbot_4FC_C3035 | -3.74 | -2.36E-05 | <0.0001 | beta-1,3-glucan-binding protein-like | *Vanessa tameamea* | GO:0004553; GO:0005975 | hydrolase activity, hydrolyzing O-glycosyl compounds;carbohydrate metabolic process |
| Lbot_4FC_C20712 | -3.59 | -2.39E-05 | <0.0001 | larval cuticle protein LCP-17-like | *Hyposmocoma kahamanoa* | GO:0042302 | structural constituent of cuticle |
| Lbot_4FC_C2121 | -3.56 | -1.96E-05 | 0.0001 | chondroitin proteoglycan 2-like | *Spodoptera exigua* | GO:0005576; GO:0006030; GO:0008061 | extracellular region;chitin metabolic process;chitin binding |
| Lbot_4FC_C1065 | -3.27 | -1.12E-05 | 0.0314 | sucrose-6-phosphate hydrolase-like | *Galleria mellonella* | GO:0004564; GO:0005737; GO:0005975 | beta-fructofuranosidase activity;cytoplasm;carbohydrate metabolic process |
| Lbot_4FC_C3183 | -3.12 | -2.16E-05 | 0.0002 | fatty acid-binding protein, liver-like | *Plutella xylostella* | 0 | 0 |
| Lbot_4FC_C1475 | -3.09 | -1.23E-05 | 0.0399 | C-type lectin 27kD | *Vanessa tameamea* | GO:0030246 | carbohydrate binding |
| Lbot_4FC_C60932 | -3.05 | -1.61E-05 | 0.0127 | larval cuticle protein 1-like | *Choristoneura fumiferana* | GO:0003677; GO:0005664; GO:0006260; GO:0042302 | DNA binding;nuclear origin of replication recognition complex;DNA replication;structural constituent of cuticle |
| Lbot_4FC_C8460 | -2.96 | -1.79E-05 | 0.0038 | glutathione S-transferase-like | *Choristoneura fumiferana* | GO:0004364; GO:0005515 | glutathione transferase activity;protein binding |
| Lbot_4FC_C1354 | -2.87 | -2.62E-05 | 0.0001 | larval cuticle protein LCP-17-like | *Plutella xylostella* | GO:0042302 | structural constituent of cuticle |
| Lbot_4FC_C3173 | -2.87 | -2.52E-05 | 0.0001 | alpha-amylase 1-like | *Amyelois transitella* | GO:0004556; GO:0005975; GO:0043169; GO:0103025 | alpha-amylase activity;carbohydrate metabolic process;cation binding;alpha-amylase activity (releasing maltohexaose) |
| Lbot_4FC_C5369 | -2.81 | -1.36E-05 | 0.0191 | fatty acid hydroperoxide dehydratase | *Papilio xuthus* | 0 | 0 |
| Lbot_4FC_C5213 | -2.8 | -1.86E-05 | 0.0035 | uncharacterized family 31 glucosidase KIAA1161-like | *Bombyx mori* | GO:0004553; GO:0005975 | hydrolase activity, hydrolyzing O-glycosyl compounds;carbohydrate metabolic process |
| Lbot_4FC_C4483 | -2.74 | -4.60E-05 | <0.0001 | fatty acid-binding protein, liver-like | *Plutella xylostella* | 0 | 0 |
| Lbot_4FC_C3345 | -2.42 | -2.24E-05 | 0.0062 | esterase FE4-like | *Cydia pomonella* | GO:0016787 | hydrolase activity |
| Lbot_4FC_C2051 | -2.34 | -2.60E-05 | 0.0027 | 3-ketoacyl-CoA thiolase, mitochondrial-like | *Bicyclus anynana* | GO:0003985 | acetyl-CoA C-acetyltransferase activity |
| Lbot_4FC_C1754 | -2.33 | -3.56E-05 | 0.0004 | larval cuticle protein 1-like | *Choristoneura fumiferana* | GO:0042302 | structural constituent of cuticle |
| Lbot_4FC_C60285 | -2.31 | -2.59E-05 | 0.014 | larval cuticle protein LCP-17-like | *Spodoptera litura* | GO:0042302 | structural constituent of cuticle |
| Lbot_4FC_C2735 | -2.25 | -2.09E-05 | 0.0199 | probable medium-chain specific acyl-CoA dehydrogenase, mitochondrial isoform X1 | *Plutella xylostella* | GO:0003995; GO:0050660; GO:0055114 | acyl-CoA dehydrogenase activity;flavin adenine dinucleotide binding;oxidation-reduction process |
| Lbot_4FC_C2437 | -2.2 | -2.38E-05 | 0.0118 | pancreatic triacylglycerol lipase-like | *Heliothis virescens* | GO:0005576; GO:0006629; GO:0052689 | extracellular region;lipid metabolic process;carboxylic ester hydrolase activity |
| Lbot_4FC_C2691 | -2.2 | -3.54E-05 | 0.001 | pancreatic triacylglycerol lipase-like | *Antheraea yamamai* | GO:0005576; GO:0006629; GO:0052689 | extracellular region;lipid metabolic process;carboxylic ester hydrolase activity |
| Lbot_4FC_C1667 | -2.14 | -3.55E-05 | 0.0016 | astacin-like metalloprotease toxin 5 | *Galleria mellonella* | GO:0004222; GO:0006508; GO:0008270 | metalloendopeptidase activity;proteolysis;zinc ion binding |
| Lbot_4FC_C826 | -2.08 | -3.20E-05 | 0.0062 | pancreatic triacylglycerol lipase-like | *Antheraea pernyi* | GO:0005576; GO:0006629; GO:0052689 | extracellular region;lipid metabolic process;carboxylic ester hydrolase activity |
| Lbot_4FC_C425 | -2.04 | -6.86E-05 | 0.0001 | zinc metalloproteinase nas-4-like | *Amyelois transitella* | GO:0004222; GO:0006508; GO:0008270 | metalloendopeptidase activity;proteolysis;zinc ion binding |
| Lbot_4FC_C5107 | -1.92 | -4.63E-05 | 0.0052 | endocuticle structural glycoprotein ABD-5-like | *Pieris rapae* | GO:0042302 | structural constituent of cuticle |
| Lbot_4FC_C363 | -1.84 | -3.30E-05 | 0.041 | chitin binding PM protein | *Papilio polytes* | GO:0004099; GO:0005975; GO:0051060 | chitin deacetylase activity;carbohydrate metabolic process;pullulanase activity |
| Lbot_4FC_C605 | -1.79 | -4.15E-05 | 0.0303 | pancreatic triacylglycerol lipase-like | *Papilio xuthus* | GO:0005576; GO:0052689 | extracellular region;carboxylic ester hydrolase activity |
| Lbot_4FC_C1402 | -1.75 | -4.74E-05 | 0.0345 | arginine kinase isoform X1 | *Helicoverpa armigera* | GO:0005524; GO:0016021; GO:0016301; GO:0016310 | ATP binding;integral component of membrane;kinase activity;phosphorylation |
| Lbot_4FC_C828 | -1.72 | -5.55E-05 | 0.0303 | acyl-CoA-binding protein homolog | *Plutella xylostella* | GO:0000062; GO:0008289 | fatty-acyl-CoA binding;lipid binding |
| Lbot_4FC_C1572 | 1.88 | 0.00017454 | 0.0001 | ---NA--- |  |  |  |
| Lbot_4FC_C70 | 2.09 | 0.00012163 | <0.0001 | cytochrome P450 4g15 | *Plutella xylostella* | GO:0004497; GO:0005506; GO:0016021; GO:0016705; GO:0020037; GO:0055114 | monooxygenase activity;iron ion binding;integral component of membrane;oxidoreductase activity, acting on paired donors, with incorporation or reduction of molecular oxygen;heme binding;oxidation-reduction process |
| Lbot_4FC_C42867 | 2.27 | 1.59E-05 | 0.0459 | probable Ras GTPase-activating protein isoform X6 | *Bicyclus anynana* | GO:0007165; GO:0043087 | signal transduction;regulation of GTPase activity |
| Lbot_4FC_C7299 | 2.48 | 1.63E-05 | 0.0303 | ---NA--- |  |  |  |
| Lbot_4FC_C2611 | 2.64 | 1.49E-05 | 0.0407 | juvenile hormone binding protein | *Spodoptera litura* |  |  |
| Lbot_4FC_C7222 | 2.79 | 1.26E-05 | 0.0459 | Tyrosine-protein kinase BAZ1B | *Amyelois transitella* | GO:0005488 | binding |
| Lbot_4FC_C7444 | 5.64 | 1.58E-05 | 0.0001 | fatty acid synthase | *Bombyx mori* | GO:0004312; GO:0009058; GO:0016491; GO:0016788; GO:0031177; GO:0055114 | fatty acid synthase activity;biosynthetic process;oxidoreductase activity;hydrolase activity, acting on ester bonds;phosphopantetheine binding;oxidation-reduction process |
| Lbot_4FC_C60370 | 6.38 | 8.82E-06 | 0.0029 | endocuticle structural glycoprotein SgAbd-5-like | *Helicoverpa armigera* | GO:0042302 | structural constituent of cuticle |
| Lbot_4FC_C11852 | 6.43 | 9.04E-06 | 0.0139 | ---NA--- |  |  |  |
| Lbot_4FC_C61342 | 6.98 | 9.51E-06 | 0.0011 | ---NA--- |  |  |  |
| Lbot_4FC_C30297 | 17.11 | 1.69E-05 | <0.0001 | basic juvenile hormone-suppressible protein 1-like | *Danaus plexippus plexippus* | GO:0005615; GO:0045735 | extracellular space;nutrient reservoir activity |
| Lbot_4FC_C42322 | 18.58 | 1.23E-05 | <0.0001 | basic juvenile hormone-suppressible protein 1-like | *Helicoverpa armigera* |  |  |
| Lbot_4FC_C71661 | 35.34 | 1.17E-05 | <0.0001 | basic juvenile hormone-suppressible protein 1-like | *Heliconius erato* | GO:0005615; GO:0045735 | extracellular space;nutrient reservoir activity |
| Lbot_4FC_C3539 | 45.23 | 7.29E-06 | 0.0005 | ---NA--- |  |  |  |

**Table S26:** Significantly differentially expressed contigs in *L. botrana* larvae feeding on inflorescences *vs.* véraison berries of ‘Cabernet Sauvignon’ under elevated CO_2_ concentration (EdgeR-analysis). A negative fold change indicates that the expression levels were lower in larvae feeding on véraison berries.

| Feature ID | Fold change | Weighted difference | FDR p-value correction | Sequence Description | Blast Top Hit Taxonomy Name | Annotation GO ID | Annotation GO Term |
| --- | --- | --- | --- | --- | --- | --- | --- |
| Lbot_4FC_C3539 | -125.08 | -1.54E-05 | <0.0001 | ---NA--- | 0 | 0 | 0 |
| Lbot_4FC_C705 | -13.66 | -3.10E-05 | <0.0001 | arylphorin precursor | *Leguminivora glycinivorella* | GO:0005615; GO:0045735 | extracellular space; nutrient reservoir activity |
| Lbot_4FC_C883 | -11.38 | -3.72E-05 | <0.0001 | pancreatic triacylglycerol lipase-like | *Bombyx mori* | GO:0005576; GO:0006629; GO:0052689 | extracellular region;lipid metabolic process;carboxylic ester hydrolase activity |
| Lbot_4FC_C173 | -8.86 | -9.15E-06 | 0.0058 | myrosinase 1-like | *Papilio polytes* | GO:0004553; GO:0005975 | hydrolase activity, hydrolyzing O-glycosyl compounds; carbohydrate metabolic process |
| Lbot_4FC_C11358 | -6.95 | -8.94E-06 | 0.0125 | cytochrome P450 4d2-like | *Danaus plexippus plexippus* | GO:0004497; GO:0005506; GO:0016705; GO:0020037; GO:0055114 | monooxygenase activity; iron ion binding; oxidoreductase activity, acting on paired donors, with incorporation or reduction of molecular oxygen; heme binding; oxidation-reduction process |
| Lbot_4FC_C63 | -6.58 | -9.02E-05 | <0.0001 | pancreatic triacylglycerol lipase-like | *Spodoptera litura* | GO:0005576; GO:0006629; GO:0052689 | extracellular region; lipid metabolic process; carboxylic ester hydrolase activity |
| Lbot_4FC_C882 | -5.94 | -1.29E-04 | <0.0001 | pancreatic triacylglycerol lipase-like | *Operophtera brumata* | GO:0005576; GO:0006629; GO:0052689 | extracellular region;lipid metabolic process;carboxylic ester hydrolase activity |
| Lbot_4FC_C2121 | -5.13 | -2.24E-05 | <0.0001 | chondroitin proteoglycan 2-like | *Spodoptera exigua* | GO:0005576; GO:0006030; GO:0008061 | extracellular region; chitin metabolic process; chitin binding |
| Lbot_4FC_C145 | -4.69 | -3.54E-05 | <0.0001 | carboxypeptidase B-like | *Papilio xuthus* | GO:0004181; GO:0004930; GO:0006508; GO:0007186; GO:0008270; GO:0016021 | metallocarboxypeptidase activity; G protein-coupled receptor activity; proteolysis; G protein-coupled receptor signaling pathway; zinc ion binding; integral component of membrane |
| Lbot_4FC_C2644 | -4.5 | -1.53E-05 | 0.0005 | cytochrome P450 6B6-like | *Hyposmocoma kahamanoa* | GO:0004497; GO:0005506; GO:0016705; GO:0020037; GO:0055114 | monooxygenase activity; iron ion binding; oxidoreductase activity, acting on paired donors, with incorporation or reduction of molecular oxygen; heme binding; oxidation-reduction process |
| Lbot_4FC_C270 | -4.46 | -3.68E-05 | <0.0001 | PREDICTED: uncharacterized protein LOC106709627 | *Papilio machaon* |  |  |
| Lbot_4FC_C1065 | -3.83 | -1.49E-05 | 0.0029 | sucrose-6-phosphate hydrolase-like | *Galleria mellonella* | GO:0004564; GO:0005737; GO:0005975 | beta-fructofuranosidase activity; cytoplasm; carbohydrate metabolic process |
| Lbot_4FC_C1721 | -3.62 | -7.40E-05 | <0.0001 | fatty acid-binding protein 2-like | *Papilio machaon* | GO:0005737; GO:0008289 | cytoplasm; lipid binding |
| Lbot_4FC_C1058 | -3.59 | -1.91E-05 | 0.0003 | epidermal retinol dehydrogenase 2-like | *Vanessa tameamea* | GO:0016021; GO:0016491; GO:0055114 | integral component of membrane; oxidoreductase activity; oxidation-reduction process |
| Lbot_4FC_C10825 | -3.57 | -1.25E-05 | 0.0102 | ---NA--- |  |  |  |
| Lbot_4FC_C2954 | -3.41 | -1.31E-05 | 0.0148 | 3-ketoacyl-CoA thiolase, mitochondrial-like | *Galleria mellonella* | GO:0016747 | transferase activity, transferring acyl groups other than amino-acyl groups |
| Lbot_4FC_C7581 | -3.23 | -1.20E-05 | 0.0381 | glutathione S-transferase D1 | *Cydia pomonella* | GO:0004364; GO:0005737; GO:0006749; GO:0018833 | glutathione transferase activity; cytoplasm; glutathione metabolic process; DDT-dehydrochlorinase activity |
| Lbot_4FC_C2847 | -3.13 | -1.29E-05 | 0.0375 | maltase A1-like | *Heliothis virescens* | GO:0003824; GO:0005975 | catalytic activity; carbohydrate metabolic process |
| Lbot_4FC_C3173 | -2.99 | -2.50E-05 | 0.0003 | alpha-amylase 1-like | *Amyelois transitella* | GO:0004556; GO:0005975; GO:0043169; GO:0103025 | alpha-amylase activity; carbohydrate metabolic process; cation binding; alpha-amylase activity (releasing maltohexaose) |
| Lbot_4FC_C4483 | -2.78 | -4.05E-05 | <0.0001 | fatty acid-binding protein, liver-like | *Plutella xylostella* |  |  |
| Lbot_4FC_C2437 | -2.21 | -2.45E-05 | 0.0258 | pancreatic triacylglycerol lipase-like | *Heliothis virescens* | GO:0005576; GO:0006629; GO:0052689 | extracellular region; lipid metabolic process; carboxylic ester hydrolase activity |
| Lbot_4FC_C278 | -2.07 | -6.38E-05 | 0.0003 | trypsin CFT-1-like | *Choristoneura fumiferana* | GO:0004252; GO:0005615; GO:0006508 | serine-type endopeptidase activity; extracellular space; proteolysis |
| Lbot_4FC_C57261 | -1.72 | -1.04E-04 | 0.0093 | ---NA--- |  |  |  |
| Lbot_4FC_C22002 | -1.64 | -7.63E-04 | 0.0056 | Cuticular protein glycine-rich 20 | *Vanessa tameamea* | GO:0098542 | defense response to other organism |
| Lbot_4FC_C13362 | 1.77 | 5.72E-05 | 0.0258 | ---NA--- |  |  |  |
| Lbot_4FC_C9606 | 1.87 | 5.63E-05 | 0.008 | ---NA--- |  |  |  |
| Lbot_4FC_C70 | 2.35 | 1.81E-04 | <0.0001 | cytochrome P450 4g15 | *Plutella xylostella* | GO:0004497; GO:0005506; GO:0016021; GO:0016705; GO:0020037; GO:0055114 | monooxygenase activity; iron ion binding; integral component of membrane; oxidoreductase activity, acting on paired donors, with incorporation or reduction of molecular oxygen; heme binding; oxidation-reduction process |
| Lbot_4FC_C2221 | 2.96 | 2.08E-05 | 0.0021 | diapausin precursor | *Spodoptera exigua* | GO:0005576; GO:0031640; GO:0042742; GO:0045087; GO:0050832 | extracellular region; killing of cells of other organism; defense response to bacterium; innate immune response; defense response to fungus |
| Lbot_4FC_C7444 | 3.71 | 1.55E-05 | 0.003 | fatty acid synthase | *Bombyx mori* | GO:0004312; GO:0009058; GO:0016491; GO:0016788; GO:0031177; GO:0055114 | fatty acid synthase activity; biosynthetic process; oxidoreductase activity; hydrolase activity, acting on ester bonds; phosphopantetheine binding; oxidation-reduction process |
| Lbot_4FC_C2029 | 3.8 | 1.22E-05 | 0.0198 | ---NA--- |  |  |  |
| Lbot_4FC_C10605 | 3.83 | 1.09E-05 | 0.0381 | ---NA--- |  |  |  |

**Table S27:** Significantly differentially expressed contigs in *L. botrana* larvae feeding on inflorescences of ‘Riesling’ vs. ‘Cabernet Sauvignon’ under ambient CO_2_ concentration (EdgeR-analysis). A negative fold change indicates that the expression levels were lower in larvae feeding on ‘Cabernet Sauvignon’.

| Feature ID | Fold change | Weighted difference | FDR p-value correction | Sequence Description | Blast Top Hit Taxonomy Name | Annotation GO ID | Annotation GO Term |
| --- | --- | --- | --- | --- | --- | --- | --- |
| Lbot_4FC_C1721 | 1.83 | 5.8704E-05 | 0.0362 | fatty acid-binding protein 2-like | *Papilio machaon* | GO:0005737; GO:0008289 | cytoplasm;lipid binding |
| Lbot_4FC_C1754 | 2.11 | 3.2814E-05 | 0.0285 | larval cuticle protein 1-like | *Choristoneura fumiferana* | GO:0042302 | structural constituent of cuticle |
| Lbot_4FC_C270 | 2.44 | 5.6062E-05 | <0.0001 | PREDICTED: uncharacterized protein LOC106709627 | *Papilio machaon* | 0 | 0 |
| Lbot_4FC_C705 | 3.19 | 8.0457E-05 | <0.0001 | arylphorin precursor | *Leguminivora glycinivorella* | GO:0005615; GO:0045735 | extracellular space;nutrient reservoir activity |
| Lbot_4FC_C2221 | 3.72 | 1.9003E-05 | 0.0006 | diapausin precursor | *Spodoptera exigua* | GO:0005576; GO:0031640; GO:0042742; GO:0045087; GO:0050832 | extracellular region;killing of cells of other organism;defense response to bacterium;innate immune response;defense response to fungus |
| Lbot_4FC_C1374 | 3.77 | 2.6237E-05 | <0.0001 | ---NA--- |  |  |  |
| Lbot_4FC_C1369 | 5.98 | 6.3945E-05 | <0.0001 | arylphorin precursor | *Leguminivora glycinivorella* | GO:0005615; GO:0045735 | extracellular space;nutrient reservoir activity |
| Lbot_4FC_C6445 | 8.58 | 0.00011766 | <0.0001 | arylphorin precursor | *Leguminivora glycinivorella* | GO:0005615; GO:0045735 | extracellular space;nutrient reservoir activity |
| Lbot_4FC_C86817 | 9.01 | 2.7396E-05 | 0.0109 | acidic juvenile hormone-suppressible protein 1-like | *Plutella xylostella* |  |  |
| Lbot_4FC_C37067 | 21.97 | 8.3263E-05 | <0.0001 | arylphorin subunit beta-like | *Leguminivora glycinivorella* | GO:0005615; GO:0045735 | extracellular space;nutrient reservoir activity |
| Lbot_4FC_C17381 | 32.11 | 1.3997E-05 | <0.0001 | methionine-rich storage protein | *Choristoneura fumiferana* |  |  |

**Table S28:** Significantly differentially expressed contigs in *L. botrana* larvae feeding on inflorescences of ‘Riesling’ vs. ‘Cabernet Sauvignon’ under elevated CO_2_ concentration (EdgeR-analysis). A negative fold change indicates that the expression levels were lower in larvae feeding on ‘Cabernet Sauvignon’.

| Feature ID | Fold change | Weighted difference | p-value (FDR corrected) | Sequence Description | Blast Top Hit Taxonomy Name | Annotation GO ID | Annotation GO Term |
| --- | --- | --- | --- | --- | --- | --- | --- |
| Lbot_4FC_C60369 | -30.1 | -6.64E-06 | 0.0079 | endocuticle structural glycoprotein SgAbd-5-like | *Trichoplusia ni* | GO:0042302 | structural constituent of cuticle |
| Lbot_4FC_C59726 | -19.23 | -1.19E-05 | <0.0001 | larval cuticle protein 8-like | *Galleria mellonella* | GO:0042302 | structural constituent of cuticle |
| Lbot_4FC_C60020 | -17.85 | -7.30E-06 | 0.0127 | ---NA--- |  |  |  |
| Lbot_4FC_C60039 | -16.6 | -1.14E-05 | 0.0001 | ---NA--- |  |  |  |
| Lbot_4FC_C32778 | -6.32 | -1.19E-05 | 0.0043 | ---NA--- |  |  |  |
| Lbot_4FC_C1813 | -6.25 | -9.47E-06 | 0.0151 | uncharacterized protein LOC113508223 | *Trichoplusia ni* | GO:0003676; GO:0015074 | nucleic acid binding; DNA integration |
| Lbot_4FC_C60097 | -4.5 | -1.69E-05 | 0.0004 | endocuticle structural glycoprotein ABD-4-like | *Hyposmocoma kahamanoa* | GO:0016021; GO:0042302 | integral component of membrane; structural constituent of cuticle |
| Lbot_4FC_C57137 | -3.66 | -1.75E-05 | 0.0014 | urbain precursor | *Galleria mellonella* |  |  |
| Lbot_4FC_C57261 | 1.87 | 1.17E-04 | 0.0009 | ---NA--- |  |  |  |
| Lbot_4FC_C22002 | 1.9 | 9.24E-04 | <0.0001 | Cuticular protein glycine-rich 20 | *Vanessa tameamea* | GO:0098542 | defense response to other organism |
| Lbot_4FC_C55 | 2.1 | 5.78E-05 | 0.0006 | flexible cuticle protein 12-like | *Nicrophorus vespilloides* | GO:0042302 | structural constituent of cuticle |
| Lbot_4FC_C4427 | 2.4 | 2.81E-05 | 0.0043 | larval cuticle protein LCP-17-like | *Papilio polytes* | GO:0042302 | structural constituent of cuticle |
| Lbot_4FC_C6321 | 3.3 | 1.28E-05 | 0.0461 | pupal cuticle protein 36-like | *Plutella xylostella* |  |  |
| Lbot_4FC_C10477 | 5.14 | 1.25E-05 | 0.0023 | ---NA--- |  |  |  |
| Lbot_4FC_C9736 | 7.39 | 1.44E-05 | 0.0001 | cuticle protein 1-like | *Papilio xuthus* | GO:0005576; GO:0016021 | extracellular region; integral component of membrane |
| Lbot_4FC_C23727 | 15.14 | 7.27E-06 | 0.0127 | ---NA--- |  |  |  |
| Lbot_4FC_C3540 | 22.25 | 8.18E-06 | 0.0043 | ---NA--- |  |  |  |

**Table S29:** Significantly differentially expressed contigs in *L. botrana* larvae feeding on véraison berries of ‘Riesling’ vs. ‘Cabernet Sauvignon’ under ambient CO_2_ concentration (EdgeR-analysis). A negative fold change indicates that the expression levels were lower in larvae feeding on ‘Cabernet Sauvignon’.

| Feature ID | Fold change | Weighted difference | FDR p-value correction | Sequence Description | Blast Top Hit Taxonomy Name | Annotation GO ID | Annotation GO Term |
| --- | --- | --- | --- | --- | --- | --- | --- |
| Lbot_4FC_C37066 | -9.41 | -1.2251E-05 | 0.0001 | arylphorin subunit beta-like | *Leguminivora glycinivorella* | GO:0005615; GO:0045735 | extracellular space;nutrient reservoir activity |
| Lbot_4FC_C6445 | -4.36 | -7.3924E-05 | 0.0031 | arylphorin precursor | *Leguminivora glycinivorella* | GO:0005615; GO:0045735 | extracellular space;nutrient reservoir activity |
| Lbot_4FC_C37067 | -4.25 | -5.8247E-05 | 0.0146 | arylphorin subunit beta-like | *Leguminivora glycinivorella* | GO:0005615; GO:0045735 | extracellular space;nutrient reservoir activity |
| Lbot_4FC_C25179 | 1.58 | 0.00356421 | 0.0276 | predicted protein | *Nematostella vectensis* |  |  |
| Lbot_4FC_C42867 | 4.25 | 2.1698E-05 | <0.0001 | probable Ras GTPase-activating protein isoform X6 | *Bicyclus anynana* | GO:0007165; GO:0043087 | signal transduction;regulation of GTPase activity |
| Lbot_4FC_C71661 | 8.60 | 1.0673E-05 | 0.0036 | basic juvenile hormone-suppressible protein 1-like | *Heliconius erato* | GO:0005615; GO:0045735 | extracellular space;nutrient reservoir activity |
| Lbot_4FC_C42322 | 11.62 | 1.1906E-05 | 0.0002 | basic juvenile hormone-suppressible protein 1-like | *Helicoverpa armigera* |  |  |
| Lbot_4FC_C30297 | 12.82 | 1.6557E-05 | <0.0001 | basic juvenile hormone-suppressible protein 1-like | *Danaus plexippus plexippus* | GO:0005615; GO:0045735 | extracellular space;nutrient reservoir activity |
| Lbot_4FC_C60932 | 13.21 | 7.2601E-06 | 0.0046 | larval cuticle protein 1-like | *Choristoneura fumiferana* | GO:0003677; GO:0005664; GO:0006260; GO:0042302 | DNA binding;nuclear origin of replication recognition complex;DNA replication;structural constituent of cuticle |
| Lbot_4FC_C20712 | 13.77 | 8.5714E-06 | 0.0036 | larval cuticle protein LCP-17-like | *Hyposmocoma kahamanoa* | GO:0042302 | structural constituent of cuticle |
| Lbot_4FC_C17381 | 15.14 | 2.0795E-05 | <0.0001 | methionine-rich storage protein | *Choristoneura fumiferana* |  |  |
| Lbot_4FC_C60285 | 15.19 | 1.8465E-05 | <0.0001 | larval cuticle protein LCP-17-like | *Spodoptera litura* | GO:0042302 | structural constituent of cuticle |
| Lbot_4FC_C22063 | 23.82 | 6.1824E-06 | 0.0079 | cuticle protein 3-like | *Galleria mellonella* | GO:0042302 | structural constituent of cuticle |
| Lbot_4FC_C59726 | 26.53 | 7.6897E-06 | 0.0032 | larval cuticle protein 8-like | *Galleria mellonella* | GO:0042302 | structural constituent of cuticle |
| Lbot_4FC_C41869 | 26.83 | 6.5303E-06 | 0.0032 | endocuticle structural glycoprotein SgAbd-5-like | *Papilio machaon* | GO:0016021; GO:0042302 | integral component of membrane;structural constituent of cuticle |
| Lbot_4FC_C60353 | 32.91 | 6.5724E-06 | 0.0032 | ---NA--- |  |  |  |
| Lbot_4FC_C36705 | 34.78 | 1.2336E-05 | <0.0001 | endocuticle structural glycoprotein SgAbd-8-like | *Trichoplusia ni* | GO:0042302 | structural constituent of cuticle |
| Lbot_4FC_C51784 | 38.37 | 6.3397E-06 | 0.0046 | ---NA--- |  |  |  |
| Lbot_4FC_C59790 | 41.25 | 1.1031E-05 | <0.0001 | glycine-rich cell wall structural protein | *Papilio machaon* |  |  |
| Lbot_4FC_C60370 | 44.69 | 1.0228E-05 | <0.0001 | endocuticle structural glycoprotein SgAbd-5-like | *Helicoverpa armigera* | GO:0042302 | structural constituent of cuticle |
| Lbot_4FC_C60020 | 49.98 | 6.0653E-06 | 0.0079 | ---NA--- |  |  |  |
| Lbot_4FC_C60039 | 53.71 | 6.5277E-06 | 0.0046 | ---NA--- |  |  |  |
| Lbot_4FC_C61342 | 55.26 | 1.0895E-05 | <0.0001 | ---NA--- |  |  |  |
| Lbot_4FC_C60865 | 61.77 | 7.5256E-06 | 0.0011 | ---NA--- |  |  |  |
| Lbot_4FC_C60369 | 77.95 | 9.5294E-06 | <0.0001 | endocuticle structural glycoprotein SgAbd-5-like | *Trichoplusia ni* | GO:0042302 | structural constituent of cuticle |
| Lbot_4FC_C60097 | 148.28 | 1.8239E-05 | <0.0001 | endocuticle structural glycoprotein ABD-4-like | *Hyposmocoma kahamanoa* | GO:0016021; GO:0042302 | integral component of membrane;structural constituent of cuticle |

**Table S30:** Significantly differentially expressed contigs in *L. botrana* larvae feeding on véraison berries of ‘Riesling’ vs. ‘Cabernet Sauvignon’ under elevated CO_2_ concentration (EdgeR-analysis). A negative fold change indicates that the expression levels were lower in larvae feeding on ‘Cabernet Sauvignon’.

| Feature ID | Fold change | Weighted difference | FDR p-value correction | Sequence Description | Blast Top Hit Taxonomy Name | Annotation GO ID | Annotation GO Term |
| --- | --- | --- | --- | --- | --- | --- | --- |
| Lbot_4FC_C3539 | -77.48 | -9.47E-06 | 0.0001 | ---NA--- |  |  |  |
| Lbot_4FC_C85940 | -48.34 | -1.35E-05 | <0.0001 | osiris 9E | *Hyposmocoma kahamanoa* | GO:0016021 | integral component of membrane |
| Lbot_4FC_C86817 | -46.26 | -6.70E-06 | 0.0046 | acidic juvenile hormone-suppressible protein 1-like | *Plutella xylostella* |  |  |
| Lbot_4FC_C6445 | -31.38 | -1.20E-05 | <0.0001 | arylphorin precursor | *Leguminivora glycinivorella* | GO:0005615; GO:0045735 | extracellular space; nutrient reservoir activity |
| Lbot_4FC_C37067 | -30.16 | -8.80E-06 | 0.0001 | arylphorin subunit beta-like | *Leguminivora glycinivorella* | GO:0005615; GO:0045735 | extracellular space; nutrient reservoir activity |
| Lbot_4FC_C53688 | -10.26 | -9.90E-06 | 0.0059 | osiris 9F | *Vanessa tameamea* | GO:0016021 | integral component of membrane |
| Lbot_4FC_C55 | 1.93 | 4.22E-05 | 0.0357 | flexible cuticle protein 12-like | *Nicrophorus vespilloides* | GO:0042302 | structural constituent of cuticle |
